# Single-Molecule and π–π-Stacked Dimer Electron Transport in Carbazole and Folded Bicarbazole Derivatives in Molecular Junctions

**DOI:** 10.1021/acsomega.5c08254

**Published:** 2025-10-27

**Authors:** Adel Amer Alrehaili, Ross J. Davidson, Juan Hurtado-Gallego, Asma Alajmi, Noorah Alwhaibi, Andrei S. Batsanov, Santiago Martin, Pilar Cea, Martin R. Bryce, Nicolás Agraït, Ali K. Ismael, Colin J. Lambert

**Affiliations:** a Department of Physics, 4396 University of Lancaster, Lancaster LA1 4YB, United Kingdom; b Physics Department, Faculty of Science, Islamic University of Madinah, Madinah 42351, Saudi Arabia; c Department of Chemistry, 3057 Durham University, Durham DH1 3LE, United Kingdom; d Departamento de Física de la Materia Condensada C−III, and Instituto Universitario de Ciencia de Materiales “Nicolás Cabrera”, Universidad Autónoma de Madrid, Madrid E-28049, Spain; e Department of Physics, College of Science and Humanities in Al-Kharj, Prince Sattam Bin Abdulaziz University, Al-Kharj 11942, Saudi Arabia; f Instituto de Nanociencia y Materiales de Aragón (INMA), CSIC-Universidad de Zaragoza, Zaragoza 50009, Spain; g Departamento de Química Física, Facultad de Ciencias, 16765 Universidad de Zaragoza, Zaragoza 50009, Spain; h Laboratorio de Microscopias Avanzadas (LMA), 16765 Universidad de Zaragoza, Zaragoza 50018, Spain; i Department of Physics, College of Education for Pure Science, Tikrit University, Tikrit 34001, Iraq

## Abstract

The present work provides insight into how the conformations of flexible molecules can impact their single-molecule conductance. Six thiol-substituted carbazole-based molecules are synthesized and characterized. In four, two carbazole groups are joined by a linking group (1,3-propane or *meta*-xylene) while the remaining two are model monocarbazoles. Using a combination of X-ray photoelectron spectroscopy (XPS), single-molecule conductance measurements, and density functional theory (DFT) calculations, we demonstrate that upon transitioning from a self-assembled monolayer (SAM) to a single-molecule junction, the intermolecular interactions give way to intramolecular interactions. This resulted in the flexible bicarbazole molecular wire switching conductance mechanisms, which occurred primarily via the covalent conjugated aromatic part of the molecule in the SAM to one including conductance via noncovalent π–π interactions in the single-molecule junction.

## Graphical abstract

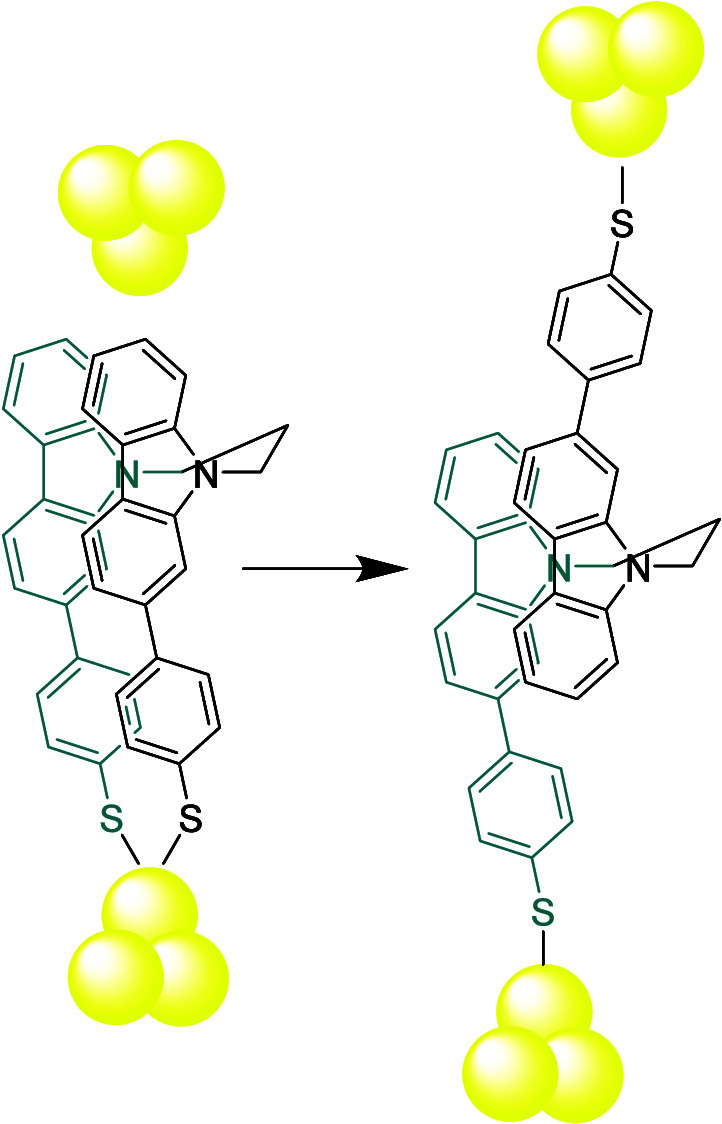

## Introduction

The field of molecule electronics has matured to the point where it is possible to examine structure–property relationships,^1–4^ not only providing information about single molecules, but also providing insight into more complex systems, such as self-assembled monolayers (SAMs) or thin films.^5–9^ Film morphology often plays an important role for conductive polymers, as small variations can significantly alter electron/hole transport.^10^ For example, altering the way polymer chains are packed influences the through-space conductance resulting from π–π interactions. Such conductance features are observed when studying the single-molecule conductance of planar aromatic molecular wires, due to the formation of face-to-face π–π stacked dimers during the course of junction evolution.^11–15^ (Subsequent discussion of π-stacked dimers refers to face-to-face π-π interactions, unless stated otherwise). However, due to their transient nature and/or the low probability of their occurrence, conductance via π-stacked dimers had not been thoroughly examined until the development of molecular junctions comprising two 1D molecular wires, each with only one surface binding group, joined by a rigid tether (e.g., phenyl, [2.2]­paracyclophane or xanthene) with a geometrical arrangement that promotes π-stacking.^16–21^ These studies demonstrated how increasing the area of the π–π overlap increases through-space conductance and confirmed the presence of destructive quantum interference (DQI) features.^16^ Owing to the spring-like nature of these molecules, Stefani et al. were able to demonstrate that the DQI feature could be mechanically manipulated, leading to variations in the conductance of the molecule as a function of strain.^18^ Beyond its effect on molecular conductance, through-space π–π conductance has also been predicted to be particularly relevant to the field of thermoelectricity. For example, Grace et al. highlighted how enhancing quantum interference in a molecular wire increases the magnitude of the Seebeck coefficient,^22^ while Wang et al. used thin films of porphyrins to demonstrate that through-space π-conductance can be used to screen phonons and thereby enhance thermal conductance.^23^ In each of the aforementioned examples, the tethers used to induce π–π stacking were rigid. In contrast, here, we seek to examine if through-space π–π conductance can be enhanced by using a flexible tether between the two π systems with a large enough aromatic area to promote π–π overlap. Additionally, the use of a flexible tether means that it is necessary to distinguish between linear strain and torsional twisting. Considering these criteria, carbazole-based systems were chosen as optimal candidates, as an alkane linker at the *N*-position can form a bicarbazole derivative. Additionally, carbazoles can be readily substituted at both the 2 or 3 positions (i.e., *meta* or *para* to the N atom, respectively; see Figure 1) with an anchor group to provide either a linear or bent molecule when π–π stacking is induced.

**Figure 1:**
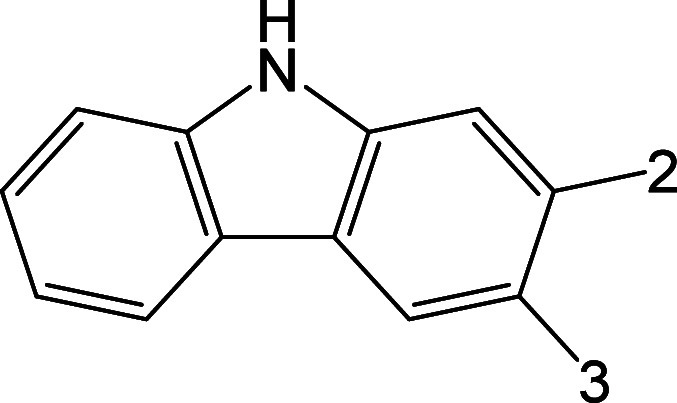
Carbazole substitution positions.

## Synthesis

DFT calculations showed that 1,3-propyl was the optimal alkane length to be used as a linker for a substituted bicarbazole system to promote intramolecular π–π overlap (SI, section 9). Using this information, the ethyl-trimethylsilyl protected compounds 1,3-bis­(2-(4-((2-(trimethylsilyl)­ethyl)­thio)­phenyl)-carbazol-9-yl)­propane (**1a-TMS**) and 1,3-bis­(3-(4-((2-(trimethylsilyl)­ethyl)­thio)­phenyl)-carbazol-9-yl)­propane (**1b-TMS**) were prepared by a combination of alkylation reactions with 1,3-dibromoporopane and Suzuki–Miyaura couplings with (4-((2-(trimethylsilyl)­ethyl)­thio)­phenyl)­boronic acid. The order in which these reactions were performed significantly impacted the purification of species and differed for each of the compounds. As such, the overall isolated yield of the compounds was dependent on the method used (see Supporting Information). Compounds **1a-TMS** and **1b-TMS** were converted to their thioacetate analogues **1a** and **1b** to facilitate gold|molecule|gold junction formation via the thiolate anchor groups.

In addition to compounds **1a** and **1b**, monocarbazoles substituted with a 1-propyl chain at the *N*-position (**2a** and **2b**) were prepared as controls to assess the impact of the second tethered carbazole, and finally, an additional pair of bicarbazole compounds was synthesized (**3a** and **3b**) using a *meta*-xylene tether instead of propyl to provide a more rigid linking unit as a comparison (Figure 2). *Meta*-xylene has been previously used in naphthalenetetracarboxylic systems to favor π–π stacking between molecules by maintaining a distance and geometry that would promote π-orbital overlap.^24–27^

**Figure 2:**
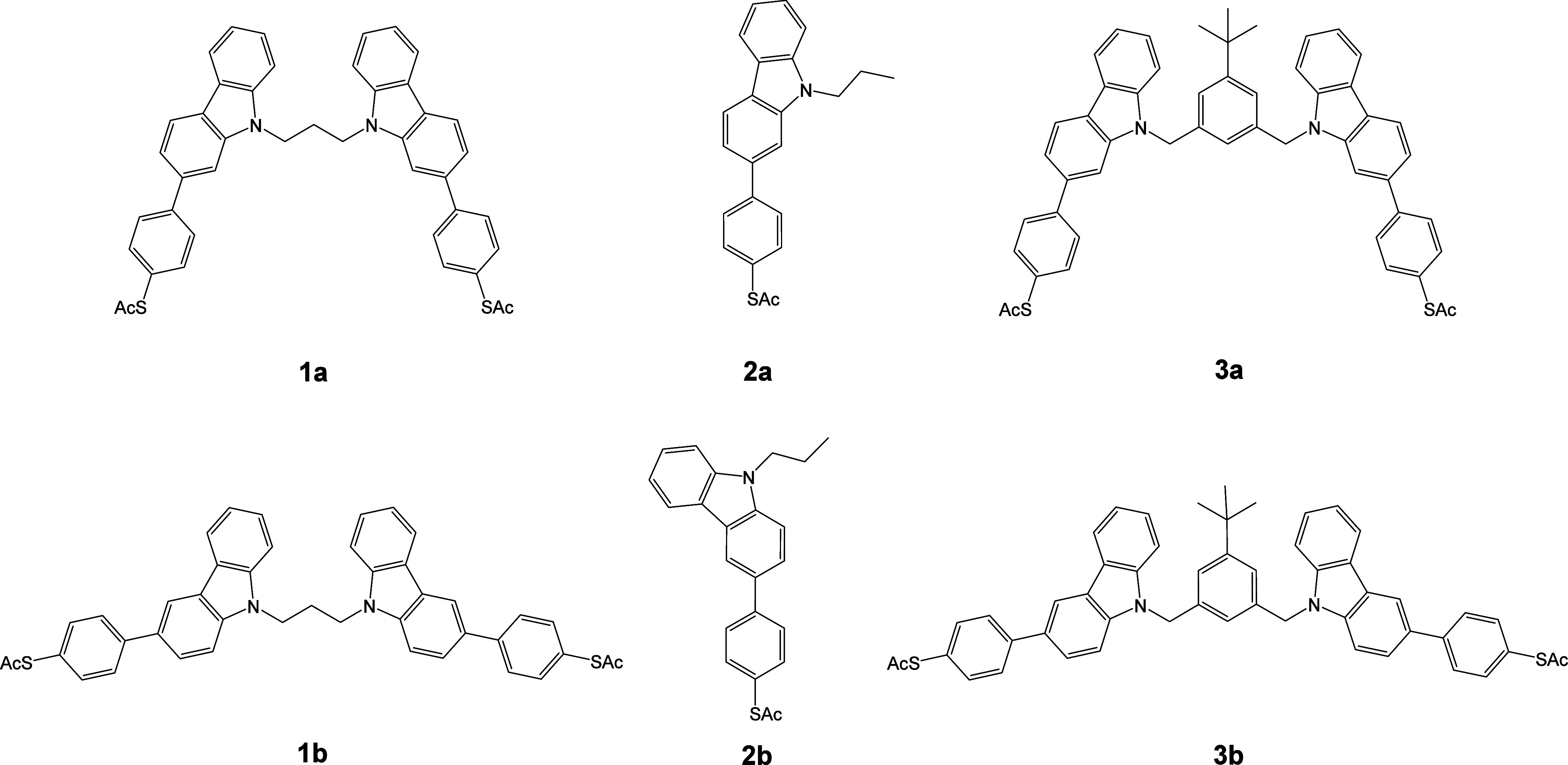
Structures of compounds **1–3a** and the isomeric series **1–3b** examined in this study.

### Structural Characterization

Crystals suitable for single-crystal X-ray diffraction of the precursor compounds **1a-TMS** and **3b-TMS** and the final compound **2a** were analyzed (CCDC numbers 2388669–2388670). Both **1a-TMS** (Figure 3 and Figures S39 and S42a) and **3b-TMS** (see SI, Figures S40 and S42b) show the carbazoles in an open conformation with packing dominated by π–H interactions, with no evidence of intra- or intermolecular π–π interactions. **2a** (see SI, Figure S41) exhibits similar behavior, showing no evidence of π–π interactions. A Cambridge Structural Database (CSD) survey for carbazoles with −CH_2_–C­(any substituents) group at N and no sterically hindering substituents in peri-positions (to N) returned 1000 entries exactly. Of these, 8% contain pairs of carbazole moieties stacked face-to-face with large overlap (π–π dimers), with just one structure showing an endless stack of carbazoles.^28^ This is a large sample size, so the 8% frequency is statistically significant. The prevailing motifs are either edge-to-face contacts (σ-π, or H-π) between carbazoles or between a carbazole and another aromatic group or π–π stacking between carbazole and another aromatic moiety. This suggests that the “open” conformation of these structures is favored.

**Figure 3:**
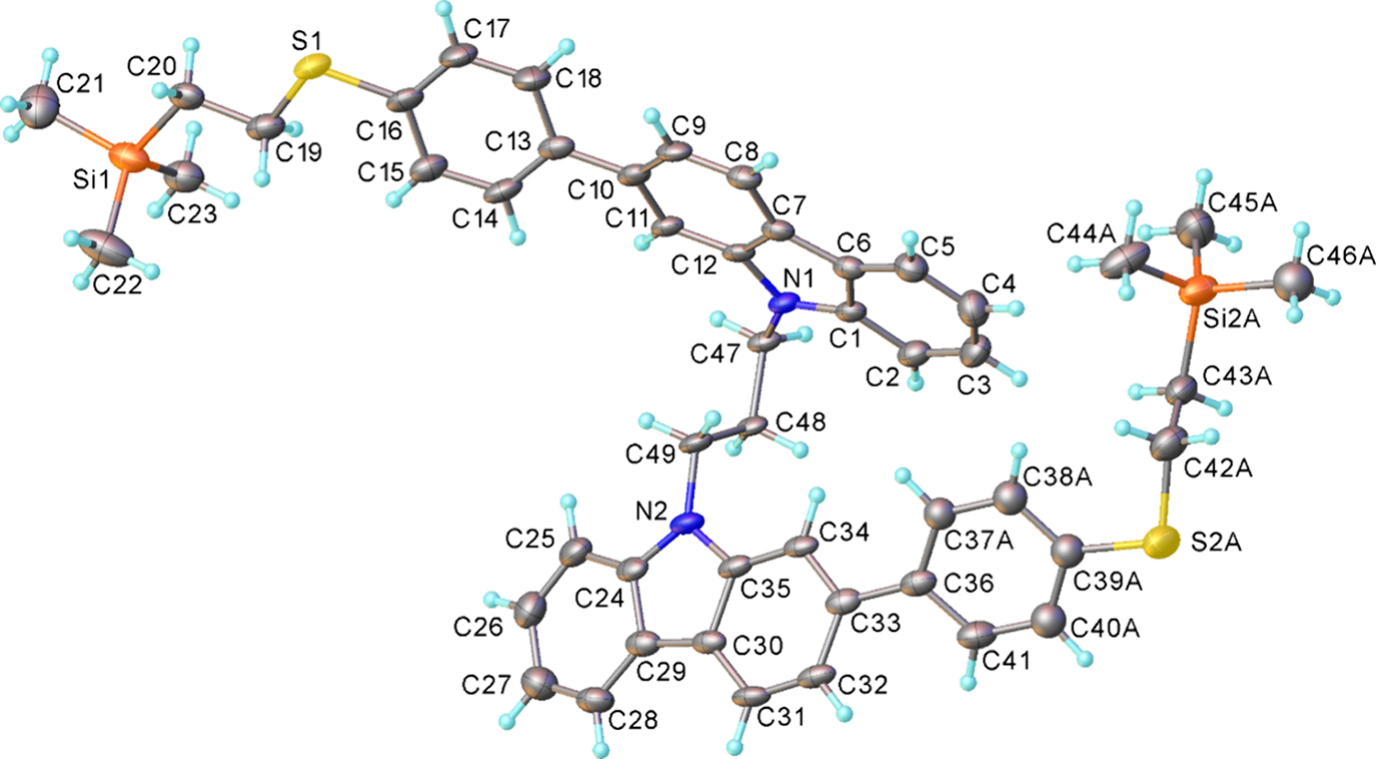
X-ray molecular structure of **1a-TMS**. Atomic displacement ellipsoids are drawn at the 50% probability level, and disorder is omitted for clarity.

### Conductance Measurements

Conductance (*G*) measurements were performed using a modified home-built scanning tunnelling microscope (STM) at ambient conditions and room temperature using the STM break junction (STM-BJ) technique.^29^ The conductance-distance traces (*GZ*) were obtained by recording the current while the tip is retracting to the sample after creating an electrical contact. A nonsupervised clustering technique^30–32^ “k-means” was used to separate *GZ* traces with similar conductance behavior. The conductance plateaus of each cluster were used to construct the 1D *G* histograms in Figure 4. To obtain mean conductance values (*G*_*m*_), Gaussian distributions were fitted to each peak of the resulting histograms.

**Figure 4:**
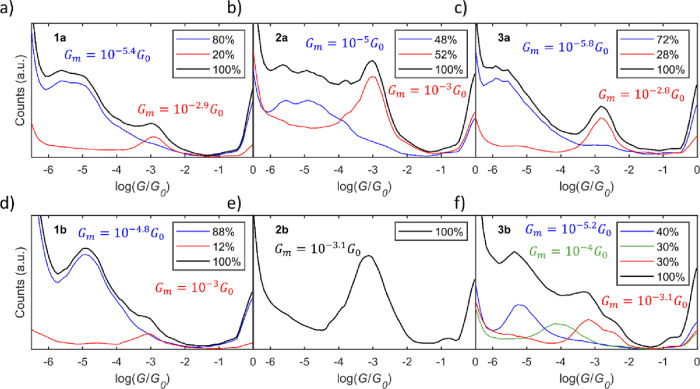
1D *G* histograms of all compounds. The black lines are the histograms of all traces with molecular junctions, and the colored lines are the histograms of the different *G* plateaus obtained using the clustering technique. The percentage of traces included in each cluster is included in the legend of each panel. The mean conductance values of each 1D *G* peak for each cluster are represented by *G*_*m*_ in its respective color.

All compounds displayed multiple conductance plateaus, except compound **2b**, and representative 1D *G* histograms are shown in Figure 4, along with their percentage traces. Except for the monocarbazole compounds (**2a** and **2b**), low-conductance plateaus are shown to be the most probable ones. Larger apparent stretching lengths obtained for low-conductance plateaus indicate a configuration of a completely extended molecule in the junction (see SI for further information).

Starting with the simplest compound **2a** (linear, rigid, single thiol), we postulate that the low-conductance (LC) plateaus (*G*_*m*_ = 10^–5^ *G*_0_) are attributed to a junction formed between two π-stacked molecules, with each bound to one of the electrodes by a thiol. Similar junctions have been previously observed for oligo­(*p*-phenyleneethynylene) molecular wires by Frisenda et al.^11^ The high-conductance (HC) plateaus (*G*_*m*_ = 10^–3^ *G*_0_) are attributed to a single **2a** molecule in the junction, in contact with one electrode via the thiol and the other via carbazole, similar to the proposed contact motif of porphyrins.^33^

Unlike **2a**, **2b** has only a single plateau (10^–3.1^ *G*_0_) similar to the high-conductance feature of **2a**, suggesting that **2b** does not form a π-stacked dimer in the junction. This can be explained by the difference in the molecular length of the isomers **2a** and **2b**. The molecular lengths of **2a** and **2b** are 14 and 13 Å, respectively. This length difference is also accompanied by a more favorable upright conformation of **2a**, which favors π–π stacking. Therefore, with a linear retraction of the STM tip, the longer molecule (**2a**) is afforded a greater probability to form the proposed π-stacked dimer. A more extreme example of this occurs when comparing the conductance behavior of the linear molecules 4-((4-(phenylethynyl)­phenyl)­ethynyl)­benzenethiol and 4-(phenylethynyl)­benzenethiol, for which the molecular lengths are 19.8 and 12.7 Å.^12^

Beyond the presence of multiple conductance plateaus observed for compounds **1a**, **1b**, **2a**, **3a**, and **3b**, the individual *GZ* traces of compounds **1a**, **1b**, and **2a** show a dramatic increase in conductance as the junction is extended, followed by a gradual decrease until the junction is broken (see Figure 5 for **1a** and Figures S50 and S51 for **1b** and **2a**). Oscillating conductances have previously been observed for spring-like cyclophane molecules resulting from the mechanical perturbation of a destructive quantum interference feature, due to a “stick–slip” motion of the molecule along one of the electrodes.^18^ In this previous work, the conductance plateaus appear with multiple conductance oscillations, while in the present case of the carbazole compounds, individual conductance increases and decreases are observed for most of the traces. No clear stick–slip behavior is observed in the carbazole junctions. Compound **1a** has a relatively higher probability of displaying conductance fluctuations (24.5%), whereas compounds **2a** and **1b** display this feature for only 10 and 5% of the junctions formed. In the following analysis, we will focus on **1a** (analysis for compounds **2a** and **1b** can be found in SI). *GZ* traces with conductance fluctuations were manually selected from all of the molecular traces. Figure 5a shows individual *GZ* traces of compound **1a**, where the conductance increase is clearly present. Red and black crosses on top of each trace indicate the minimum and maximum in *G*, respectively, where the conductance fluctuation occurs. The zero displacement of all *GZ* traces with a *G* fluctuation is centered at the start of the *G* increase (red cross). Figure 5b shows all of the selected *GZ* traces, with *G* fluctuations of compound **1a**, and on top of it, as a black line, the mean trace of the *G* value in this displacement range. A clear sharp increase followed by a smoother decrease in *G* is observed for the mean trace, indicating a clear mechanosensitive behavior of the molecule. To further explore the mean conductance fluctuation, we calculated the mean *G* value between the minimum and the maximum value of all the selected traces (shown in Figure S49 as Δ*G*). A variation of Δ*G* = 1.1 log (*G*/*G*_0_) was obtained for compounds **1a** and **1b**, while the variation for compound **2a** is Δ*G* = 0.8 log (*G*/*G*_0_). We also show this *G* fluctuation by comparing the normalized mean *GZ* trace of all of the selected traces with *G* fluctuations (see SI, Figure S52). Here, it is observed that not only is the *G* variation for compound **2a** smaller than the ones for compounds **1a** and **1b**, but also the distance of the fluctuation is smaller for compound **2a** (see SI for more information). This difference in the conductance fluctuations indicates a different origin between the conductance fluctuations of compounds **1a**, **1b**, and **2a**. Our hypothesis is that the *G* fluctuations observed in compound **2a** originate from the sliding motion of two molecules interacting through their π-systems. As previously studied, these oscillations typically have a distance periodicity of around 0.2 nm.^11^ The conductance fluctuations for compounds **1a** and **1b** may be the result of a conformation change as the junction is extended, given the flexible nature of compounds **1a** and **1b**, as has been observed by Wu et al., where 1,2-bis­(4-(methylthio)­phenyl)­ethane-1,2-dione was extended in a junction forcing a change between the *syn*- and *anti*-conformations.^34^

**Figure 5:**
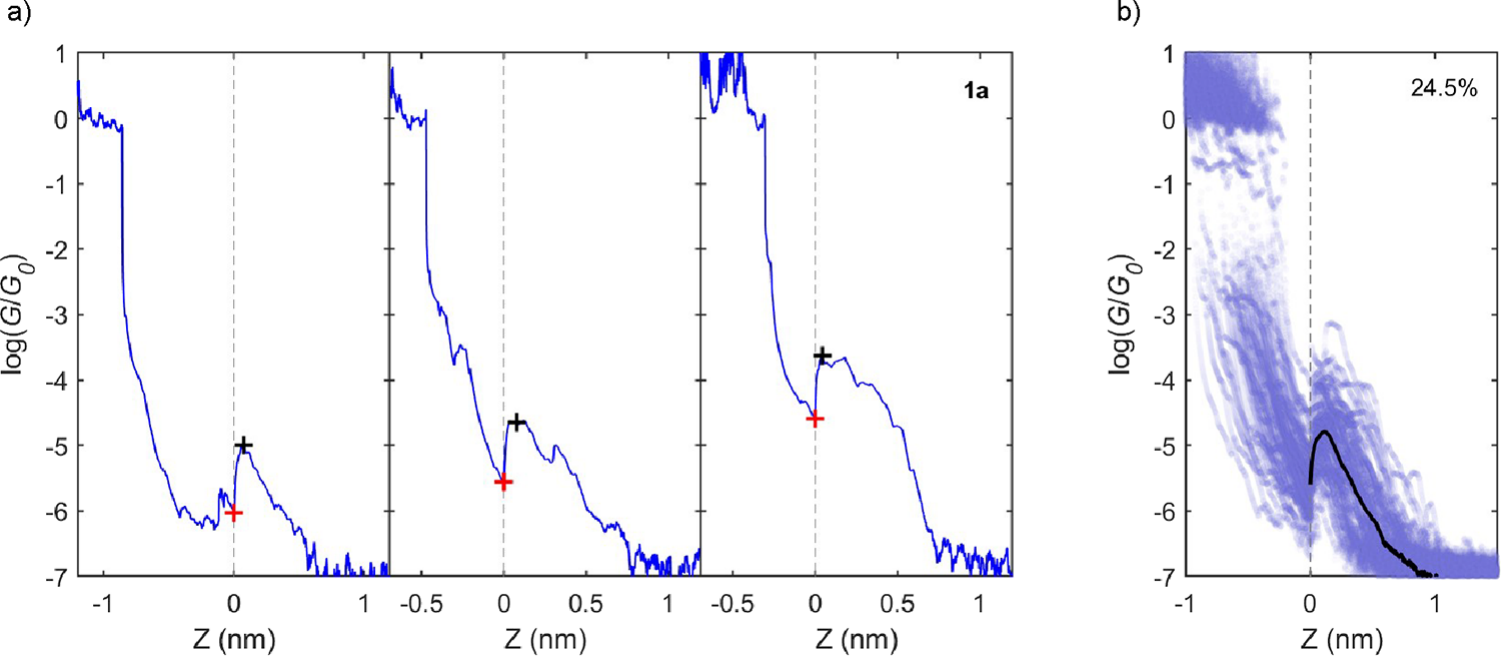
(a) Individual *GZ* traces of compound **1a** exhibiting a conductance fluctuation. Red and black crosses show the minimum and maximum *G* points of fluctuation, respectively. (b) All *GZ* traces with *G* fluctuations centered at the minimum *G* value of the fluctuation of compound **1a**. Percentage of traces with *G* fluctuations is shown in the top right part of the panel. Black trace represents the mean *G* versus displacement behavior of all the selected traces.

To gain insight into how the molecular conformations evolve as the junction is stretched, the initial state of each of the molecules on the gold substrate needs to be well-defined; therefore, X-ray photoelectron spectroscopy (XPS) and quartz crystal microbalance (QCM) analysis were performed.

Self-assembled monolayer (SAM) formation for all of the compounds was monitored by incubating a QCM resonator in a 1 mM solution in dichloromethane (DCM) and following its frequency with the incubation time. After 24 h, no further frequency variation was observed indicating the stable formation of the monolayer. The surface coverage of the resulting SAMs is shown in Table 1 according to the Sauerbrey equation.^35^

**Table 1:** Surface Coverage Values of the SAMs Formed by Compounds 1-**3a** and 1-**3b**, Determined by Quartz Crystal Microbalance Measurements

compound	surface coverage, Γ (molecules cm^–2^)
**1a**	0.9 × 10^14^
**1b**	0.8 × 10^14^
**2a**	2.1 × 10^14^
**2b**	1.5 × 10^14^
**3a**	0.9 × 10^14^
**3b**	0.9 × 10^14^

As shown in Table 1, very similar surface coverage was obtained for the double-thiol compounds **1a**, **1b**, **3a**, and **3b** suggesting the same orientation and arrangement of the molecules in the SAM even though compounds **1a** and **1b** are more flexible than the others. Nevertheless, approximately twice the surface coverage was observed for compounds **2a** and **2b** compared to **1a**, **1b**, **3a**, and **3b**, which can be attributed to **2a** and **2b** consisting of only one carbazole per molecule while the others contain two. Additionally, **2a** has a higher coverage than **2b**, which is likely due to the slightly different geometry of the molecules on the surface with **2a** having a “linear” structure while **2b** is “bent”.

XPS was utilized to determine the molecular orientation in the SAM formation for all compounds. For that, XPS measurements were performed on both powdered samples and SAMs on gold.

The powdered samples in the S 2p region (see SI, Figure S53) of the XPS spectra display two peaks at 163.5 and 164.7 eV for **2a** and **2b** and at 163.9 and 165.1 eV for **1a**, **1b**, **3a**, and **3b**. These peaks, separated by 1.2 eV with an area ratio of 2:1, are assigned to the (2p_3/2_) and (2p_1/2_) spin–orbit components, respectively. In contrast, the XPS spectra of the SAMs of all compounds formed on gold substrates exhibit only peaks at 161.9 and 163.1 eV, which arise from thiols chemisorbed to the gold substrate (see SI, Figure S53).^36–38^ Therefore, these data show that in the SAM, each molecule is in contact with the gold substrate through all of the available thiols present in each compound. However, the N 1s region of the SAM spectra shows a peak at the same binding energy as in the corresponding powder samples, indicating that there is no direct chemical bonding between the carbazole and the gold substrate (see SI, Figure S54).

Finally, none of the SAMs displayed evidence in the C 1s region (ca. 292 eV) that could be attributed to π–π stacking of the carbazoles, as was observed for **1a**, **1b**, **3a**, and **3b** powders but not for **2a** and **2b** (see SI, Figure S55), indicating that, as observed in the SCXRD data, these compounds tend not to have π–π interactions in the SAM leaving only H−π and van der Waals interactions to drive assembly. Taken together, these results show that in SAMs, compounds **1a**, **1b**, **3a**, and **3b** bind to the gold substrate via all available thiols in an “open” conformation (e.g., for **1b**, see Figure 6(iii)) while compounds **2a** and **2b** bind via the single thiol resulting in the molecule being vertically orientated relative to the substrate. Given the similar preparation conditions for the SAMs and samples used for conductance measurements, it is reasonable to assume that the SAMs provide an accurate representation of the molecules’ interaction with the gold surface prior to junction formation.

**Figure 6:**
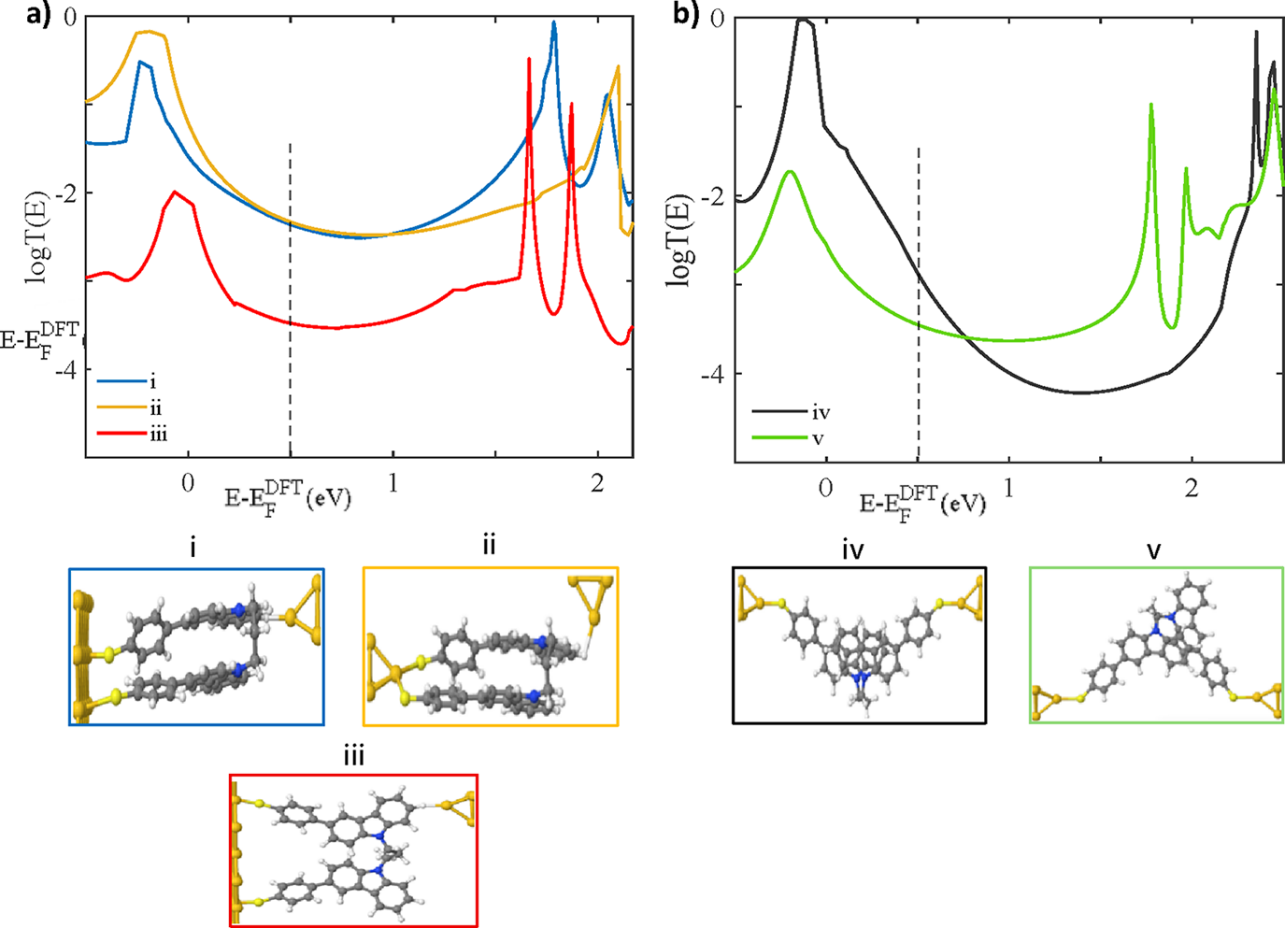
Transmission coefficient of **1b** for different bridging geometries. (a) (i,ii) closed-asym and (iii) open-asym. (b) (iv) Closed-asym, (v) open-asym. *T*(*E*) values are taken at *E* – *E*_*F*_^DFT^ = 0.5 eV.

### Computational Modeling and Interpretation

To explain each of the conductance features and the evolution of the junction (i.e., the switching events), we turned to DFT calculations. Theories of electron transport in single-molecule junctions are based on the concept that electrons moving through a molecule from the source electrode to the drain electrode maintain coherence, and their energy *E* remains unchanged during transit. Thus, the conductance *G* of the molecular junction is described by the Landauer formula *G* = *G*_0_ *T*(*E*_*F*_), where *G*_0_ represents the quantum of conductance and *T*(*E*_*F*_) is the transmission coefficient, calculated at the Fermi energy *E*_*F*_ of the electrodes. First, the ground-state Hamiltonian and optimized geometry of each compound were obtained using SIESTA.^39,40^ The van der Waals exchange-correlation functional was used along with double-ζ-polarized (DZP) basis sets and the norm-conserving pseudopotentials. The real space grid was defined by a plane wave cutoff of 250 Ry,^41,42^ and the geometry optimization was performed to a force tolerance of 0.01 eV/Å. This process was repeated for a unit cell with the molecule placed between two electrodes using the optimized distance between electrodes and the anchor groups shown in Table S2. The electrical properties of the molecular geometries were modeled using a combination of density functional theory (DFT) and quantum transport theory.^43,44^

Regarding the HC feature, this is common to all molecules independent of the tethering group, so one contact occurs via the thiol and the second contact must occur via the carbazole group directly, consistent with the proposed SAM geometry. The geometry of the HC junction was determined by examining the nature of the electrode contact with the carbazole and the conformation of the molecules in the junction. For the contact with the second electrode, we focus on **2a** due to its simplicity to examine contact either via the terminating hydrogens or through the π-system in a cofacial binding geometry or the carbazole (see SI, section S6.2). Enthalpically, the value of this interaction is too small to make a definitive conclusion; therefore, the transmission coefficients of each possible geometry were computed using the GOLLUM^45^ quantum transport code (see SI, section S6.3). Using the value of E – E_F_^DFT^ = 0.5 eV to afford the best fit of all data, the hydrogen contacted junctions (iii) and (iv) give a value of −4.3 log­(*G*/*G*_0_), while the cofacial contacted junction (i) gives a value of −2.6 log­(G/G_0_) (see SI, Figure S67); thus, the cofacial contact provides the closer fit to experimental conductance values of −3.0 log­(*G*/*G*_0_). Regarding molecular conformation, molecules **1a**, **1b**, **3a**, and **3b** can exist with each of their carbazoles stacking via an intramolecular π–π interaction (“closed-asym” conformation, see Figure 6(i) and (ii)) or with no interactions between the carbazoles in a splayed-out fashion (the “open-asym” conformation, see Figure 6(iii)). Both scenarios were modeled for each molecule using the van der Waals functional revealing that the closed conformation was enthalpically favorable by as much as 1.2 eV for **1b** (see SI, section S6.1). This contrasts with the behavior observed for the SAM, but can be explained by the isolation of the molecule in a molecular junction as compared to the assembly of molecules in a SAM; as a result, intramolecular π–π stacking dominates in the junction in the absence of competing intermolecular interactions. This is further supported by the calculated transmission coefficients with the closed geometries using the value of *E* – *E*_*F*_^DFT^ = 0.5 eV. The open-asym conformation (iii) **1b** gives a conductance of −3.5 log­(*G*/*G*_0_) while the closed-asym (i) and (ii) conformations give a conductance of −2.3 log­(*G*/*G*_0_) (see SI, Table S3). Taken together, these results indicate that the HC feature is attributed to a cofacial contact with the carbazole, and this must occur with molecules in a closed-asym conformation for the bicarbazole molecules.

The LC feature is more complex as **2b** does not show it, and it is associated with the stick–slip behavior of molecules **1a**, **1b**, and **2a**. Based on the breakoff distances of the LC feature, it is assumed that the bicarbazole molecules contact each electrode via a thiol contact. As in the previous case, all possible conformations of the molecules in the junction were considered, either where the carbazole groups stack via an intramolecular interaction (closed-asym, see Figure 6(iv)) or with no interactions between the carbazoles in a splayed-out fashion (the open-asym conformation, see Figure 6(v)). Each scenario was modeled using a van der Waals functional, and the closed conformation was enthalpically favorable by as much as 0.6 eV for **1b**. Transmission coefficients for the possible junction geometries were calculated using the value of *E* – *E*_*F*_^DFT^ = 0.5 eV; the open-asym (v) transmission was calculated to be −3.5 log­(*G*/*G*_0_) for **1b** while the closed-asym (iv) was calculated to be −2.9 log­(*G*/*G*_0_) showing an increase in conductance due to the addition of a π–π interaction between the carbazole groups.^13^ For molecules **3a** and **3b**, the difference between each conformation was greatly reduced due to the increased distance, 4 and 4.5 Å respectively, between the carbazole groups relative to **1a** and **1b**, ca. 3 Å. It is noteworthy that the difference between the closed-asym and open-asym is comparable to the difference for the stick–slip behavior observed for **1a** and **1b**, suggesting that as the junction extends, the majority of the molecules adopt the enthalpically favorable closed-asym conformation, but for 24.5% (**1a**) or 10% (**1b**) of the junctions, they formed initially adopt the open-asym conformation, but then switch to the closed-asym conformation resulting in the stick–slip behavior. For **3a** and **3b**, either fewer junctions are formed in the open-asym conformation or the difference in conductance between the conformations is so small that such stick–slip events cannot be readily observed during measurements. However, this cannot explain the stick–slip behavior of **2a** and the absence of any such behavior for **2b**. From the experimental (distance and conductance) data, the stick–slip events for **2a** have a different origin from that of **1a** and **1b**. The most significant structural difference between **2a** and **2b** and the other molecules is that **2a** and **2b** do not have a tethering group that covalently links the carbazole groups; therefore, they must form noncovalently π-stacked dimers, a common occurrence for planar aromatic molecular wires,^46^ to form a junction analogous to **1a** and **1b**. In the absence of a linking group, as the electrode distance is increased, the molecules forming the dimer slide past each other. When the transition coefficient of the dimer junctions is calculated as a function of distance, we observe two distinct differences between **2a** and **2b**. At the geometrically optimized starting position, **2b** has an almost 1 order of magnitude lower conductance than **2a,** and as the electrode distance increases, **2b** has a near exponential reduction in conductance while **2a** displays significant DQI features at *X* = 7.5 and 9.5 Å (*X* = 0.75 and 0.95 nm) (see Figure 7). Such DQI features observed by Frisenda et al.^11^ and explained by Al-Khaykanee et al.^47^ may account for the observed stick–slip behavior of **2a**, and due to the lateral displacement between the carbazole groups required for this to occur, it is not in conflict with the proposed explanation for the stick–slip behavior of **1a** and **1b** as the presence of the tethering groups would prevent such movement.

**Figure 7:**
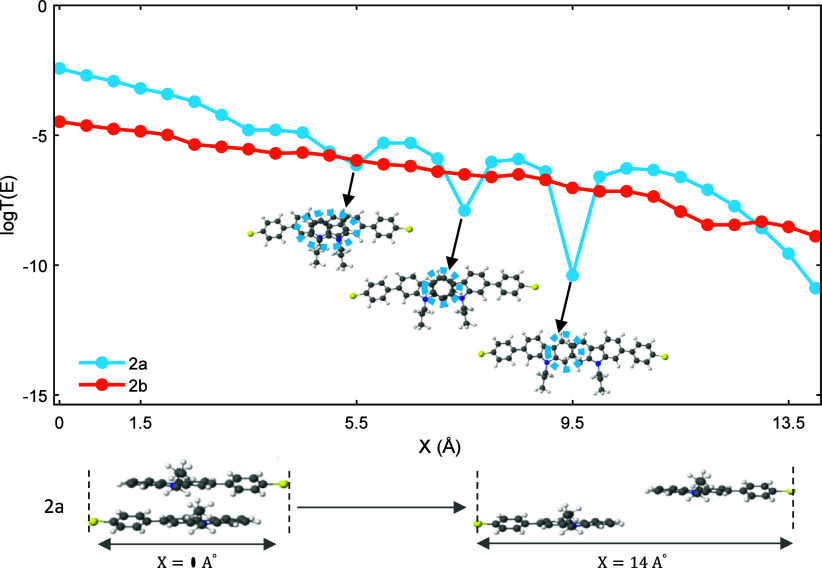
Transmission coefficient versus sliding positions between the carbazole units in dimers **2a** and **2b**. *T*(*E*) values are taken at *E* – *E*_*F*_^DFT^ = 0.5 eV.

## Conclusions

In this study, we have shown how structural flexibility, torsional degrees of freedom, and the number of thiol anchor groups combine to determine the electrical conductance of carbazole derivatives. For example, the monothiol **2a** and the flexibly tethered bicarbazoles with two thiols are predicted and observed to exhibit a slip–stick behavior, whereas the monothiol **2b** does not. Furthermore, **1a**, **1b**, **3a**, and **3b** can adopt conformations with each of their carbazoles stacking via an intramolecular π–π interaction, which in turn controls their electrical conductance. In total, six new carbazole-based molecular wires were synthesized, with both varied linking groups to tether the carbazole groups together and thiol groups attached at either the 2 or 3 position of the carbazole group(s). Through a combination of XPS, QCM, single-molecule conductance measurements, and DFT calculations, it was possible to follow the evolution of the molecular junctions from molecular deposition as a SAM to the complete retraction of the gold tip and examine how intra/intermolecular π–π interactions impact the molecular conductance as each molecule switched between different conformations. The importance of conductance via noncovalent π–π interactions in molecular junctions of carbazole/bicarbazole derivatives has been demonstrated. This work provides valuable insights for future experimental and theoretical studies on the interplay of conformation and conductance in highly flexible molecules that are anchored in molecular junctions.

## Supplementary Material



## Notes

Experimental procedures, NMR spectra, conductance measurements, XPS data and theoretical data, crystallographic data for compounds **1a-TMS**, **3b-TMS**, and **2a** (CCDC numbers 2388669–2388670) (PDF)
