# Supplementary material for: Single-Molecule and π–π-Stacked Dimer Electron Transport in Carbazole and Folded Bicarbazole Derivatives in Molecular Junctions
Source: ACS Omega. 2025 Oct 27;10(44):53540–8. doi: 10.1021/acsomega.5c08254 (PMC12612922; doi:10.1021/acsomega.5c08254)
Supplement: Supplementary file 1 [file ao5c08254_si_001.pdf]

SUPPORTING INFORMATION

Single-molecule and π-π-stacked dimer electron transport in carbazole and folded bicarbazole derivatives in molecular junctions

Adel Amer Alrehaili,[a,b†] Ross J. Davidson,[c†] Juan Hurtado-Gallego,[d†] Asma Alajmi,[a,e] Noorah Alwhaibi,[a] Andrei S. Batsanov,[c] Santiago Martin,[f,g,h] Pilar Cea,[f,g,h] Martin R. Bryce,[b*] Nicolás Agraït,[d*] Ali K. Ismael[a*,i] and Colin J. Lambert [a*]

a) *Department of Physics, University of Lancaster, Lancaster LA1 4YB, U.K.*

b) *Physics Department, Faculty of Science, Islamic University of Madinah, Madinah, 42351, Saudi Arabia.*

c) *Department of Chemistry, Durham University, Durham, DH1 3LE, U.K.*

d) *Departamento de Física de la Materia Condensada C-III, and Instituto Universitario de Ciencia de Materiales "Nicolás Cabrera", Universidad Autónoma de Madrid, E-28049 Madrid, Spain.*

e) *Department of Physics, College of Science and Humanities in Al-Kharj, Prince Sattam Bin Abdulaziz University, Al-Kharj 11942, Saudi Arabia.*

f) *Instituto de Nanociencia y Materiales de Aragón (INMA), CSIC-Universidad de Zaragoza, 50009, Zaragoza, Spain.*

g) *Departamento de Química Física, Facultad de Ciencias, Universidad de Zaragoza, 50009, Zaragoza, Spain.*

h) *Laboratorio de Microscopias Avanzadas (LMA), Universidad de Zaragoza, 50018, Zaragoza, Spain.*

i) *Department of Physics, College of Education for Pure Science, Tikrit University, Tikrit, Iraq.*

† These authors contributed equally to this work.

a) Experimental

S1. Synthesis S3

S2. NMR spectra of reported compounds S21

S3. Crystallographic data S39

S4. Conductance behavior S46

S4.1. Conductance measurements S46

S4.2. Conductance fluctuation analysis S51

S5. XPS measurements S55

b) Theoretical

S6. Density Functional Theory (DFT) Calculations S58

S6.1. Molecules in the gas phase S58

S6.2. Binding energies to gold electrodes S62

S6.3. Metal|Molecule|Metal junctions S65

S7. Comparison between experimental and theory S72

S8. π-π stacking S76

S9. Molecular design S78

References S80

a) Experimental

S1. Synthesis

Two new thioacetate-substituted monocarbazoles and four new thioacetate bi(carbazoles) were produced by two different approaches: approach A involved Suzuki–Miyaura coupling between (4-((2-(trimethylsilyl)ethyl)thio)phenyl)boronic acid and the respective halogenated carbazole followed by *N*-alkylation of the substituted carbazole by 1-iodopropane, 1,3-dibromopropane or 1,3-bis(bromomethyl)-5-(*tert*-butyl)benzene to give the ethyl-trimethylsilyl-protected intermediate. Approach B reversed the order, starting with *N*-alkylation of the respective halogenated carbazole followed by Suzuki–Miyaura coupling with (4-((2-(trimethylsilyl)ethyl)thio)phenyl)boronic acid to give the same ethyl-trimethylsilyl protected intermediate. There was little difference in the overall yields of the monocarbazoles (**2a-TMS** and **2b-TMS**) for either approach; however, **1a-TMS** and **1b-TMS** could only be synthesized by approach A while **3b-TMS** could only be isolated by approach B. These differences are attributed to the ease of purification. Finally, the ethyl-trimethylsilyl protected intermediates were deprotected by TBAF/acetyl chloride to give the final products **1**–**3a** and **1**–**3b**.

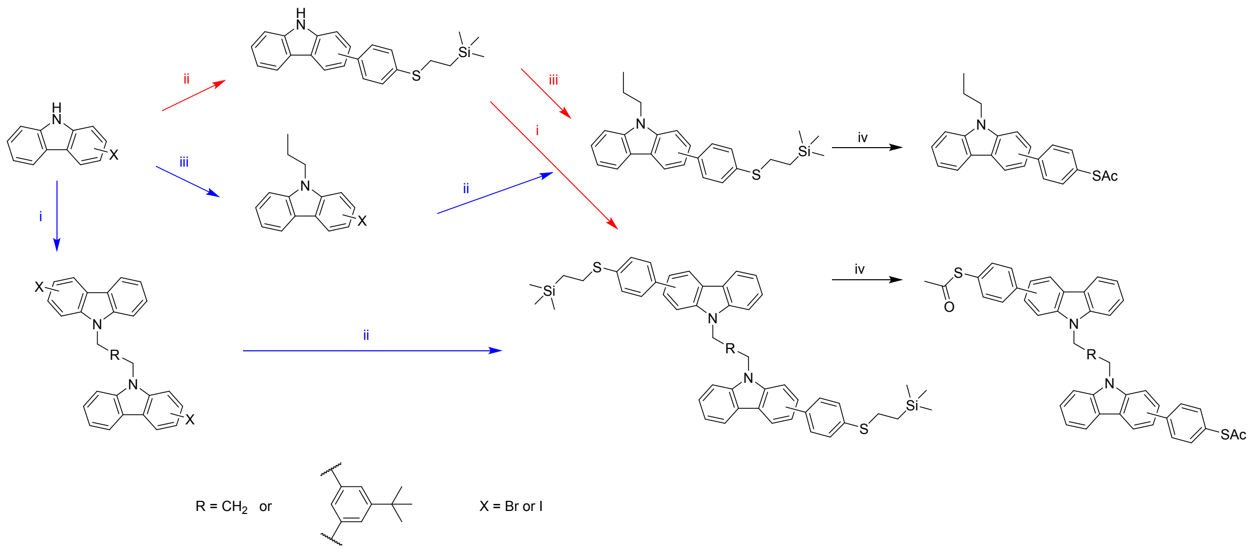

Scheme 1. Synthesis of monocarbazoles and folded bicarbazoles: Method A (blue): i or iii, then ii and iv; Method B (red): ii, then i or iii and iv, where i) 1,3-dibromopropane or 1,3-bis(bromomethyl)-5-(*tert*-butyl)benzene, K_2CO_3, DMF; ii) (4-((2-(trimethylsilyl)ethyl)thio)phenyl)boronic acid, K_2CO_3, $Pd(PPh_3)_4$, toluene, H_2O, EtOH; iii) 1-iodopropane, K_2CO_3, DMF; iv) TBAF, AcCl, THF.

Instrumentation. NMR spectra were recorded in deuterated solvent solutions using a Varian VNMRS-700 spectrometer and referenced against solvent resonances (^{1}H, ^{13}C). ASAP data were recorded using a Xevo QTOF (Waters) high-resolution accurate mass tandem mass spectrometer equipped with atmospheric pressure gas chromatography (APGC) and an atmospheric solids analysis probe (ASAP). Microanalyses were performed by the Elemental Microanalysis Service, Durham University, UK.

General details. The compounds 3-iodo-9-propyl-9*H*-carbazole[1], 2-bromo-9-propyl-9*H*-carbazole[1], 1,3-bis(bromomethyl)-5-(*tert*-butyl)benzene[2], and (4-((2-

(trimethylsilyl)ethyl)thio)phenyl)boronic acid[3] were prepared according to published methods. All other chemicals were sourced from standard chemical suppliers.

General synthesis of trimethyl(2-(phenylthio)ethyl)silane-substituted carbazoles. $Pd(PPh_3)_4$ (5 mol%) was added to a solution mixture containing 2- or 3-bromo-carbazole (4.00 g, 16.32 mmol), (4-((2-(trimethylsilyl)ethyl)thio)phenyl)boronic acid (4.56 g, 17.95 mmol), K_2CO_3 (2.47 g, 17.95 mmol), toluene (70 mL), water (5 mL) and EtOH (10 mL), which was then degassed by three freeze–pump–thaw cycles. The reaction mixture was heated to reflux for 16 hours before the solvent was removed in *vacuo*. The residue was then dissolved in DCM and filtered. The filtrate was collected and the solvent was removed, then initial purification was carried out by silica chromatography eluted by a solvent gradient from neat hexane to DCM:hexane (1:1).

2-(4-((2-(trimethylsilyl)ethyl)thio)phenyl)-carbazole. A white solid. **Yield**: 4.78 g (78%). **^{1}H NMR** (700 MHz; CD_2Cl_2): δ_H 8.26 (br, 1H, Hj), 8.13 (d, $^3J_{HH}$ = 8.1 Hz, 1H, H_c), 8.10 (d, $^3J_{HH}$ = 8.2 Hz, 1H, H_d), 7.66-7.65 (m, 3H, H_i+H_g), 7.50-7.48 (m, 2H, H_a+H_b), 7.45-7.40 (m, 3H, H_f+H_i), 7.26 (ddd, $^3J_{HH}$ = 8.0 Hz, $^3J_{HH}$ = 6.9 Hz, $^4J_{HH}$ = 1.1 Hz, 1H, H_e), 3.06-3.00 (m, 2H, H_k), 1.01-0.96 (m, 2H, H_l), 0.08 (s, 9H, H_m) ppm. **^{13}C{^{1}H} NMR** (176 MHz; CD_2Cl_2): δ_C 140.1, 140.0, 138.9, 138.3, 136.4, 128.9, 127.6, 125.8, 122.9, 122.4, 120.4, 120.2, 119.5, 118.6, 110.6, 108.6, 29.3, 16.8, -2.1 ppm. **Acc-MS**(ASAP$^+$): *m/z* 376.1564 $[M+H]^+$ calcd. for $C_{24}H_{26}NSSi$ *m/z* 376.1555 ($|\Delta m/z|$ = 2.4 ppm). **Anal. Calc.** for $C_{23}H_{25}NSSi$: C, 73.55; H, 6.71; N, 3.73 %. **Found**: C, 73.31; H, 6.62; N, 3.66 %.

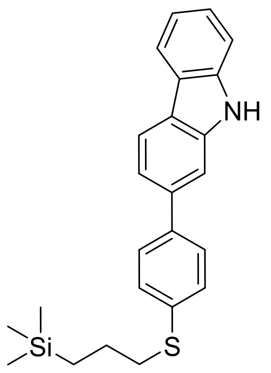

3-(4-((2-(trimethylsilyl)ethyl)thio)phenyl)-carbazole. A white solid. **Yield**: 5.39 g (80%). **^{1}H NMR** (700 MHz; CD_2Cl_2): δ_H 8.32(s, 1H, H_a), 8.25 (br, 1H, H_j), 8.15 (dd, $^3J_{HH}$ = 7.8 Hz, $^4J_{HH}$ = 1.0 Hz, 1H, H_g), 7.70-7.67 (m, 3H, H_b+H_h), 7.53 (d, $^3J_{HH}$ = 8.3 Hz, 1H, H_c), 7.50-7.42 (m, 4H, H_d+H_e+H_i), 7.27 (ddd, $^3J_{HH}$ = 8.0 Hz, $^3J_{HH}$ = 6.9 Hz, $^4J_{HH}$ = 1.2 Hz, 1H, H_f), 3.07-3.04 (m, 2H, H_k), 1.02-0.99 (m, 2H, H_l), 0.09 (s, 9H, H_m) ppm. **^{13}C{^{1}H} NMR** (176 MHz; CD_2Cl_2): δ_C 140.0, 139.3, 139.0, 135.4, 132.0, 129.2, 127.4, 126.0, 125.0, 123.7, 123.2, 120.2, 119.5, 118.3, 110.9, 110.8, 29.5, 16.8, -2.1 ppm. **Acc-MS**(ASAP$^+$): *m/z* 376.1540 [M+H]$^+$ calcd. for $C_{23}H_{26}NSSi$ *m/z* 376.1555 (|Δ*m/z*| = 4.0 ppm). **Anal. Calc.** for $C_{23}H_{25}NSSi$: C, 73.55; H, 6.71; N, 3.73 %. **Found**: C, 73.66; H, 6.62; N, 3.74 %.

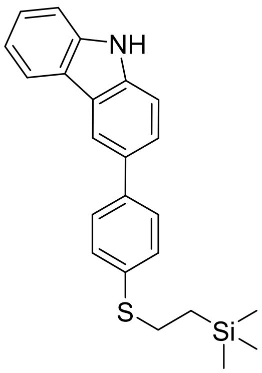

General synthesis of bis(halocarbazole) alkyls. Halocarbazole (3-iodocarbazole, 3-bromocarbazole or 2-bromocarbazole) (13.65 mmol, 2 eq) was added to a suspension of NaH (327 mg, 13.65 mmol, 2 eq) in DMF (30 mL), immediately forming a yellow solution. After 1 h stirring at room temperature, the dihaloalkane (1,3-dibromopropane or 1,3-bis(bromomethyl)-5-(tert-butyl)benzene) (6.82 mmol, 1 eq) was added and stirring was continued at room temperature for 16 hours. Water was added, forming a white precipitate that was collected by filtration and washed thoroughly by MeOH. The precipitate was recrystallized in $CHCl_3$, with the crystals collected by filtration.

1,3-bis(3-iodo-carbazol-9-yl)propane. A white solid. **Yield**: 1.6 g (18%).**^{1}H NMR** (700 MHz; $CDCl_3$): δ_H 8.39 (d, J^4_{HH} = 1.7 Hz, 2H, H_e), 8.02 (d, J^3_{HH} = 7.7 Hz, 2H, H_d), 7.62 (dd, J^3_{HH} = 8.5 Hz, J^4_{HH} = 1.7 Hz,2H, H_f), 7.40 (ddd, J^3_{HH} = 8.2 Hz, J^3_{HH} = 7.1 Hz, J^4_{HH} = 1.2 Hz, 2H, H_b), 7.23 (ddd, J^3_{HH} = 8.2 Hz, J^3_{HH} = 7.1 Hz, J^4_{HH} = 1.2 Hz, 2H, H_c), 7.15 (d, J^3_{HH} = 8.2 Hz, 2H, H_a), 6.92 (d, J^3_{HH} = 8.5 Hz, 2H, H_g), 4.28 (t, J^3_{HH} = 7.3 Hz, 4H, H_h), 2.42 (p, J^3_{HH} = 7.3 Hz, 2H, H_i) ppm. **^{13}C{^{1}H} NMR** (176 MHz; $CDCl_3$): δ_C 140.0, 139.2, 134.0, 129.3, 126.5, 121.7, 120.6, 119.7, 110.3, 108.5, 81.6, 40.4, 27.6 ppm. **Acc-MS**(ASAP$^+$): *m/z* 625.9724 $[M]^+$ calcd. for $C_{27}H_{20}I_2N_2$ *m/z* 625.9716 ($|\Delta m/z|$ = 1.3 ppm). **Anal. Calc.** for $C_{27}H_{20}I_2N_2$: C, 51.75; H, 3.18; N, 4.52 %. **Found**: C, 51.78; H, 3.22; N, 4.47 %.

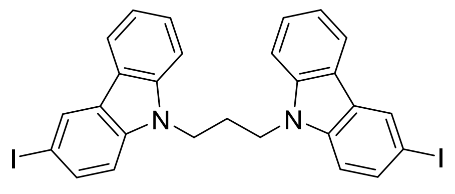

1,3-bis(2-bromo-9H-carbazol-9-yl)propane. A white solid. **Yield**: 1.12 g (31%).**^{1}H NMR** (700 MHz; CD_2Cl_2): δ_H 8.09 (dt, $^3J_{HH}$ = 7.7 Hz, $^4J_{HH}$ = 0.9 Hz, 2H, H_d), 7.98 (d, $^3J_{HH}$ = 8.2 Hz, 2H, H_c), 7.43 (ddd, $^3J_{HH}$ = 8.3 Hz, $^3J_{HH}$ = 7.2 Hz, $^4J_{HH}$ = 1.2 Hz, 2H, H_f), 7.39 (d, $^4J_{HH}$ = 1.7 Hz, 2H, H_a), 7.34 (dd, $^3J_{HH}$ = 8.2 Hz, $^4J_{HH}$ = 1.7 Hz, 2H, H_b), 7.25 (td, $^3J_{HH}$ = 7.5 Hz, $^4J_{HH}$ = 0.9 Hz, 2H, H_e), 7.22 (d, $^3J_{HH}$ = 8.3 Hz, 2H, H_g), 4.33 (t, $^3J_{HH}$ = 7.4 Hz, 4H, H_h), 2.46 (p, $^3J_{HH}$ = 7.3 Hz, 2H, H_i) ppm. **^{13}C{^{1}H} NMR** (176 MHz; $CDCl_3$): δ_C 140.9, 140.2, 126.2, 122.3, 122.1, 121.9, 121.5, 120.4, 119.6, 119.2, 40.52, 27.59 ppm. **Acc-MS**(ASAP$^+$): *m/z* 530.0015 [M]$^+$ calcd. for $C_{27}H_{20}I_2N_2$ *m/z* 529.9993 (|Δ*m/z*| = 4.2 ppm). **Anal. Calc.** for $C_{27}H_{20}Br_2N_2$: C, 60.93; H, 3.79; N, 5.26 %. **Found**: C, 60.74; H, 3.73; N, 5.19 %.

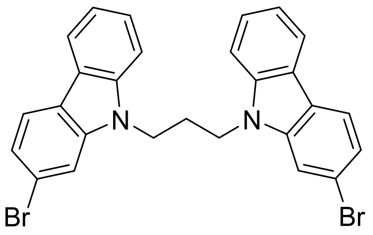

9,9'-((5-(tert-butyl)-1,3-phenylene)bis(methylene))bis(2-bromo-carbazole). A white solid. **Yield**: 1.55 g (43%).**^{1}H NMR** (500 MHz; CD_2Cl_2): δ_H 8.07 (dd, J^3_{HH} = 7.7 Hz, J = 1.2 Hz, 2H, H_d) 7.97 (d, J^3_{HH} = 8.2 Hz, 2H, H_c), 7.41-7.34 (m, 6H, H_a+H_b+H_f), 7.28-7.18 (m, 6H, H_e+H_g+H_i), 6.33 (s, 1H, H_h), 5.35 (m, 4H, H_j), 1.20 (s, 9H, H_k) ppm. **^{13}C{^{1}H} NMR** (125 MHz; CD_2Cl_2): δ_C 152.5, 141.2, 140.6, 137.1, 126.2, 122.8, 122.2, 121.8, 121.5, 121.1, 120.3, 119.6, 119.1, 111.7, 108.9, 46.5, 34.4, 30.8 ppm. **Acc-MS**(ASAP$^+$): *m/z* 649.0825 [M+H]$^+$ calcd. for $C_{36}H_{31}Br_2N_2$ *m/z* 649.0854 (|Δ*m/z*| = 4.8 ppm). **Anal. Calc.** for $C_{36}H_{30}Br_2N_2$: C, 66.48; H, 4.65; N, 4.31 %. **Found**: C, 66.62; H, 4.44; N, 4.26 %.

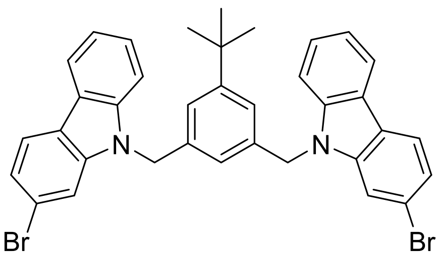

9,9'-((5-(tert-butyl)-1,3-phenylene)bis(methylene))bis(3-bromo-carbazole). A white solid. **Yield**: 1.40 g (39%).**^{1}H NMR** (700 MHz; CD_2Cl_2): δ_H 8.18 (d, $^{4}J_{HH}$ = 1.9 Hz, 2H, H_c), 8.03 (d, $^{3}J_{HH}$ = 8.2 Hz, 2H, H_d), 7.35-7.32 (m, 4H, H_b+H_f), 7.24 (t, $^{3}J_{HH}$ = 7.7 Hz, 2H, H_e), 7.18 (d, $^{4}J_{HH}$ = 1.8 Hz, 2H, H_i), 7.16 (d, $^{3}J_{HH}$ = 8.2 Hz, 2H, H_a), 6.98 (d, $^{3}J_{HH}$ = 8.7 Hz, 2H, H_g), 6.08 (s, 1H, H_h), 5.30 (s, 4H, H_i), 1.20 (s, 9H, H_k) ppm. **^{13}C{^{1}H} NMR** (176 MHz; CD_2Cl_2): δ_C 152.5, 140.6, 139.0, 137.1, 128.1, 126.3, 124.4, 123.0, 122.8, 121.7, 120.9, 120.4, 119.4, 111.6, 110.0, 108.8, 46.3, 34.4, 30.8 ppm. **Acc-MS**(ASAP^{+}): *m/z* 649.0865 [M+H]$^{+}$ calcd. for $C_{36}H_{31}N_2Br_2$ *m/z* 649.0854 (|Δ*m/z*| = 1.7 ppm). **Anal. Calc.** for $C_{36}H_{30}Br_2N_2$: C, 66.48; H, 4.65; N, 4.31 %. **Found**: C, 66.03; H, 4.65; N, 4.20 %.

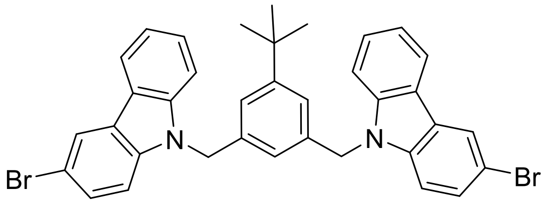

Propane carbazole phenyl compounds: route A. $Pd(PPh_3)_4$ (5 mmol%) was added to a solution mixture containing the halo-carbazole compound (3-iodo-9-propyl-carbazole, 2-bromo-9-propyl-carbazole, 1,3-bis(3-iodo-carbazol-9-yl)propane, 1,3-bis(2-bromo-9*H*-carbazol-9-yl)propane, 9,9'-((5-(*tert*-butyl)-1,3-phenylene)bis(methylene))bis(2-bromo-carbazole) or 9,9'-((5-(*tert*-butyl)-1,3-phenylene)bis(methylene))bis(3-bromo-carbazole)) (1 eq), (4-((2-(trimethylsilyl)ethyl)thio)phenyl)boronic acid (mono-carbazole (1.1 eq) or bis-carbazole (2.2

eq)), K_2CO_3 (mono-carbazole (1.1 eq) or bi-carbazole (2.2 eq)), toluene (70 mL) and EtOH (10 mL), then degassed by three freeze–pump–thaw cycles. The reaction mixture was heated to reflux for 16 hours before the solvent was removed in *vacuo*, the residue was then dissolved in DCM and filtered. The filtrate was collected and the solvent removed, followed by purification using silica chromatography eluted by a solvent gradient from neat hexane to DCM:hexane (1:1).

Propane carbazole phenyl compounds: route B. Halo-alkane (1-iodopropane (1.1 eq)), 1(,3-dibromopropane (0.5 eq) or 1,3-bis(bromomethyl)-5-(*tert*-butyl)benzene (0.5 eq)) was added to a suspension containing 2-(4-((2-(trimethylsilyl)ethyl)thio)phenyl)-carbazole or 3-(4-((2-(trimethylsilyl)ethyl)thio)phenyl)-carbazole (1.0 eq) and NaH (1.1 eq) in DMF (30 mL). This suspension was stirred for 16 hours at room temperature, after which water was added, forming a turbid solution. This was extracted by DCM then the organic layer was washed twice with water. The organic layer was collected and dried over $MgSO_4$ and filtered. The filtrate was collected and purified by silica chromatography eluted by a solvent gradient from neat hexane to DCM:hexane (1:1).

1,3-bis(2-(4-((2-(trimethylsilyl)ethyl)thio)phenyl)-carbazol-9-yl)propane (**1a-TMS**). White solid. X-ray suitable crystals were grown by layering MeOH onto a solution of **1a-TMS** in DCM. **Yield**: method A: 0.90 g (61%), method B: pure product not isolated.**^{1}H NMR** (700 MHz; $CDCl_3$): δ_H 8.11-8.08 (m, 4H, H_c+H_d), 7.42 (m, 8H, H_b+H_f+H_h), 7.34 (d, $^3J_{HH}$ = 8.0 Hz, 4H, H_i), 7.27 (d, $^3J_{HH}$ = 8.1 Hz, 2H, H_g), 7.24-7.22 (m, 4H, H_a+H_e), 4.41 (t, $^3J_{HH}$ = 7.2 Hz, 4H, H_k) 3.04-3.02 (m, 4H, H_l), 2.55 (p, $^3J_{HH}$ = 7.3 Hz, 2H, H_j), 1.00-0.98 (m, 4H, H_m), 0.07 (s, 18H, H_n) ppm.

$^{13}C\{^1H\}$ NMR (176 MHz; $CDCl_3$): δ_C 140.6, 139.2, 138.5, 136.0, 129.2, 127.7, 125.8, 122.8, 122.2, 120.7, 120.5, 119.3, 118.6, 108.4, 106.5 ppm. **Acc-MS**(ASAP$^+$): *m/z* 790.3250 $[M]^+$ calcd. for $C_{49}H_{54}N_2S_2Si_2$ *m/z* 790.3267 (|Δ*m/z*| = 2.2 ppm). **Anal. Calc.** for $C_{49}H_{54}N_2S_2Si_2$: C, 74.38; H, 6.88; N, 3.54 %. **Found**: C, 74.25; H, 6.65; N, 3.52 %.

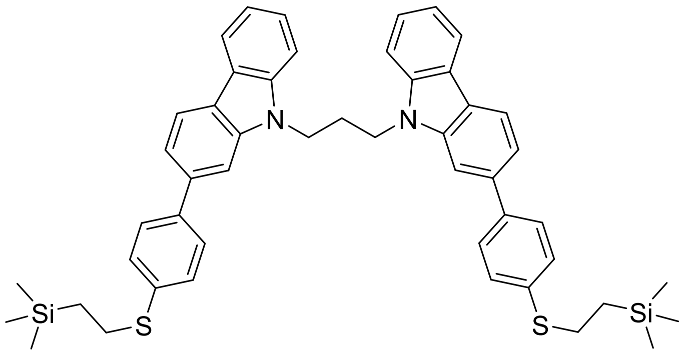

1,3-bis(3-(4-((2-(trimethylsilyl)ethyl)thio)phenyl)-carbazol-9-yl)propane (**1b-TMS**). Method A: this was further purified using normal phase HPLC eluted by solvent gradient from DCM:Hexane (1:9) to neat DCM, to yield a pale-yellow oil. **Yield**: method A: 0.74 g (50%), method B: pure product not isolated.**^1H NMR** (700 MHz; CD_2Cl_2): δ_H 8.33 (d, $^4J_{HH}$ = 1.8 Hz, 2H, H_e), 8.16 (dd, $^3J_{HH}$ = 7.8 Hz, $^4J_{HH}$ = 1.1 Hz, 2H, H_d), 7.66-7.64 (m, 6H, H_f+H_h), 7.44-7.40 (m, 6H, H_b+H_i), 7.30-7.24 (m, 6H, H_a+H_c+H_g), 4.41 (t, $^3J_{HH}$ = 7.4 Hz, 4H, H_m), 3.05-3.02 (m, 4H, H_j), 2.51 (p, $^3J_{HH}$ = 7.3 Hz, 2H, H_n), 1.00-0.98 (m, 4H, H_k), 0.08 (s, 18H, H_l) ppm. **$^{13}C\{^1H\}$ NMR** (176 MHz; CD_2Cl_2): δ_C 140.6, 139.6, 139.2, 135.4, 131.7, 129.2, 127.3, 126.0, 124.9, 123.4, 123.0, 120.4, 119.2, 118.4, 108.8, 108.6, 40.6, 29.5, 27.9, 16.8, -2.1 ppm. **Acc-MS**(ASAP$^+$): *m/z* 791.3375 $[M+H]^+$ calcd. for $C_{49}H_{55}N_2S_2Si_2$ *m/z* 791.3345 (|Δ*m/z*| = 3.8 ppm). **Anal. Calc.** for $C_{49}H_{54}N_2S_2Si_2$: C, 74.38; H, 6.88; N, 3.54 %. **Found**: C, 73.92; H, 6.67; N, 3.43 %.

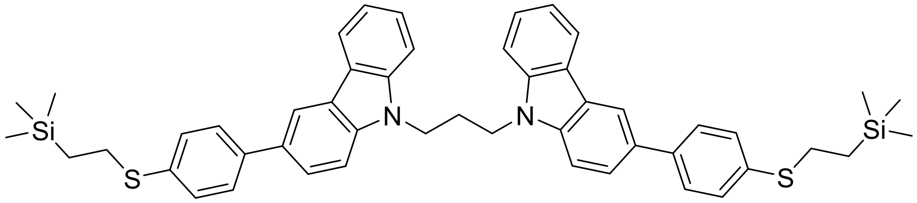

9-propyl-3-(4-((2-(trimethylsilyl)ethyl)thio)phenyl)-carbazole (**2a-TMS**). A white sold. **Yield**: Method A: 0.75 (61%), Method B: 0.90 g (73%). **^{1}H NMR** (500 MHz; CD_2Cl_2): δ_H 8.18 (d $^3J_{HH}$ = 8.1 Hz, 1H, H_c), 8.14 (d, $^3J_{HH}$ = 7.8 Hz, 1H, H_d), 7.72 (dd, $^3J_{HH}$ = 8.2 Hz, $^4J_{HH}$ = 1.3 Hz, 2H, H_h), 7.65 (s, 1H, H_a), 7.51-7.49 (m, 3H, H_b+H_f+H_g), 7.45 (d, $^3J_{HH}$ = 8.2 Hz, 2H, H_i), 7.29-7.26 (m, 1H, H_e), 4.38 (t, $^3J_{HH}$ = 7.2 Hz, 2H, H_j), 3.10-3.05 (m, 2H, H_m), 1.98 (h, $^3J_{HH}$ = 7.4 Hz, 2H, H_k), 1.05-0.99 (m, 5H, H_l+H_n), 0.11 (s, 9H, H_o) ppm. **^{13}C{^{1}H} NMR** (125 MHz; $CDCl_3$): δ_C 141.0, 139.3, 138.2, 136.3, 128.9, 127.8, 125.6, 122.4, 121.9, 120.5, 120.2, 118.8, 118.0, 108.8, 106.9, 44.5, 29.3, 22.3, 16.8, 11.5, -2.1 ppm. **Acc-MS**(ASAP$^+$): *m/z* 418.2033 $[M+H]^+$ calcd. for $C_{26}H_{32}NSSi$ *m/z* 418.2025 (|Δ*m/z*| = 1.9 ppm). **Anal. Calc.** for $C_{26}H_{31}NSSi$: C, 74.77; H, 7.48; N, 3.35 %. **Found**: C, 74.83; H, 7.65; N, 6.35 %.

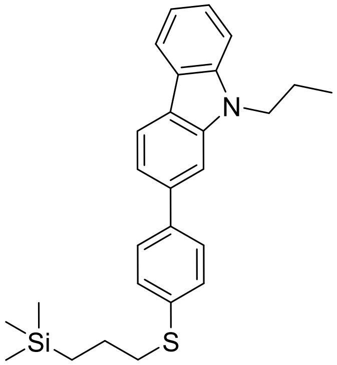

9-propyl-3-(4-((2-(trimethylsilyl)ethyl)thio)phenyl)-carbazole (**2b-TMS**). Using method A this was further purified using normal phase HPLC eluted by solvent gradient from DCM:Hexane (1:9) to neat DCM, to yield a pale-yellow oil. **Yield**: Method A: 0.19 g (16%), Method B: 0.80 g

(65%).**^{1}H NMR** (500 MHz; $CDCl_3$): δ_H 8.37 (t, $^4J_{HH}$ = 1.6 Hz, 1H, H_a), 8.20 (dd, $^3J_{HH}$ = 7.8 Hz, $^4J_{HH}$ = 1.4 Hz, 1H, H_d), 7.76 (dt, $^3J_{HH}$ = 8.5 Hz, $^4J_{HH}$ = 1.6 Hz, 1H, H_g), 7.71 (d, $^3J_{HH}$ = 8.0 Hz, 2H, H_h), 7.55-7.45 (m, 5H, H_b+H_c+H_f+H_i), 7.29 (tt, $^3J_{HH}$ = 6.3 Hz, $^4J_{HH}$ = 1.6 Hz, 1H, H_e), 4.35 (t, $^3J_{HH}$ = 7.2 Hz, 2H, H_j), 3.11-3.06 (m, 2H, H_m), 1.98 (h, $^3J_{HH}$ = 7.4 Hz, 2H, H_k), 1.06-1.00 (m, 5H, H_l+H_n), 0.12 (s, 9H, H_o) ppm. **^{13}C{^{1}H} NMR** (125 MHz; $CDCl_3$): δ_C 141.0, 140.0, 139.4, 135.3, 131.3, 129.2, 127.4, 125.8, 124.7, 123.2, 122.7, 120.3, 118.8, 118.3, 109.1, 109.0, 44.7, 29.5, 22.3, 16.9, 11.5, -2.0 ppm. **Acc-MS**(ASAP$^+$): *m/z* 417.1967 $[M]^+$ calcd. for $C_{26}H_{31}NSSi$ *m/z* 417.1947 (|Δ*m/z*| = 4.8 ppm). **Anal. Calc.** for $C_{26}H_{31}NSSi$: C, 74.77; H, 7.48; N, 3.35 %. **Found**: C, 74.63; H, 7.36; N, 3.43 %.

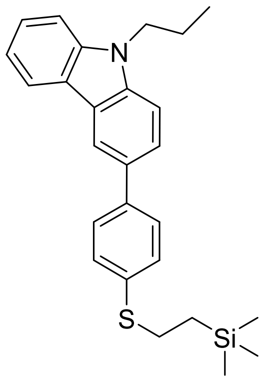

9,9'-((5-(tert-butyl)-1,3-phenylene)bis(methylene))bis(2-(4-((2-(trimethylsill)ethyl)thio)phenyl)-carbazole) (**3a-TMS**). A white solid. **Yield**: method A: 1.12 g (80%), method B: 0.80 g (67 %). **^{1}H NMR** (700 MHz; CD_2Cl_2): δ_H 8.08 (d, $^3J_{HH}$ = 8.4 Hz, 2H, H_c), 8.05 (d, $^3J_{HH}$ = 7.7 Hz, 2H, H_d), 7.50 (d, $^3J_{HH}$ = 7.50 Hz, 4H, H_h), 7.43-7.41 (m, 4H, H_a+H_b), 7.35 (d, $^3J_{HH}$ = 7.50 Hz, 4H, H_i), 7.29 (dd, $^3J_{HH}$ = 8.1 Hz, $^4J_{HH}$ = 7.7, 2H, H_f), 7.22-7.18 (m, 6H, H_e+H_g+H_k), 6.60 (d, $^4J_{HH}$ = 2.1 Hz, 1H, H_j), 5.39 (s, 4H, H_m), 3.06-3.03 (m, 4H, H_n), 1.18 (s, 9H, H_l), 1.02-0.99 (m, 4H, H_o), 0.09 (s, 18H, H_p) ppm. **^{13}C{^{1}H} NMR** (176 MHz; CD_2Cl_2): δ_C 1523, 141.1, 141.0, 139.0, 138.2,

137.6, 136.2, 128.9, 127.6, 125.7, 123.0, 122.6, 122.0, 121.6, 120.4, 120.2, 119.2, 118.3, 108.8, 106.7, 46.6, 34.4, 30.8, 29.3, 16.8, -2.1 ppm. **Acc-MS**(ASAP$^+$): *m/z* 909.4144 $[M+H]^+$ calcd. for $C_{58}H_{65}N_2S_2Si_2$ *m/z* 909.4128 ($|\Delta m/z|$ = 2.8 ppm). **Anal. Calc.** for $C_{58}H_{64}N_2S_2Si_2$: C, 76.60; H, 7.09; N, 3.08 %. **Found**: C, 76.54; H, 6.94; N, 3.00 %.

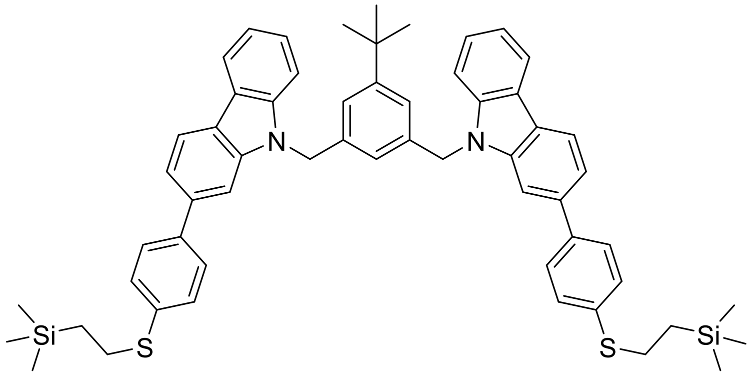

RJD1908 *9,9'-((5-(tert-butyl)-1,3-phenylene)bis(methylene))bis(3-(4-((2-(trimethylsilyl)ethyl)thio)phenyl)-9H-carbazole)* (**3b-TMS**). A white solid. X-ray suitable crystals were grown by layering MeOH onto a solution of **3b-TMS** in DCM. **Yield**: method A: pure product not isolated, method B: 0.66 g (56 %).**^{1}H NMR** (500 MHz; CD_2Cl_2): δ_H 8.31 (t, $^3J_{HH}$ = 1.3 Hz, 2H, H_a), 8.15 (d, $^3J_{HH}$ = 7.7 Hz, 2H, H_d), 7.67 (d, $^3J_{HH}$ = 8.3 Hz, 4H, H_h), 7.59 (dt, $^3J_{HH}$ = 8.3 Hz, $^4J_{HH}$ = 1.5 Hz, 2H, H_g), 7.46 (d, $^3J_{HH}$ = 8.3 Hz, 4H, H_i), 7.39 (ddd, $^3J_{HH}$ = 8.3 Hz, $^3J_{HH}$ = 7.0 Hz, $^4J_{HH}$ = 1.2 Hz, 2H, H_f), 7.30-7.25 (m, 8H, H_b+H_c+H_e+H_k), 6.55 (s, 1H, H_l), 5.40 (s, 4H, H_j), 3.13-3.08 (m, 4H, H_m), 1.22 (s, 9H, H_f), 1.07-1.03 (m, 4H, H_n), 0.13 (s, 18H, H_o) ppm. **^{13}C{^{1}H} NMR** (125 MHz; CD_2Cl_3): δ_C 152.4, 140.9, 139.9, 139.2, 137.5, 135.4, 131.5, 129.2, 127.3, 125.9, 124.7, 123.3, 123.0, 122.9, 121.8, 120.3, 119.2, 118.3, 109.0, 108.9, 46.6, 34.4, 30.9, 29.5, 16.8, -2.0 ppm. **Acc-MS**(ASAP$^+$): *m/z* 909.4150 $[M+H]^+$ calcd. for

$C_{58}H_{65}N_2S_2Si_2$ *m/z* 909.4128 (|Δ*m/z*| = 2.4 ppm). **Anal. Calc.** $C_{58}H_{64}N_2S_2Si_2$: C, 76.60; H, 7.09; N, 3.08 %. **Found**: C, 76.49; H, 6.97; N, 2.96 %.

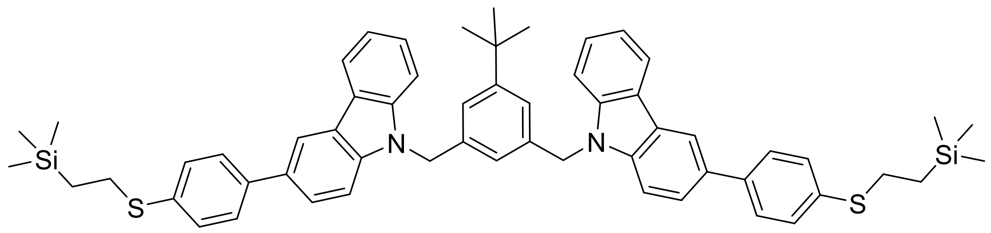

Ethyl-TMS deprotection: general method. The ethyl-TMS protected compound was dissolved in dry THF, then tetrabutylammonium fluoride solution (1.0 M) was added (3 eq per TMS group) and stirring was continued for 2 hours before acetyl chloride (3 eq per TMS group) was added. Stirring was continued for a further 16 hours before the solution was poured into water and extracted three times with DCM. The organic layers were combined and dried over $MgSO_4$. The solution was filtered and the solvent was removed from the filtrate in *vacuo*. Purification was achieved by silica chromatography eluted by a solvent gradient from neat hexane to neat DCM for the mono-thioacetate compounds and a solvent gradient of DCM:hexane 1:1 to DCM:ethylacetate 9:1 for the di-thioacetate compounds.

S,S'-((propane-1,3-diylbis(carbazole-9,2-diyl))bis(4,1-phenylene)) diethanethioate (**1a**). A white solid. **Yield**: 94 mg (35 %). **^{1}H NMR** (500 MHz; CD_2Cl_2): δ_H 8.19 (d, $^3J_{HH}$ = 8.1 Hz, 2H, H_c), 8.16 (d, $^3J_{HH}$ = 7.7 Hz, 2H, H_d), 7.58 (d, $^3J_{HH}$ = 8.0 Hz, 4H, H_h), 7.51-7.45 (m, 8H, H_b+H_f+H_i), 7.38 (d, $^3J_{HH}$ = 8.2 Hz, 2H, H_g), 7.34 (s, 2H, H_a), 7.28 (t, $^3J_{HH}$ = 7.5 Hz, 2H, H_e), 4.48 (t, $^3J_{HH}$ = 7.3 Hz, 4H, H_k), 2.58 (p, $^3J_{HH}$ = 7.4 Hz, 2H, H_l), 2.49 (s, 6H, H_j) ppm. **^{13}C{^{1}H} NMR** (125 MHz;

CD_2Cl_2): δ_C 194.1, 142.9, 140.7, 140.6, 137.9, 134.8, 128.0, 126.7, 126.0, 122.5, 120.7, 120.5, 119.3, 118.7, 108.6, 107.0, 40.3, 30.1, 27.8 ppm. **Acc-MS**(ASAP$^+$): *m/z* 675.2167 $[M+H]^+$ calcd. for $C_{43}H_{35}N_2S_2O_2$ *m/z* 675.2140 ($|\Delta m/z|$ = 4.0 ppm). **Anal. Calc.** for $C_{43}H_{34}N_2O_2S_2$: C, 76.53; H, 5.08; N, 4.15 %. **Found**: C, 76.42; H, 4.77; N, 3.91 %.

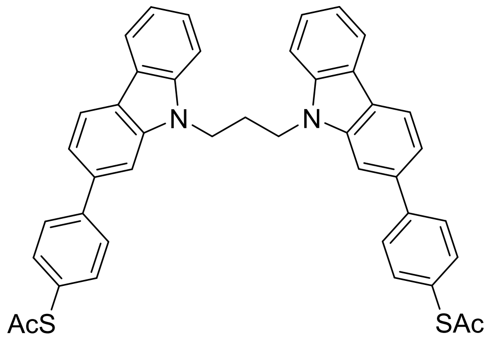

S,S'-((propane-1,3-diylbis(9H-carbazole-9,3-diyl))bis(4,1-phenylene)) diethanethioate (**1b**). A white solid. **Yield**: 75 mg (28%). **^{1}H NMR** (500 MHz; CD_2Cl_2): δ_H 8.39 (d, $^4J_{HH}$ = 1.7 Hz, 1H, H_c), 8.21 (d, $^3J_{HH}$ = 7.7 Hz, 2H, H_d), 7.80 (d, $^3J_{HH}$ = 8.0 Hz, 4H, H_h), 7.72 (dt, $^3J_{HH}$ = 8.5 Hz, $^4J_{HH}$ = 1.5 Hz, 2H, H_b), 7.54 (d, $^3J_{HH}$ = 8.0 Hz, 4H, H_i), 7.74 (dd, $^3J_{HH}$ = 8.3 Hz, $^3J_{HH}$ = 7.0 Hz, 2H, H_f), 7.36-7.28 (6H, H_a+H_e+H_g), 4.46 (t, $^3J_{HH}$ = 7.3 Hz, 4H, H_k), 2.56 (p, $^3J_{HH}$ = 7.4 Hz, 2H, H_l), 2.48 (s, 6H, H_j) ppm. **^{13}C{^{1}H} NMR** (125 MHz; CD_2Cl_2): δ_C 194.3, 143.0, 140.6, 139.9, 135.0, 131.2, 127.7, 126.1, 125.9, 125.1, 123.5, 122.9, 120.5, 119.3, 118.9, 108.9, 108.7, 40.6, 30.0, 27.9 ppm. **Acc-MS**(ASAP$^+$): *m/z* 675.2163 $[M+H]^+$ calcd. for $C_{43}H_{35}N_2$ O_2S_2 *m/z* 675.2140 ($|\Delta m/z|$ = 3.6 ppm). **Anal. Calc.** for $C_{43}H_{34}N_2O_2S_2$: C, 76.53; H, 5.08; N, 4.15 %. **Found**: C, 76.32; H, 4.72; N, 3.97 %.

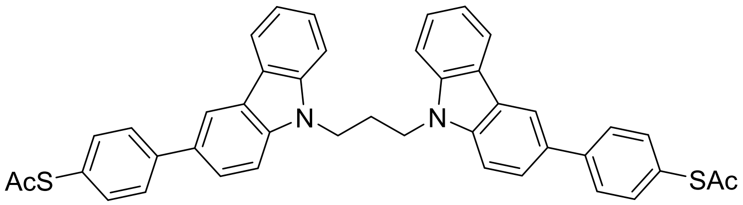

S-(4-(9-propyl-carbazol-2-yl)phenyl) ethanethioate (**2a**). A white solid. X-ray suitable crystals were grown by slow evaporation of a DCM/*n*-Hexane solution. **Yield**: 0.23 g (54%). **^{1}H NMR** (500 MHz; CD_2Cl_2): δ_H 8.17 (d, $^3J_{HH}$ = 8.0 Hz, 1H, H_c), 8.13 (dt, $^3J_{HH}$ = 7.8 Hz, $^4J_{HH}$ = 1.0 Hz, 1H, H_d), 7.81-7.79 (m, 2H, H_h), 7.67 (d, $^4J_{HH}$ = 1.5 Hz, 1H, H_a), 7.55-7.52 (m, 2H, H_i), 7.51-7.46 (m, 3H, H_b+H_f+H_g), 7.25 (ddd, $^3J_{HH}$ = 7.9 Hz, $^3J_{HH}$ = 6.5 Hz, $^4J_{HH}$ = 1.6 Hz, 1H, H_e), 4.35 (t, $^3J_{HH}$ = 7.2 Hz, 2H, H_j), 2.45 (s, 3H, H_m), 1.96 (h, $^3J_{HH}$ = 7.4 Hz, 2H, H_k), 1.00 (t, $^3J_{HH}$ = 7.4 Hz, 3H, H_l) ppm. **^{13}C{^{1}H} NMR** (125 MHz; CD_2Cl_2): δ_C 194.0, 143.3, 141.1, 140.9, 137.7, 134.8, 128.1, 126.7, 125.8, 122.4, 122.3, 120.5, 120.3, 118.9, 118.2, 108.8, 107.4, 44.6, 30.0, 22.2, 11.5 ppm. **Acc-MS**(ASAP$^+$): *m/z* 360.1406 $[M+H]^+$ calcd. for $C_{23}H_{22}NOS$ *m/z* 360.1422 (|Δ*m/z*| = 4.4 ppm). **Anal. Calc.** for $C_{23}H_{21}NOS$: C, 76.85; H, 5.89; N, 3.90%. **Found**: C, 76.93; H, 5.79; N, 4.10 %.

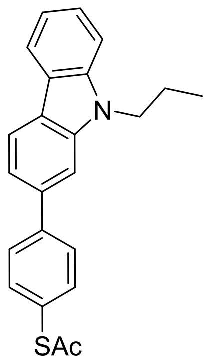

S-(4-(9-propyl-carbazol-3-yl)phenyl) ethanethioate (**2b**). A colorless oil. **Yield**: 0.20 g (47%).**^{1}H NMR** (500 MHz; CD_2Cl_2): δ_H 8.40 (d, $^3J_{HH}$ = 1.7 Hz, 1H, H_a), 8.20 (d, $^3J_{HH}$ = 7.8 Hz, 1H, H_d), 7.84-7.78 (m, 3H, H_b+H_h), 7.56-7.50 (m, 5H, H_c+H_f+H_g+H_i), 7.29 (t, $^3J_{HH}$ = 7.8 Hz, 1H, H_e), 4.36 (t, $^3J_{HH}$ = 7.2 Hz, H_j), 2.48 (s, 3H, H_m), 1.98 (h, $^3J_{HH}$ = 7.3 Hz, 2H, H_k), 1.02 (t, $^3J_{HH}$ = 7.4 Hz, 3H, H_l) ppm. **^{13}C{^{1}H} NMR** (125 MHz; CD_2Cl_2): δ_C 194.3, 143.2, 141.0, 140.3, 134.9,

130.7, 127.7, 125.9, 125.8, 124.9, 123.2, 122.7, 120.3, 118.9, 118.8, 109.2, 109.0, 44.7, 30.0, 22.3, 11.5 ppm. **Acc-MS**(ASAP$^+$): *m/z* 360.1420 [M+H]$^+$ calcd. for $C_{23}H_{22}NOS$ *m/z* 360.1422 (|Δ*m/z*| = 0.6 ppm). **Anal. Calc.** for $C_{23}H_{21}NOS$: C, 76.85; H, 5.89; N, 3.90%. **Found**: C, 76.41; H, 5.90; N, 3.85 %.

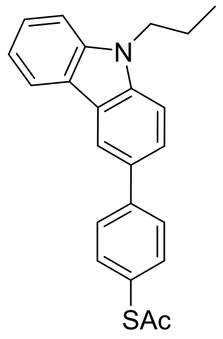

((((5-(tert-butyl)-1,3-phenylene)bis(methylene))bis(carbazole-9,2-diyl))bis(4,1-phenylene)) diethanethioate (**3a**). A white solid. **Yield**: 0.16 g (30%). **^{1}H NMR** (700 MHz; CD_2Cl_2): δ_H 8.09 (dd, $^3J_{HH}$ = 7.8 Hz, $^4J_{HH}$ = 1.0 Hz, 2H, H_a), 8.06 (dt, $^3J_{HH}$ = 7.8 Hz, $^4J_{HH}$ = 1.0 Hz, 2H, H_d), 7.59 (d, $^3J_{HH}$ = 8.3 Hz, 4H, H_h), 7.46-7.44 (m, 8H, H_b+H_c+H_i), 7.30 (ddd, $^3J_{HH}$ = 8.2 Hz, $^3J_{HH}$ = 7.1 Hz, $^4J_{HH}$ = 1.2 Hz, 2H, H_f), 7.23-7.19 (m, 6H, H_e+H_g+H_k), 6.55 (s, 1H, H_j), 5.41 (s, 4H, H_l), 2.46 (s, 6H, H_n), 1.19 (s, 9H, H_m) ppm. **^{13}C{^{1}H} NMR** (176 MHz; CD_2Cl_2): δ_C 194.0, 152.3, 142.9, 1410, 137.6, 134.7, 128.0, 126.6, 125.8, 123.0, 122.5, 121.5, 120.5, 119.3, 118.6, 108.8, 107.1, 46.6, 34.4, 30.8, 30.0 ppm. **Acc-MS**(ASAP$^+$): *m/z* 793.2936 [M+H]$^+$ calcd. for $C_{52}H_{45}N_2$ O_2S_2 *m/z* 793.2922 (|Δ*m/z*| = 1.8 ppm). **Anal. Calc.** for $C_{52}H_{44}N_2O_2S_2$: C, 78.76; H, 5.59; N, 3.53 %. **Found**: C, 78.57; H, 5.60; N, 3.46 %.

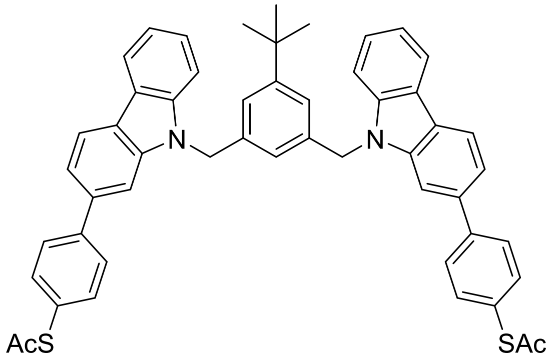

*S,S'-((((5-(*tert*-butyl)-1,3-phenylene)bis(methylene))bis(carbazole-9,3-diyl))bis(4,1-phenylene)) diethanethioate* (**3b**). A white solid. **Yield**: 0.18 g (33%). **^{1}H NMR** (500 MHz; CD_2Cl_2): δ_H 8.33 (d, $^4J_{HH}$ = 1.7 Hz ppm, 2H, H_a), 8.15 (d, $^3J_{HH}$ = 7.7 Hz, 2H, H_d), 7.78 (d, $^3J_{HH}$ = 8.1 Hz, 4H, H_h), 7.62 (dt, $^3J_{HH}$ = 8.5 Hz, $^4J_{HH}$ = 1.4 Hz, 2H, H_b), 7.54 (d, $^3J_{HH}$ = 8.0 Hz, 4H, H_i), 7.39 (dd, $^3J_{HH}$ = 8.3 Hz, $^3J_{HH}$ = 7.0 Hz, 2H, H_f), 7.31-7.25 (m, 8H, H_c+H_e+H_g+H_k), 6.54 (s, 1H, H_j) 5.42 (s, 4H, H_l), 2.50 (s, 6H, H_n), 1.22 (s, 9H, H_m) ppm. **^{13}C{^{1}H} NMR** (125 MHz; CD_2Cl_2): δ_C 194.3, 152.2, 143.0, 140.9, 140.2, 137.4, 135.0, 131.0, 127.6, 126.0, 125.9, 125.0, 123.4, 123.0, 122.8, 121.7, 120.3, 119.3, 118.8, 109.1, 108.9, 46.6, 34.4, 30.9, 30.0 ppm. **Acc-MS**(ASAP$^+$): *m/z* 793.2920 $[M+H]^+$ calcd. for $C_{52}H_{45}N_2$ O_2S_2 *m/z* 793.2922 ($|\Delta m/z|$ = 0.3 ppm). **Anal. Calc.** for $C_{52}H_{44}N_2O_2S_2$: C, 78.76; H, 5.59; N, 3.53 %. **Found**: C, 78.41; H, 5.36; N, 3.47 %.

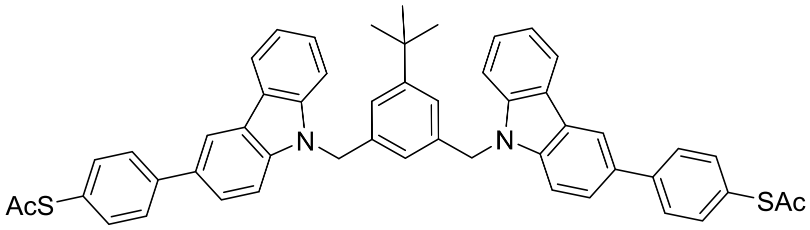

HPLC Chromatograms

HPLC purification was performed using an Interchimic PuriFlash prep HPLC using Puriflash 12 g, HC Spherical Silica, 15µm, Flash LL Columns eluted using HPLC quality solvents from standard suppliers, running at 28 mL/min.

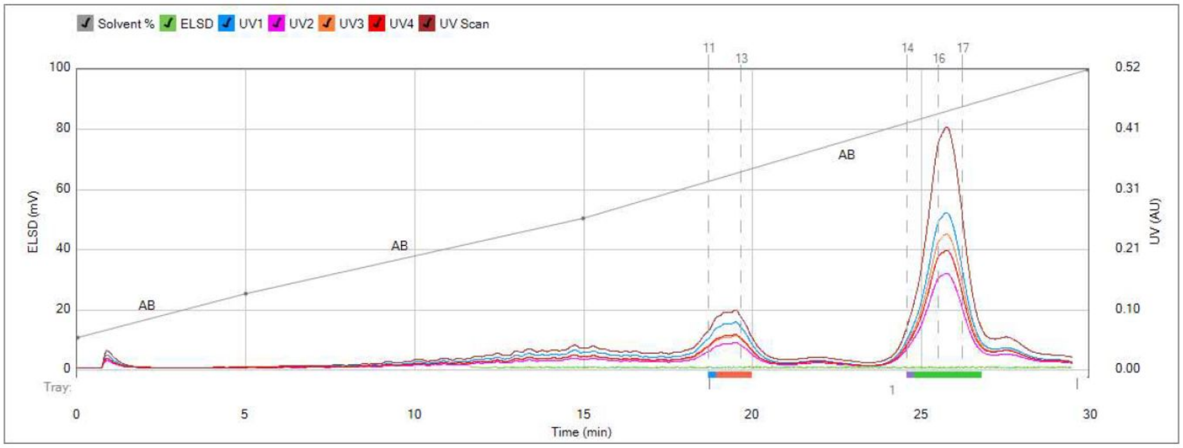

Figure S1. HPLC chromatogram of **1b-TMS** where solvent A = hexane and solvent B = DCM.

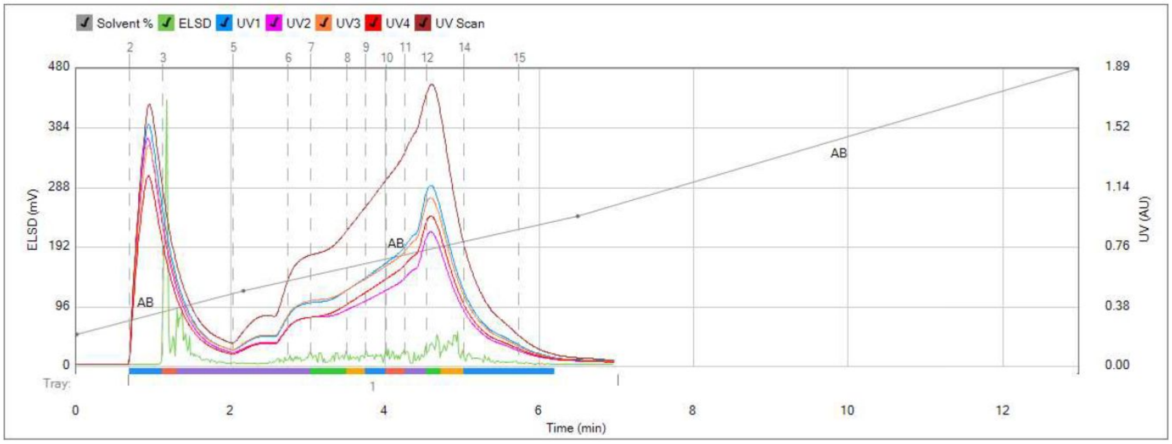

Figure S2. HPLC chromatogram of **2b-TMS** where solvent A = hexane and solvent B = DCM.

S2. NMR spectra of reported compounds

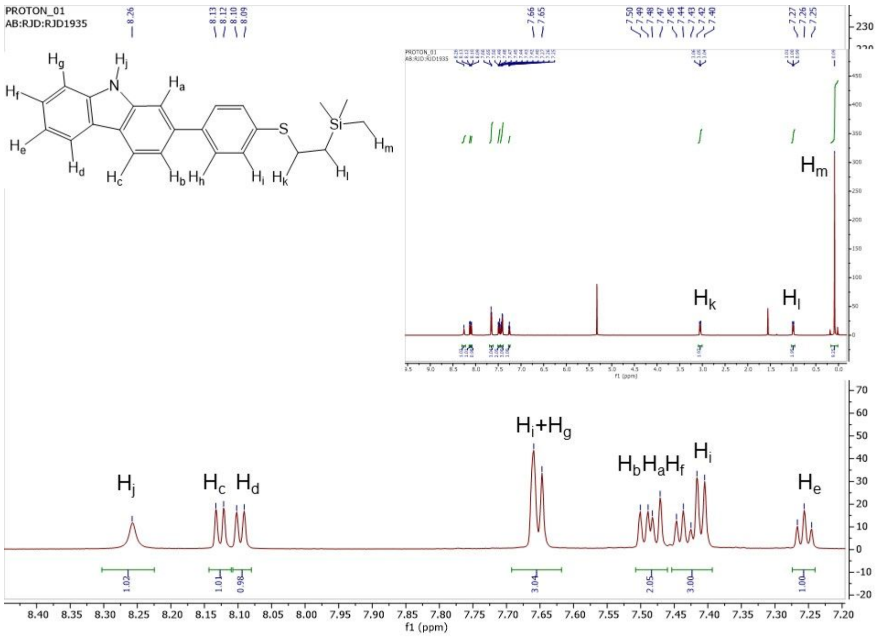

Figure S3. ^{1}H NMR spectra of 2-(4-((2-(trimethylsilyl)ethyl)thio)phenyl)-carbazole recorded in CD_2Cl_2.

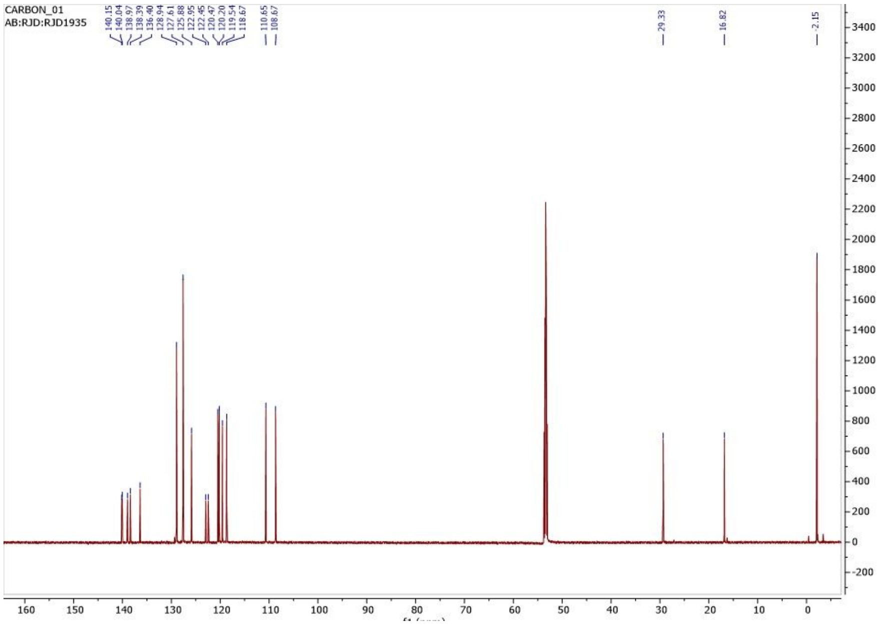

Figure S4. $^{13}C\{^{1}H\}$ NMR spectrum of 2-(4-((2-(trimethylsilyl)ethyl)thio)phenyl)-carbazole recorded in CD_2Cl_2.

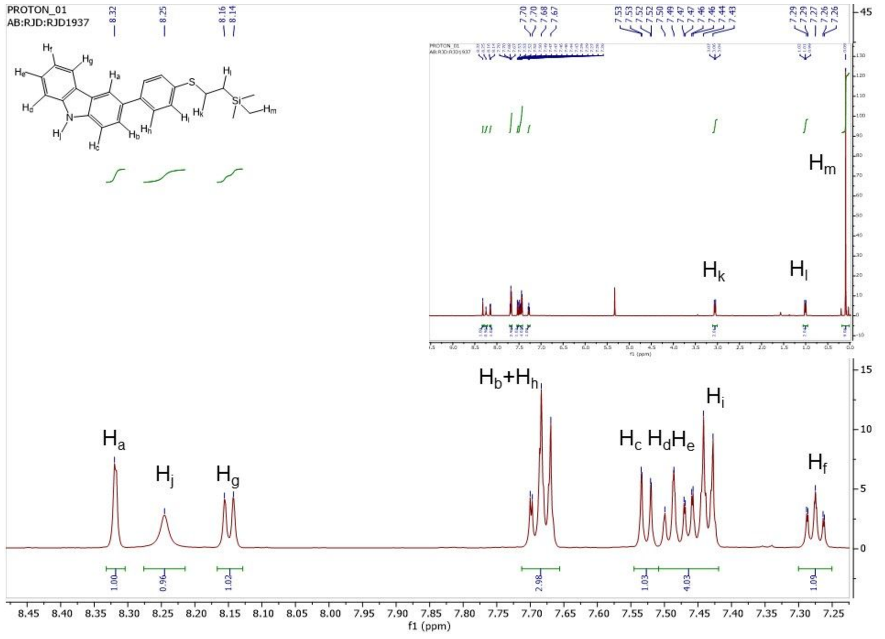

Figure S5. ^{1}H NMR spectra of 3-(4-((2-(trimethylsilyl)ethyl)thio)phenyl)-9*H*-carbazole recorded in CD_2Cl_2.

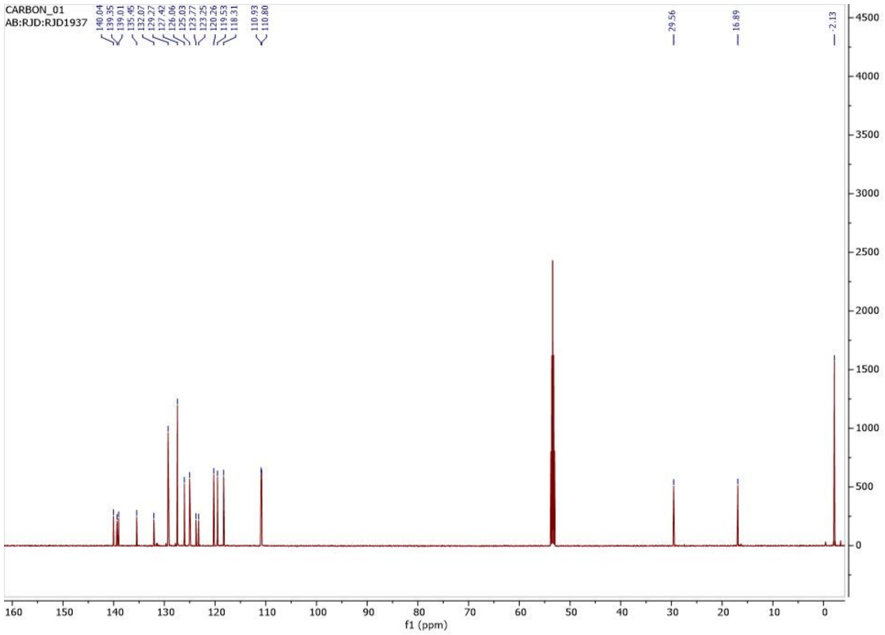

Figure S6. $^{13}C\{^{1}H\}$ NMR spectrum of 3-(4-((2-(trimethylsilyl)ethyl)thio)phenyl)-9*H*-carbazole recorded in CD_2Cl_2.

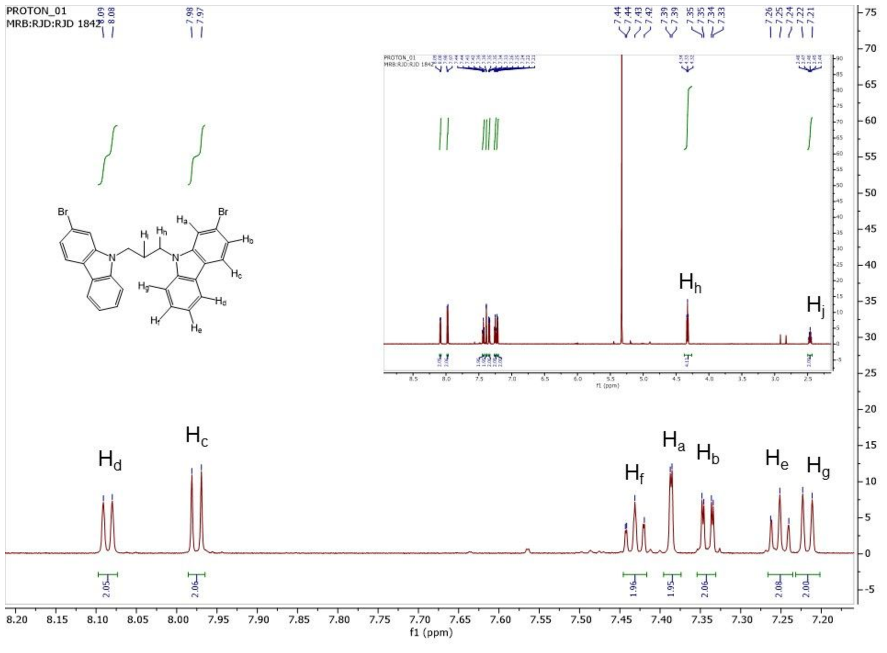

Figure S7. ^{1}H NMR spectra of 1,3-bis(2-bromo-9*H*-carbazol-9-yl)propane recorded in CD_2Cl_2.

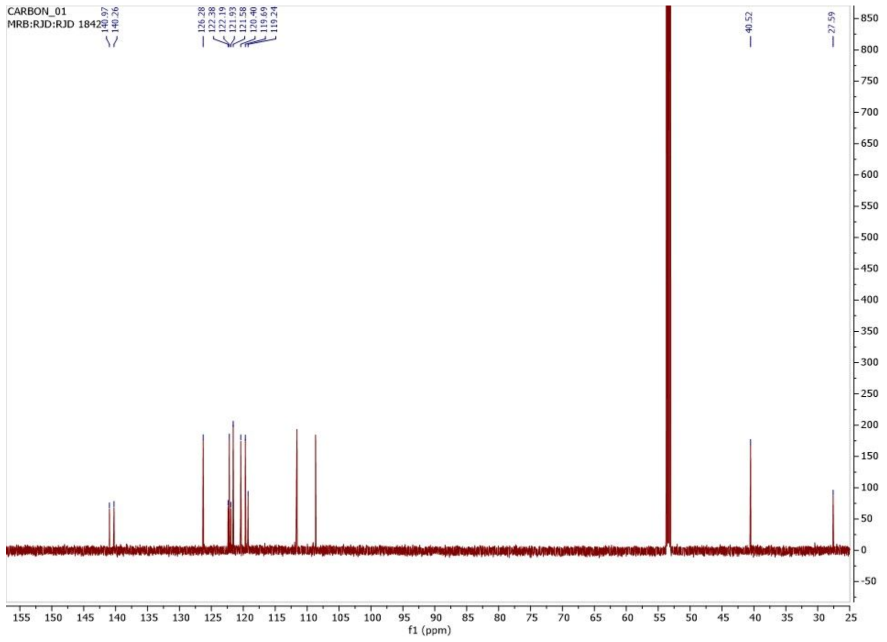

Figure S8. $^{13}C\{^{1}H\}$ NMR spectrum of 1,3-bis(2-bromo-9*H*-carbazol-9-yl)propane recorded in CD_2Cl_2.

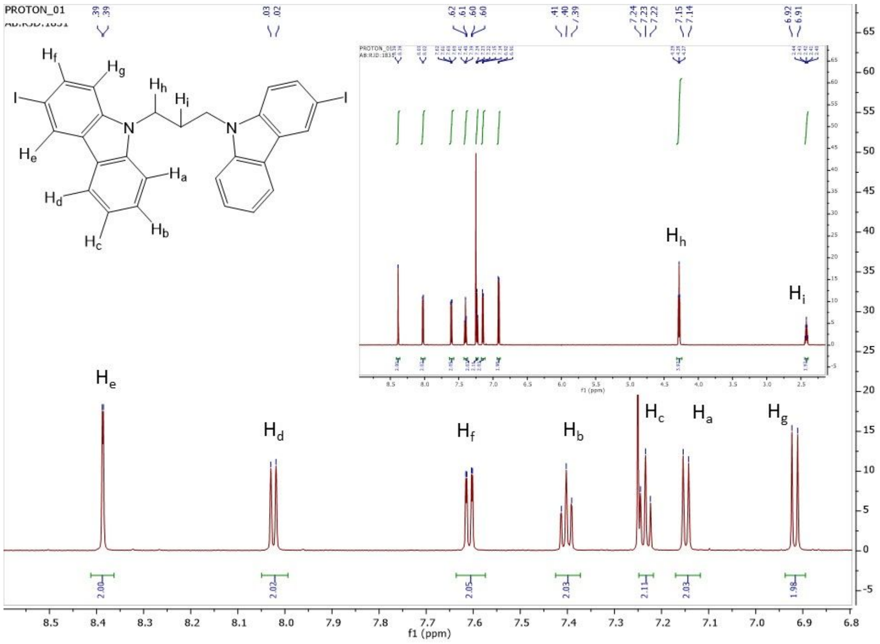

Figure S9. ^{1}H NMR spectra of 1,3-bis(3-iodo-carbazol-9-yl)propane recorded in $CDCl_3$.

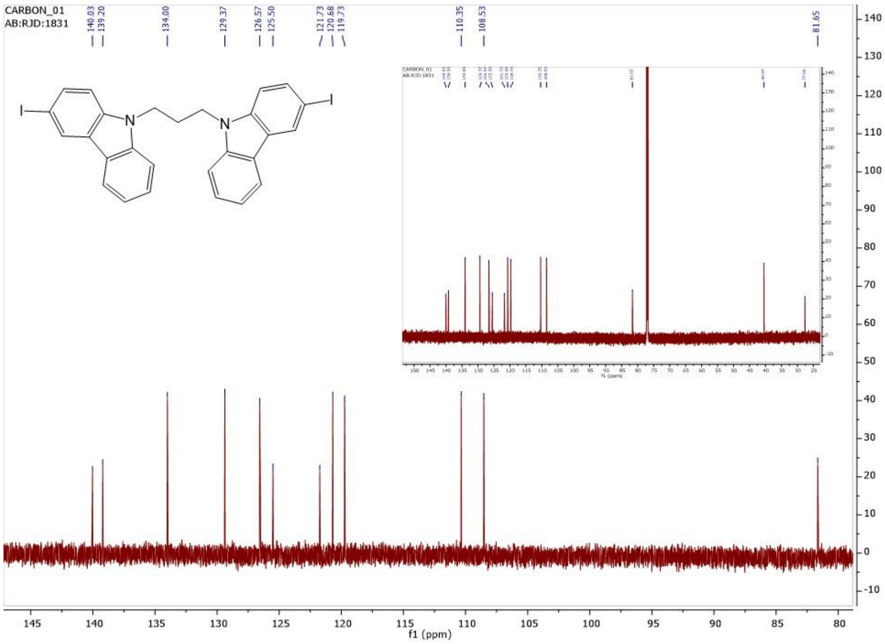

Figure S10. $^{13}C\{^{1}H\}$ NMR spectra of 1,3-bis(3-iodo-carbazol-9-yl)propane recorded in $CDCl_3$.

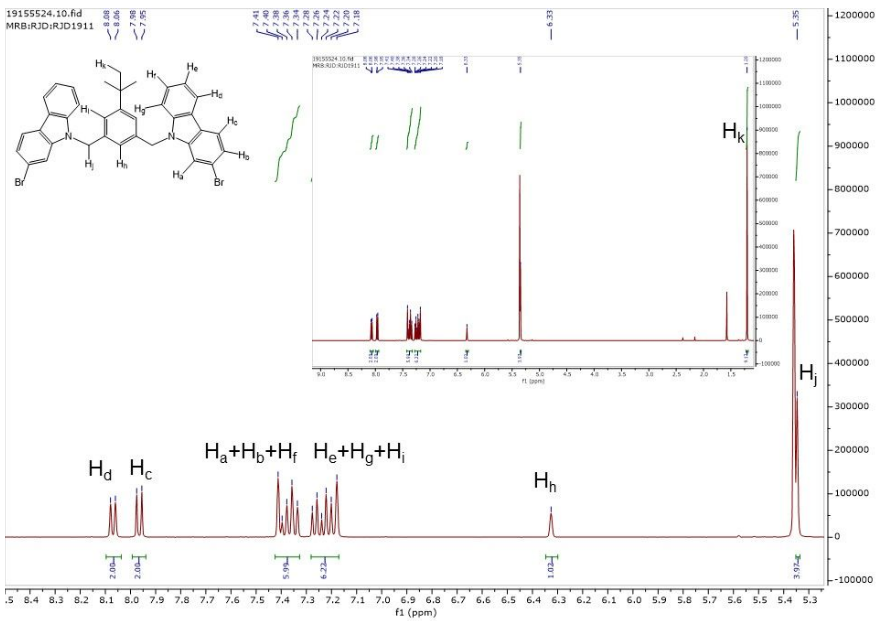

Figure S11. ^{1}H NMR spectra of 9,9'-((5-(*tert*-butyl)-1,3-phenylene)bis(methylene))bis(2-bromo-carbazole) recorded in CD_2Cl_2.

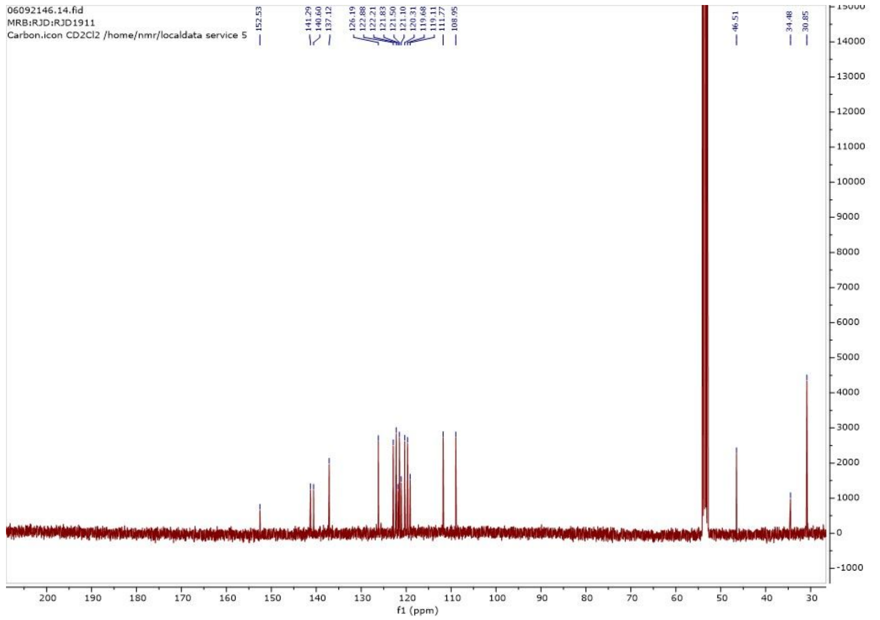

Figure S12. $^{13}C\{^{1}H\}$ NMR spectrum of 9,9'-((5-(*tert*-butyl)-1,3-phenylene)bis(methylene))bis(2-bromo-carbazole) recorded in CD_2Cl_2.

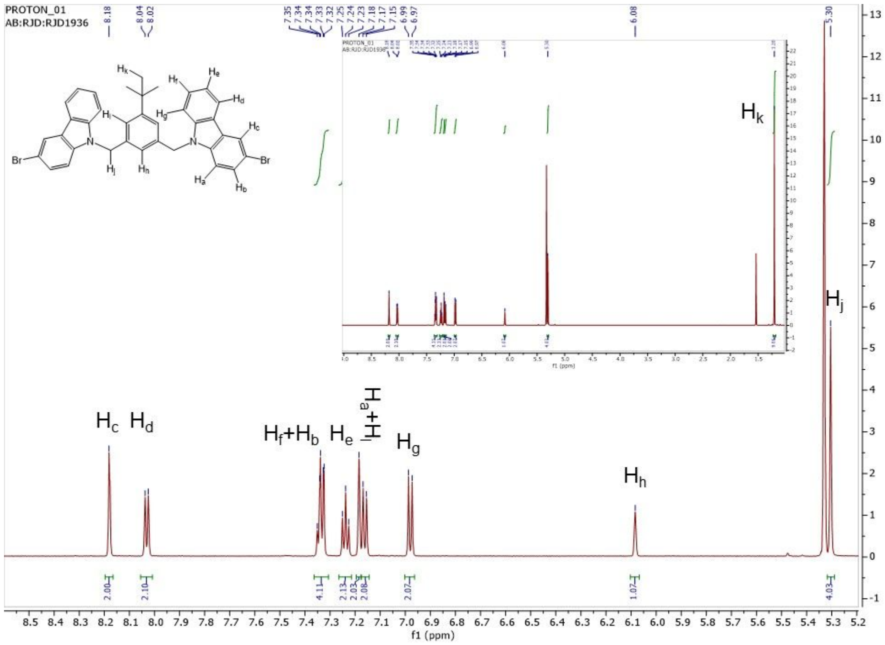

Figure S13. ^{1}H NMR spectra of 9,9'-((5-(*tert*-butyl)-1,3-phenylene)bis(methylene))bis(3-bromo-carbazole) recorded in CD_2Cl_2.

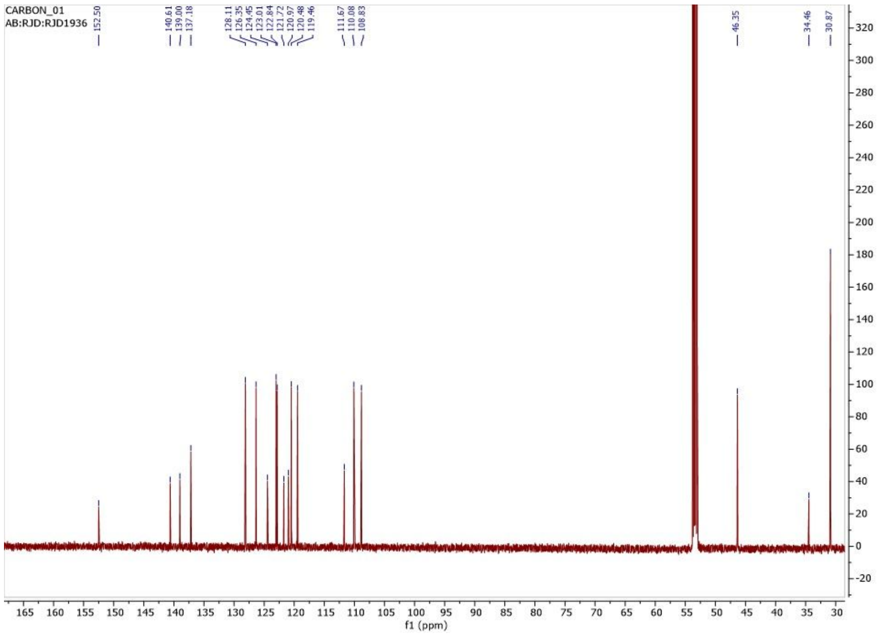

Figure S14. $^{13}C\{^{1}H\}$ NMR spectrum of 9,9'-((5-(*tert*-butyl)-1,3-phenylene)bis(methylene))bis(3-bromo-carbazole) recorded in CD_2Cl_2.

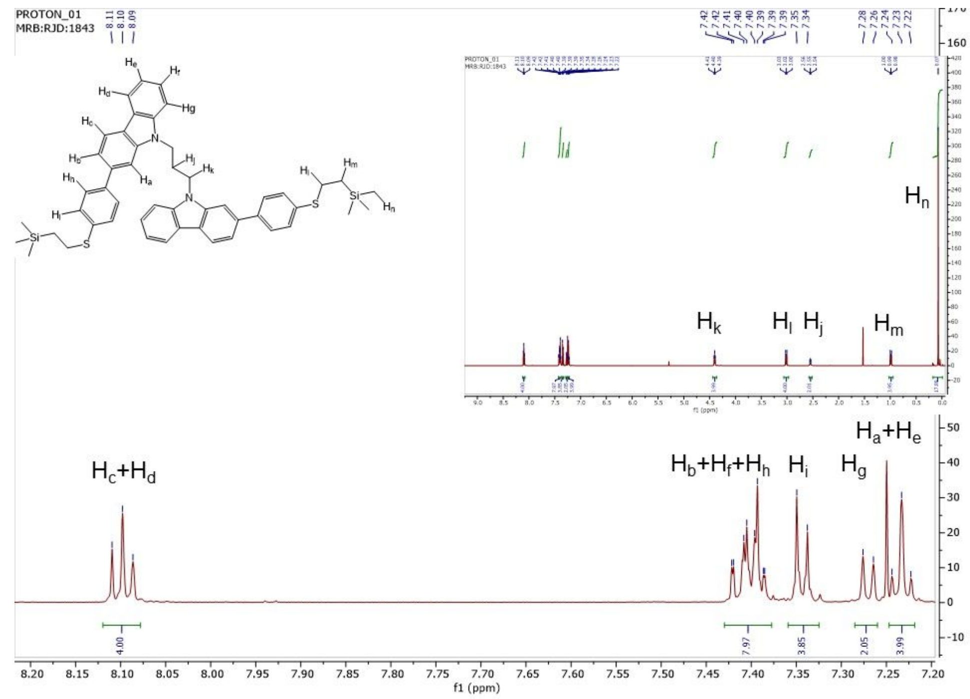

Figure S15. ^{1}H NMR spectra of **1a-TMS** recorded in $CDCl_3$.

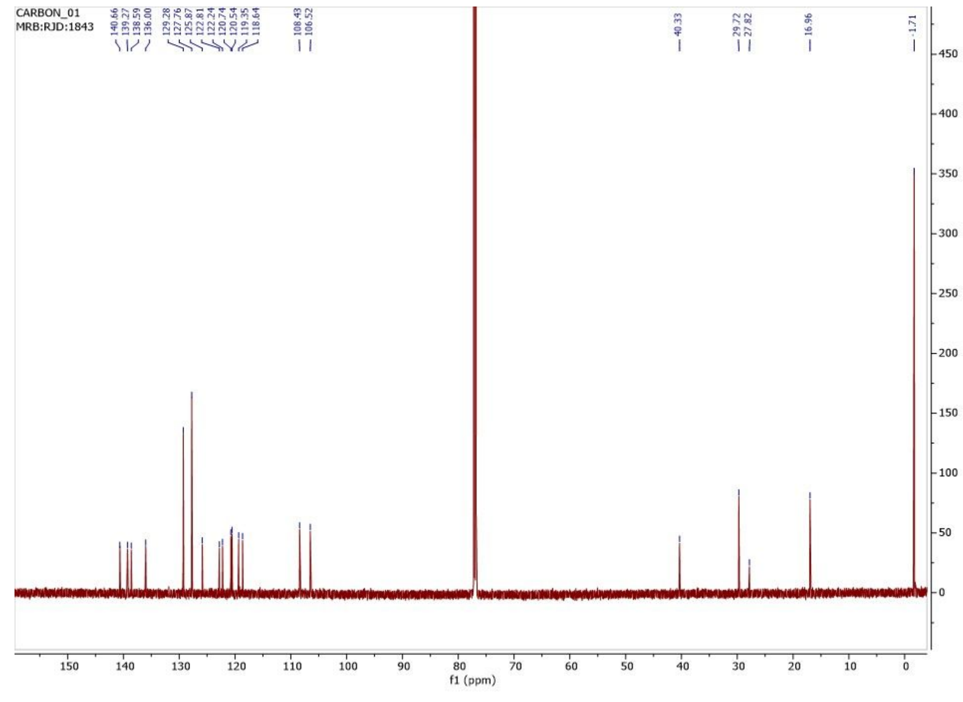

Figure S16. $^{13}C\{^{1}H\}$ NMR spectrum of **1a-TMS** recorded in $CDCl_3$.

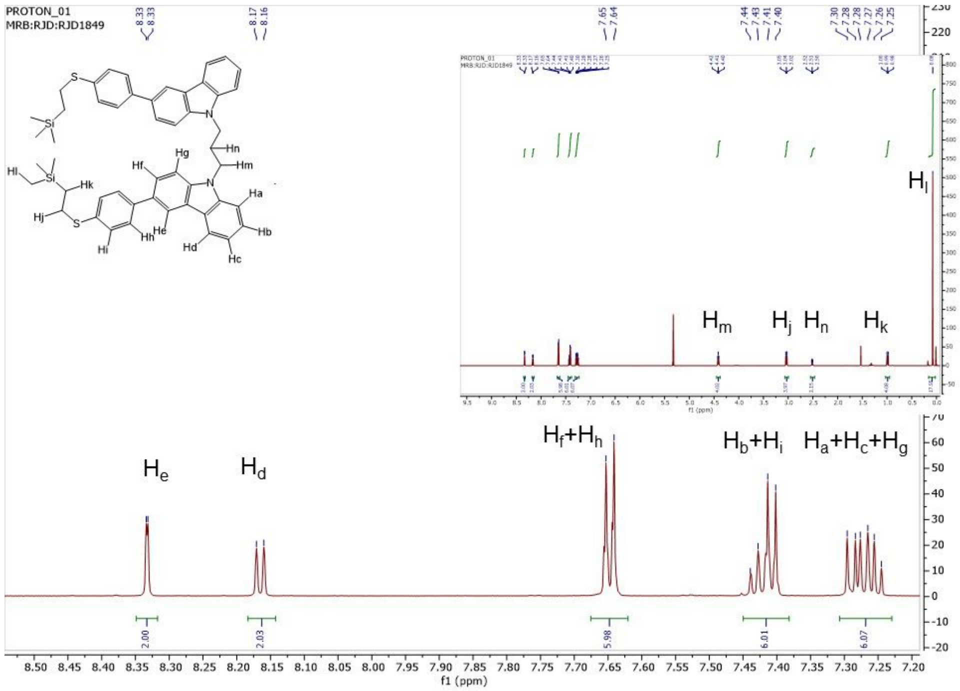

Figure S17. ^{1}H NMR spectra of **1b-TMS** recorded in CD_2Cl_2.

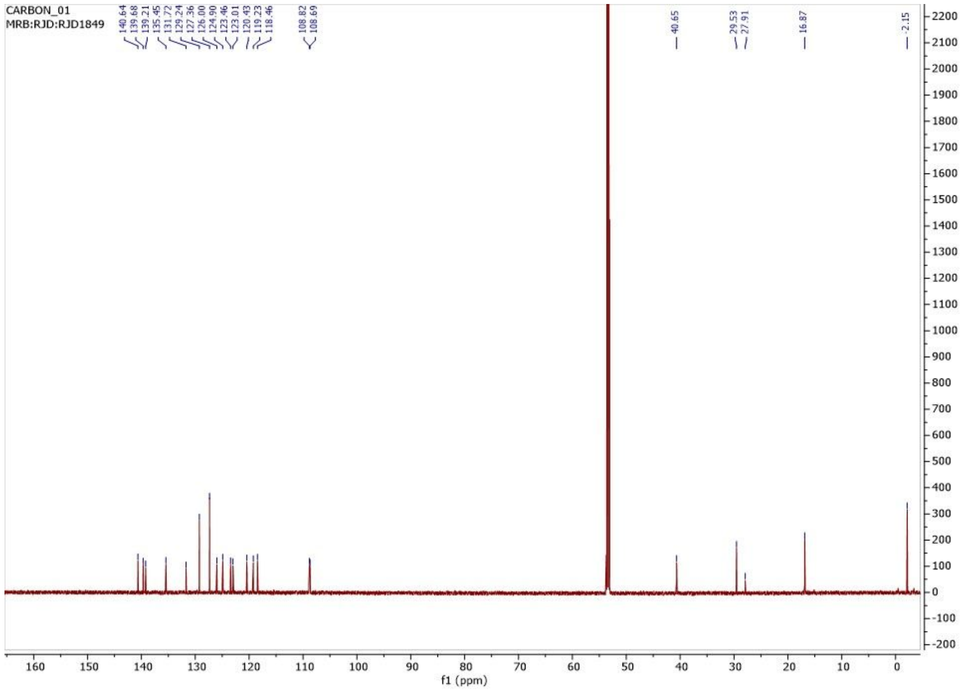

Figure S18. $^{13}C\{^1H\}$ NMR spectrum of **1b-TMS** recorded in CD_2Cl_2.

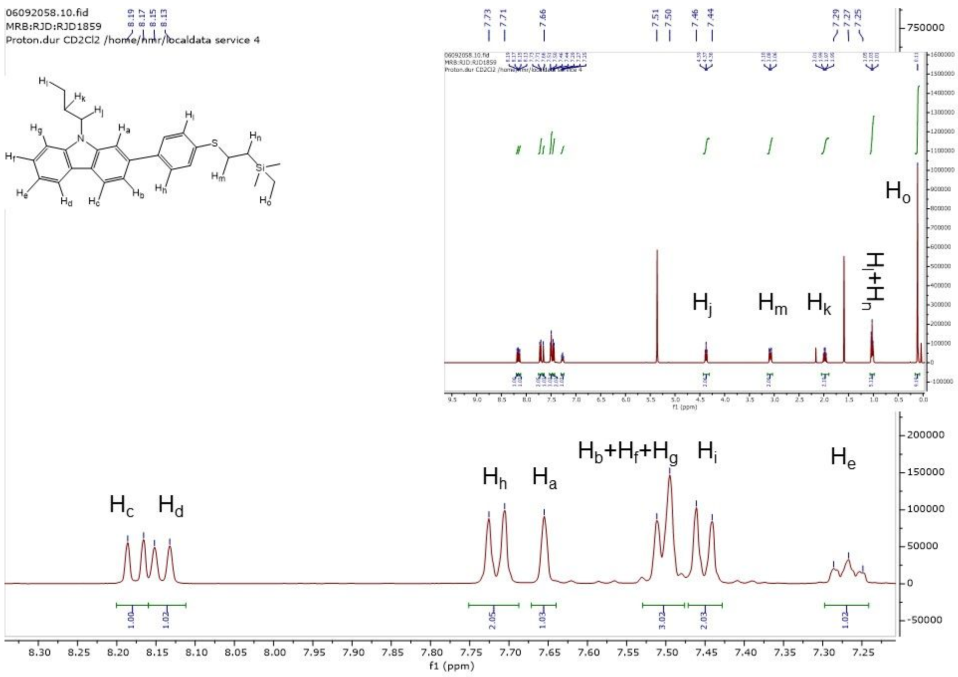

Figure S19. ^{1}H NMR spectra of **2a-TMS** recorded in CD_2Cl_2.

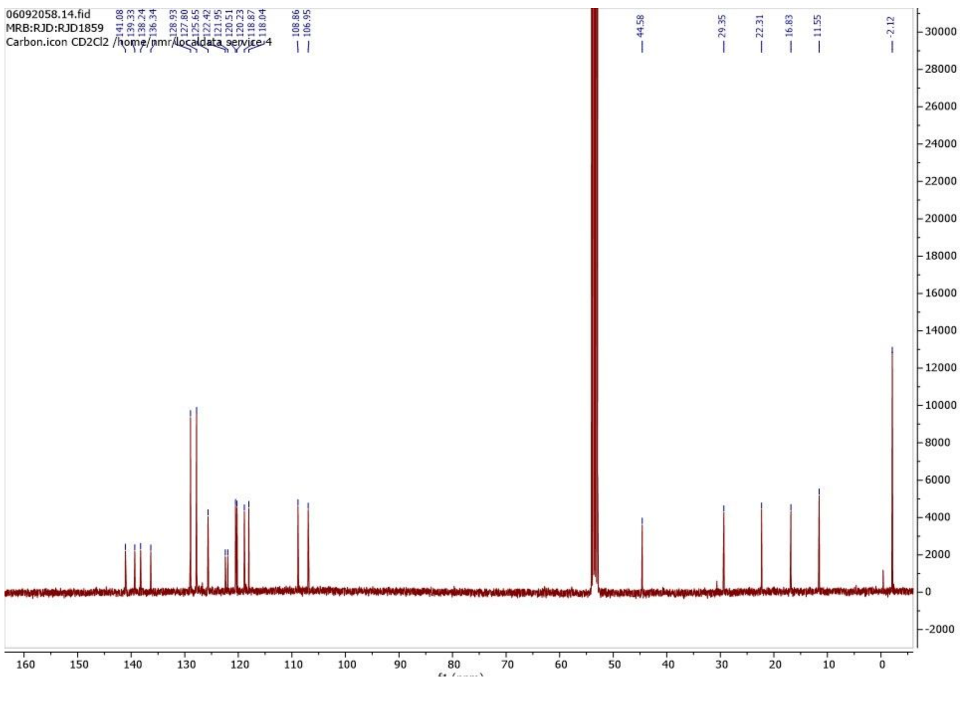

Figure S20. $^{13}C\{^1H\}$ NMR spectrum of **2a-TMS** recorded in CD_2Cl_2.

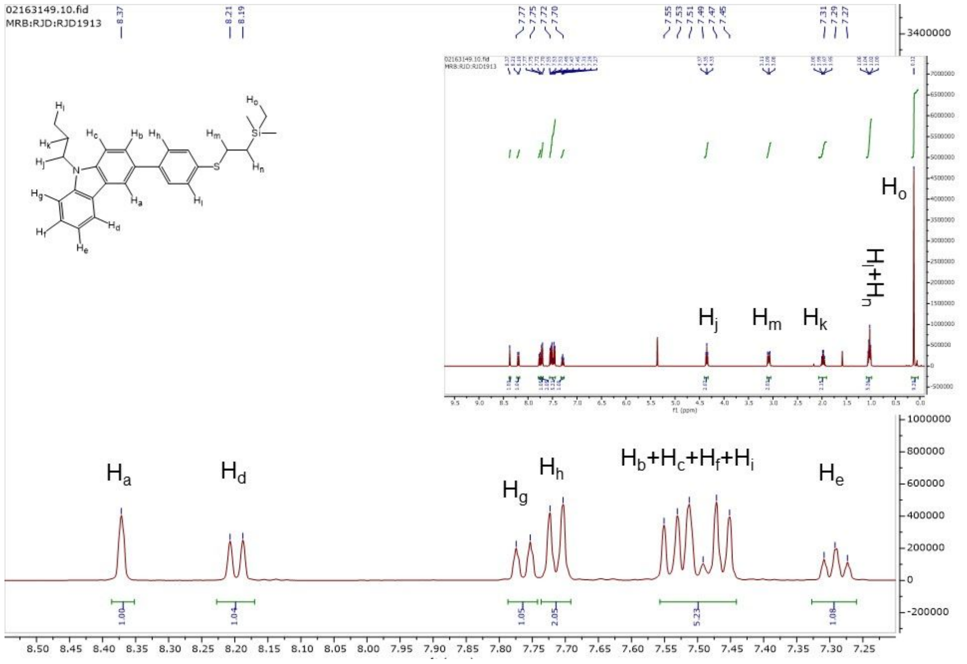

Figure S21. ^{1}H NMR spectra of **2b-TMS** recorded in CD_2Cl_2.

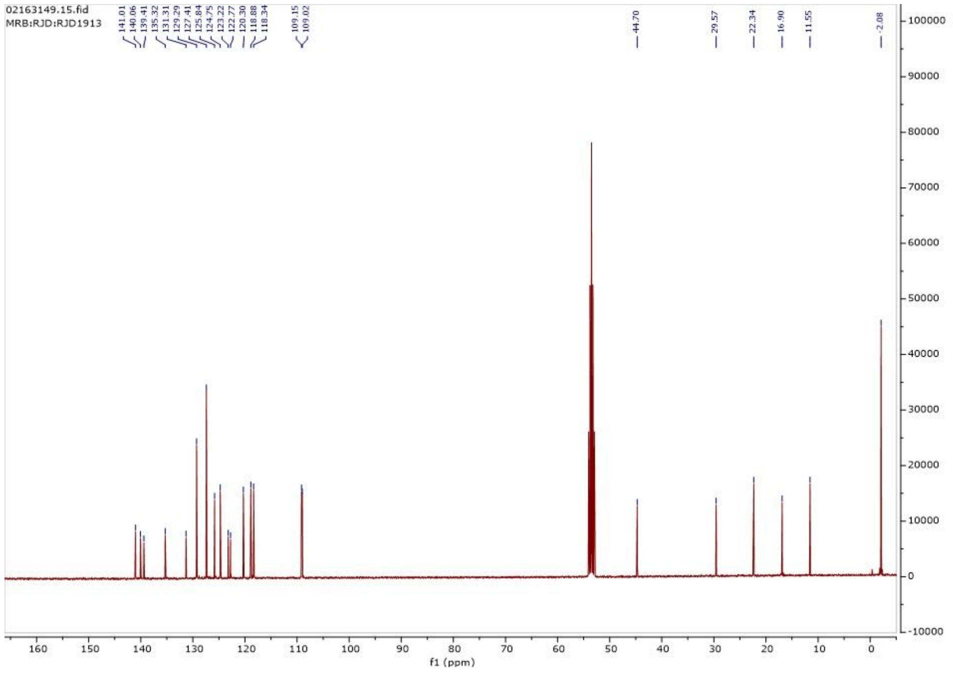

Figure S22. $^{13}C\{^{1}H\}$ NMR spectrum of **2b-TMS** recorded in CD_2Cl_2.

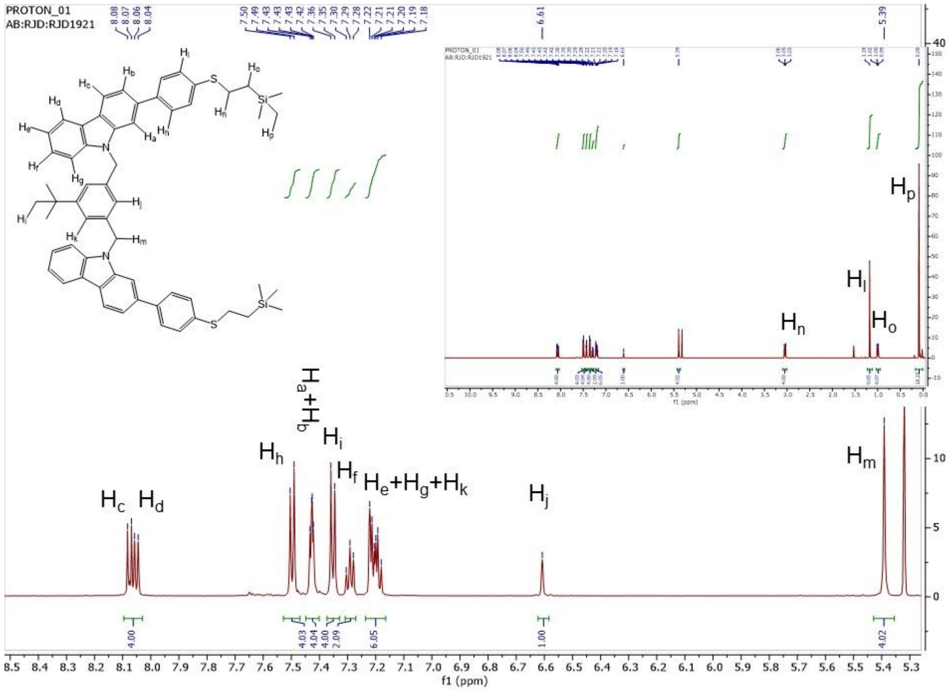

Figure S23. ^{1}H NMR spectra of **3a-TMS** recorded in CD_2Cl_2.

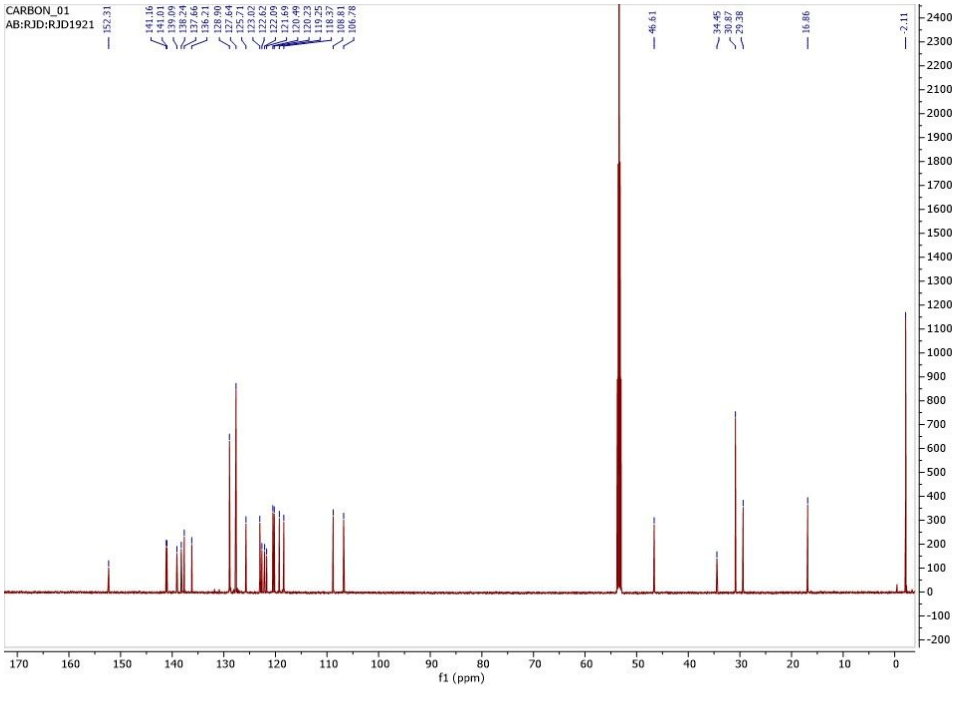

Figure S24. $^{13}C\{^{1}H\}$ NMR spectrum of **3a-TMS** recorded in CD_2Cl_2.

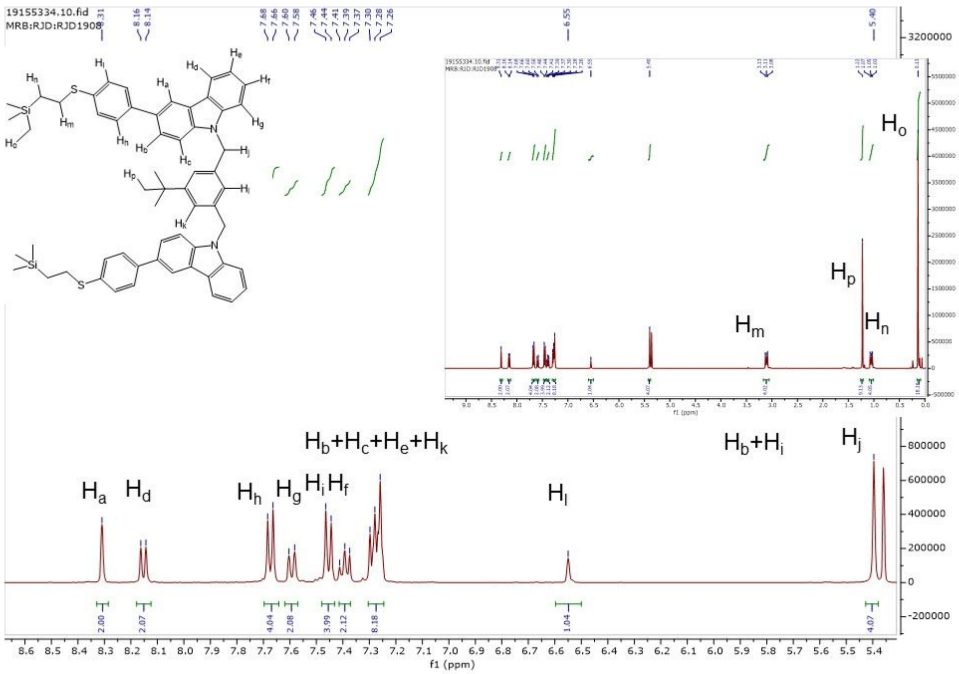

Figure S25. ^{1}H NMR spectra of **3b-TMS** recorded in CD_2Cl_2.

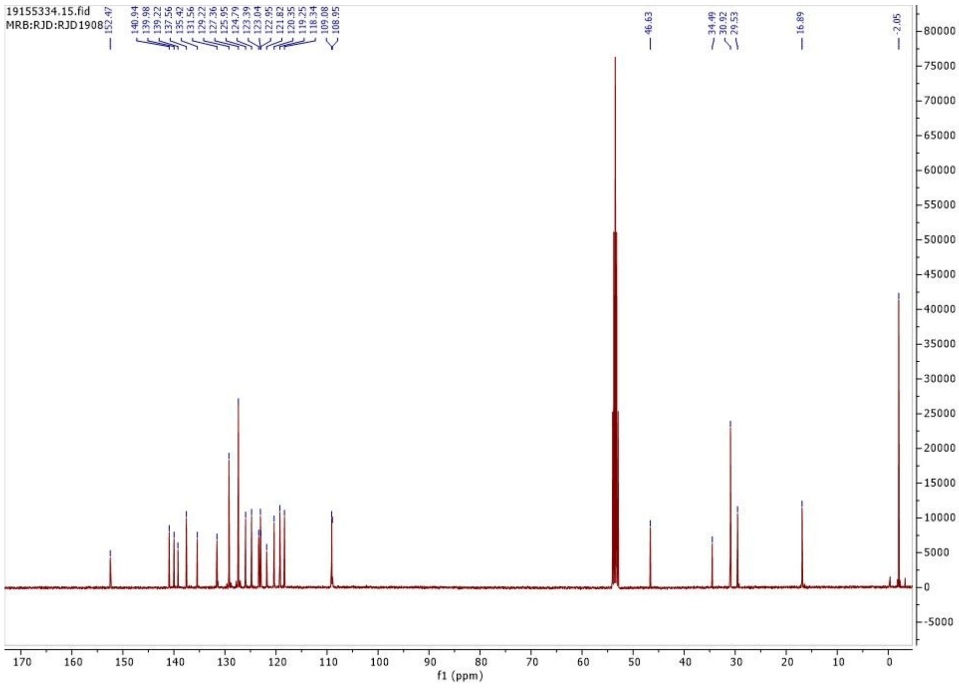

Figure S26. $^{13}C\{^1H\}$ NMR spectrum of **3b-TMS** recorded in CD_2Cl_2.

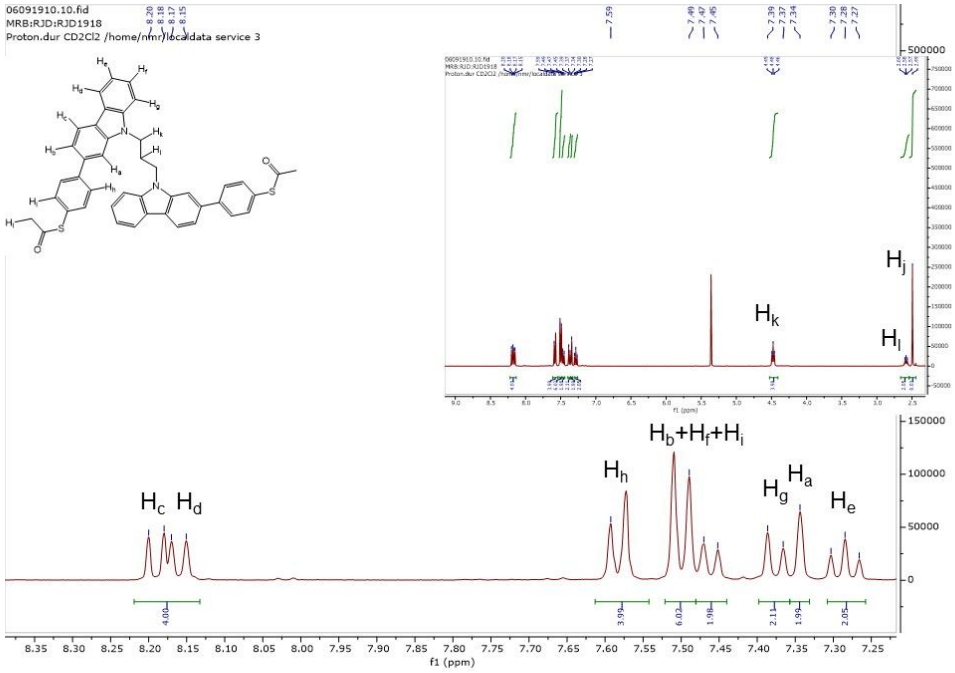

Figure S27. ^{1}H NMR spectra of **1a** recorded in CD_2Cl_2.

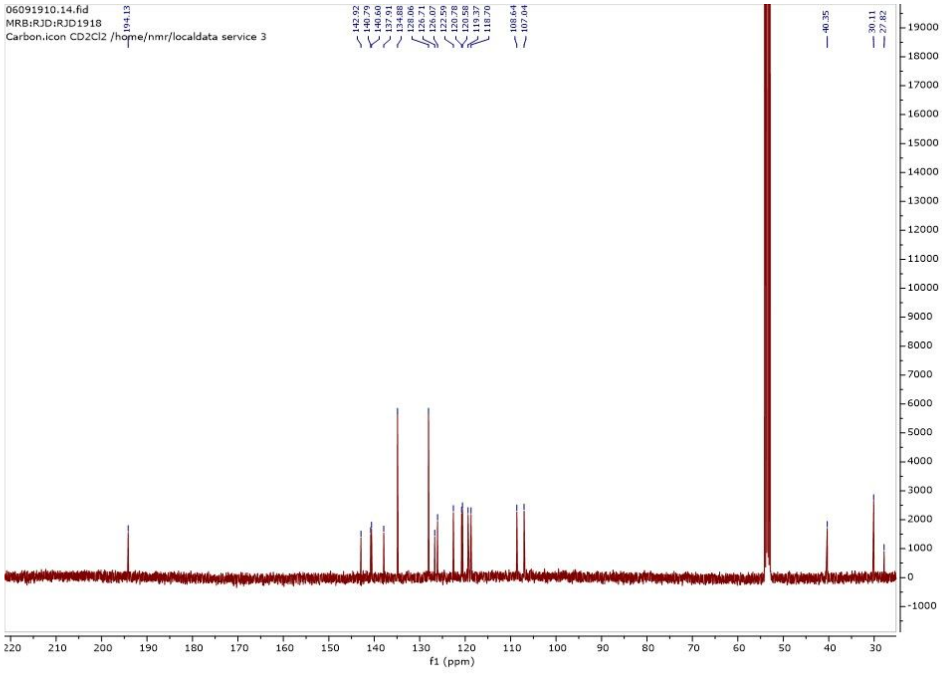

Figure S28. $^{13}C\{^{1}H\}$ NMR spectrum of **1a** recorded in CD_2Cl_2.

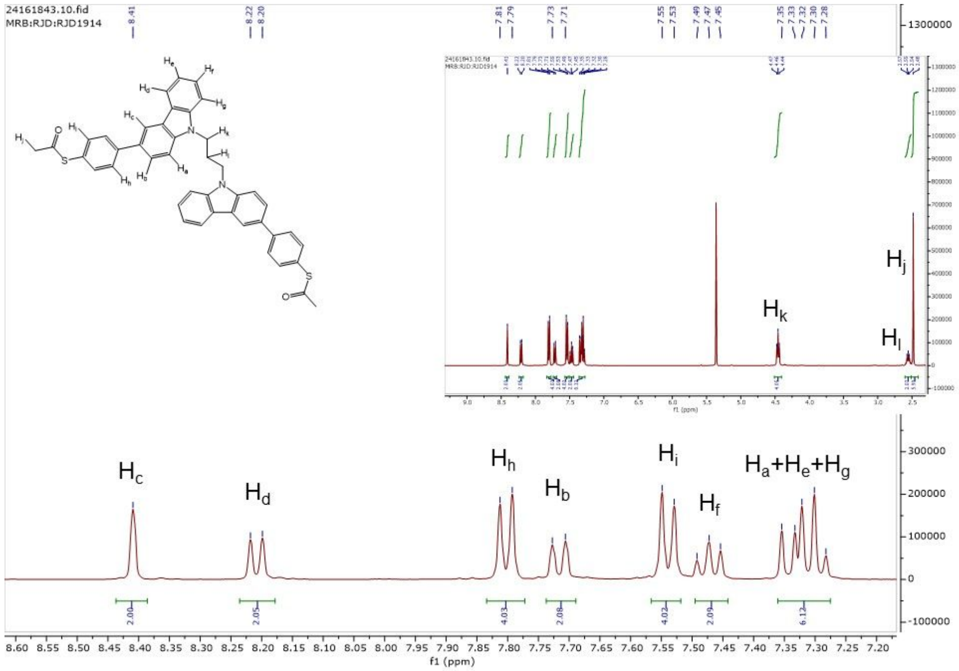

Figure S29. ^{1}H NMR spectra of **1b** recorded in CD_2Cl_2.

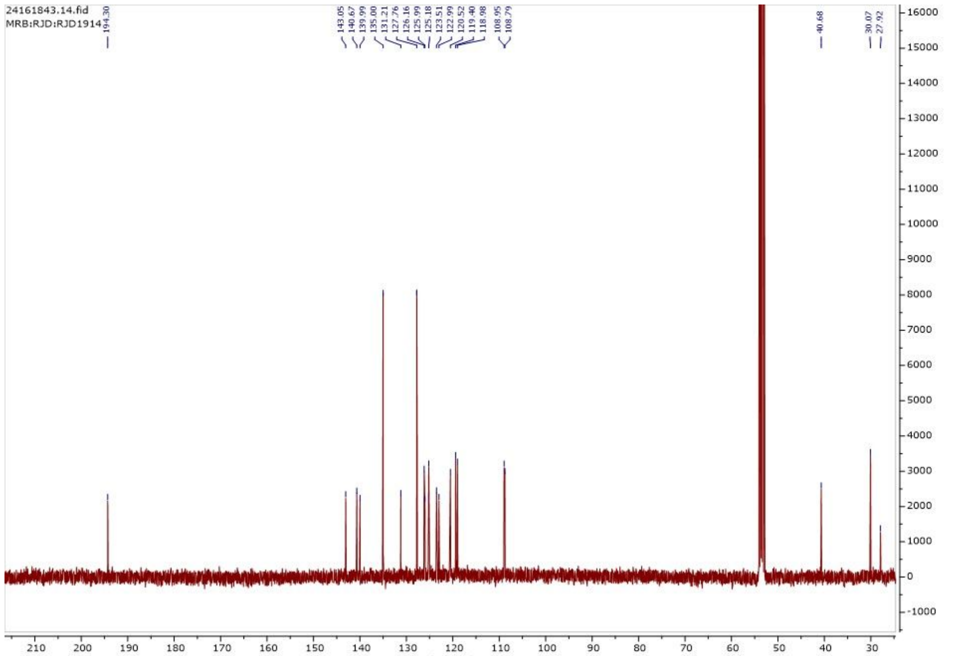

Figure S30. $^{13}C\{^{1}H\}$ NMR spectrum of **1b** recorded in CD_2Cl_2.

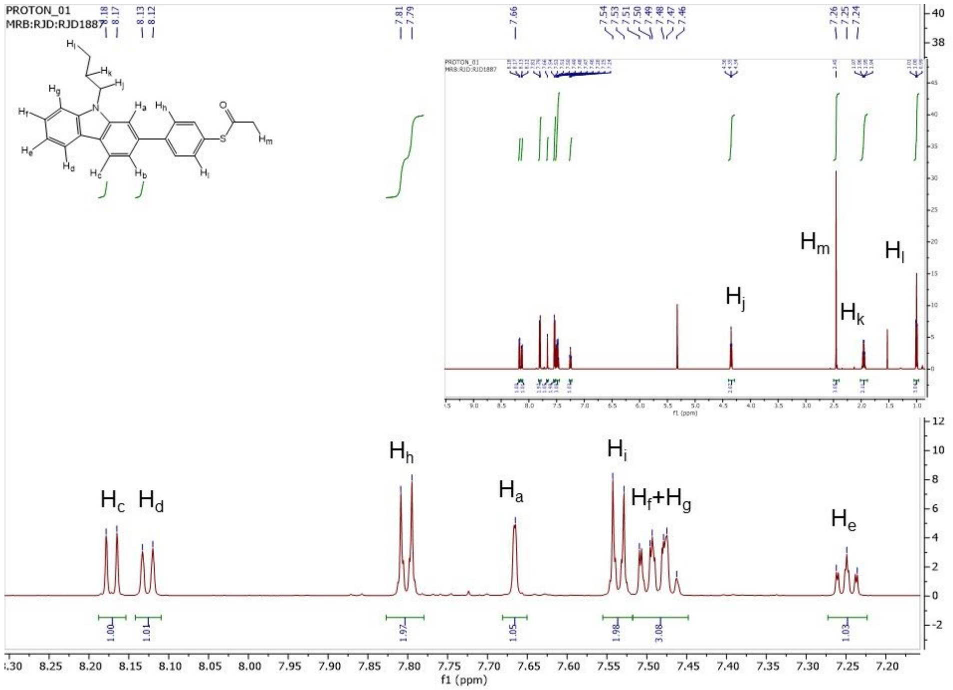

Figure S31. ^{1}H NMR spectra of **2a** recorded in CD_2Cl_2.

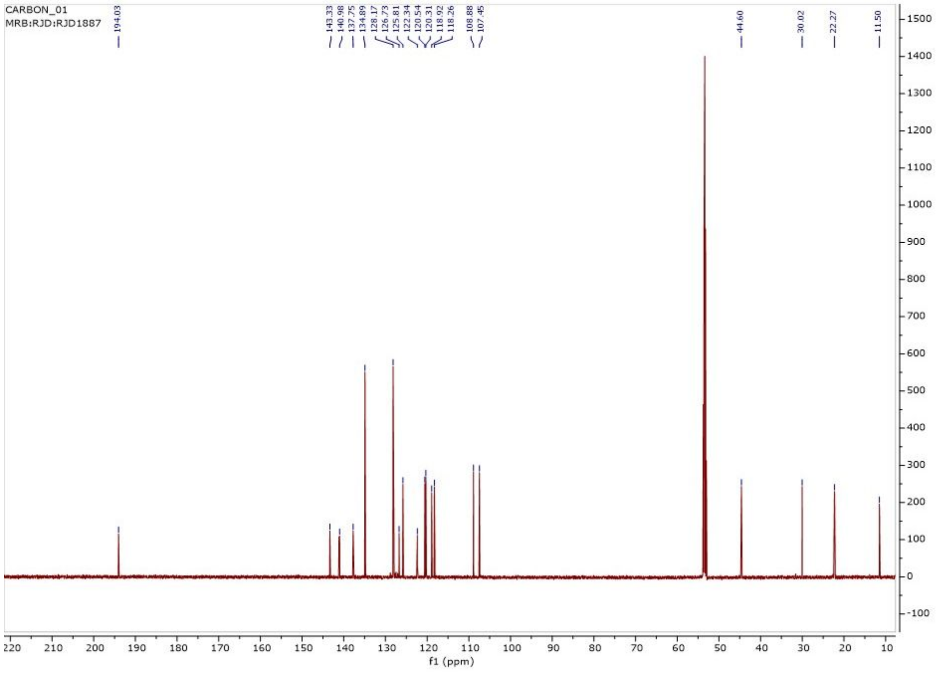

Figure S32. $^{13}C\{^{1}H\}$ NMR spectrum of **2a** recorded in CD_2Cl_2.

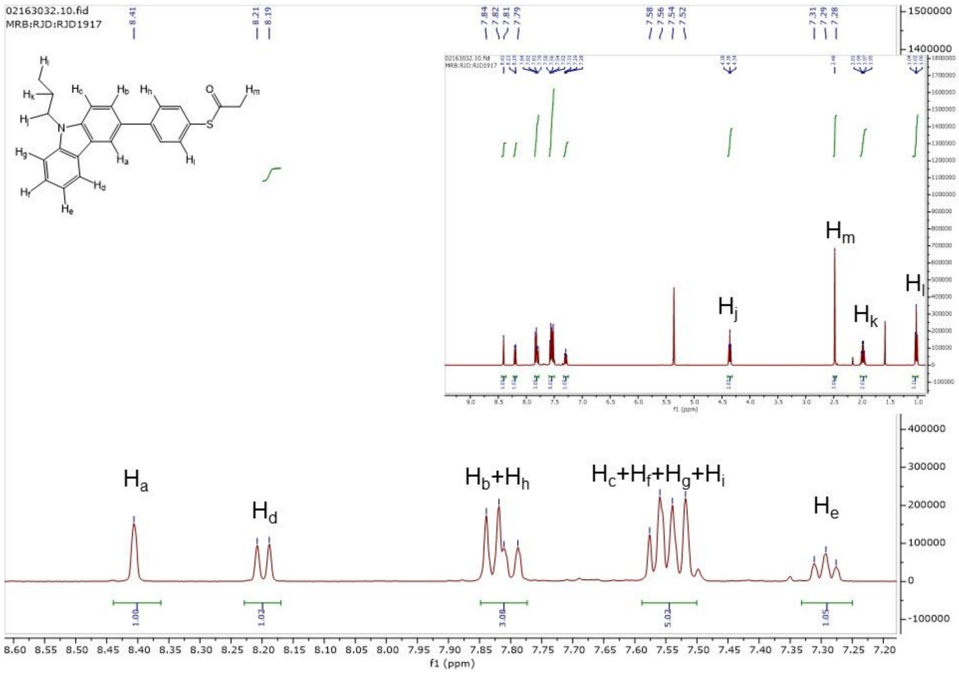

Figure S33. ^{1}H NMR spectra of **2b** recorded in CD_2Cl_2.

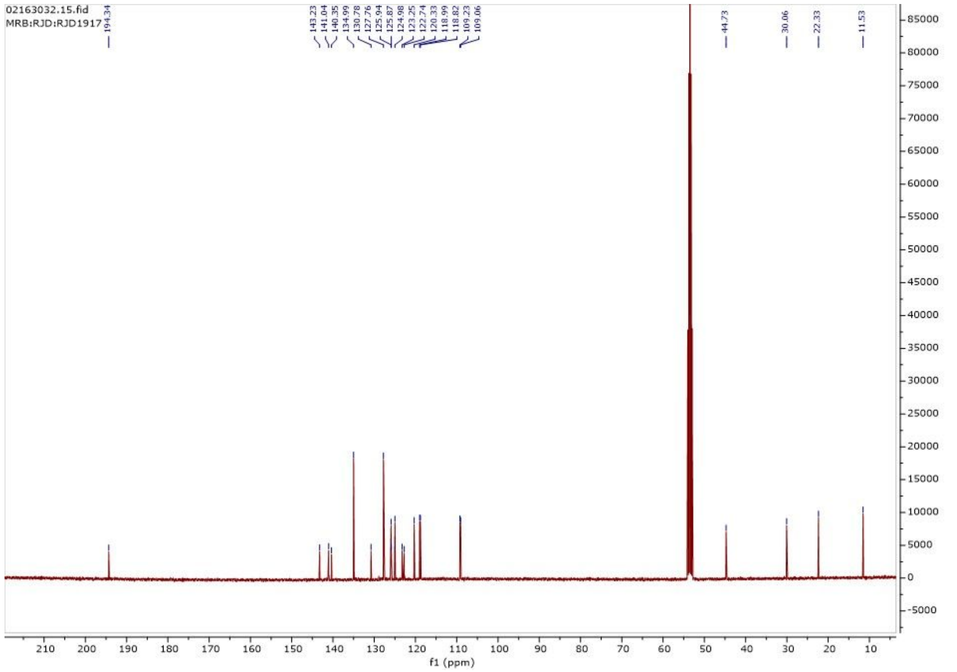

Figure S34. $^{13}C\{^{1}H\}$ NMR spectrum of **2b** recorded in CD_2Cl_2.

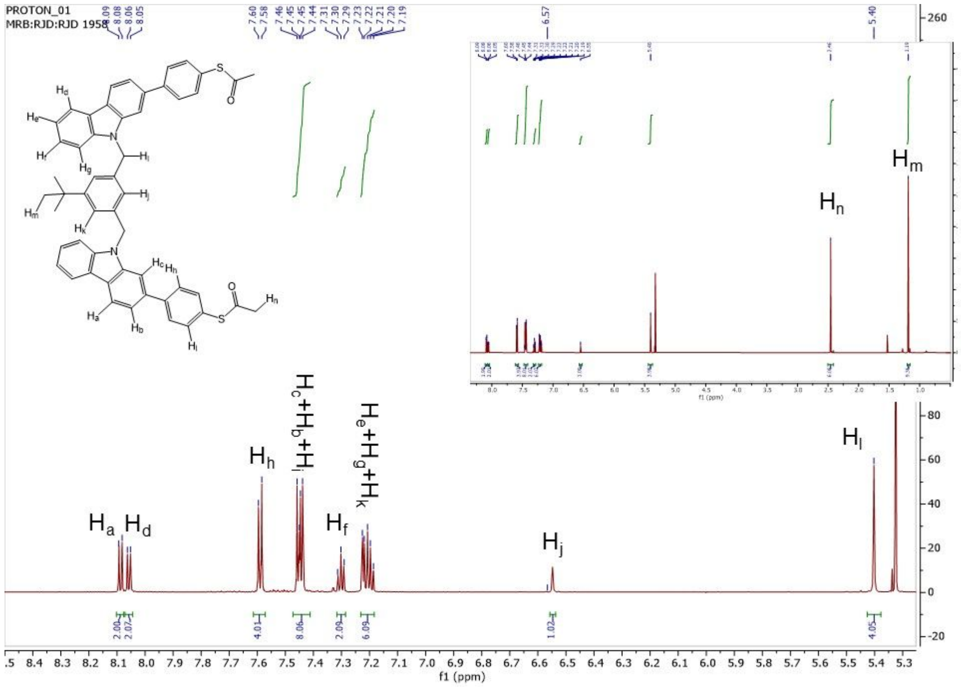

Figure S35. ^{1}H NMR spectra of **3a** recorded in CD_2Cl_2.

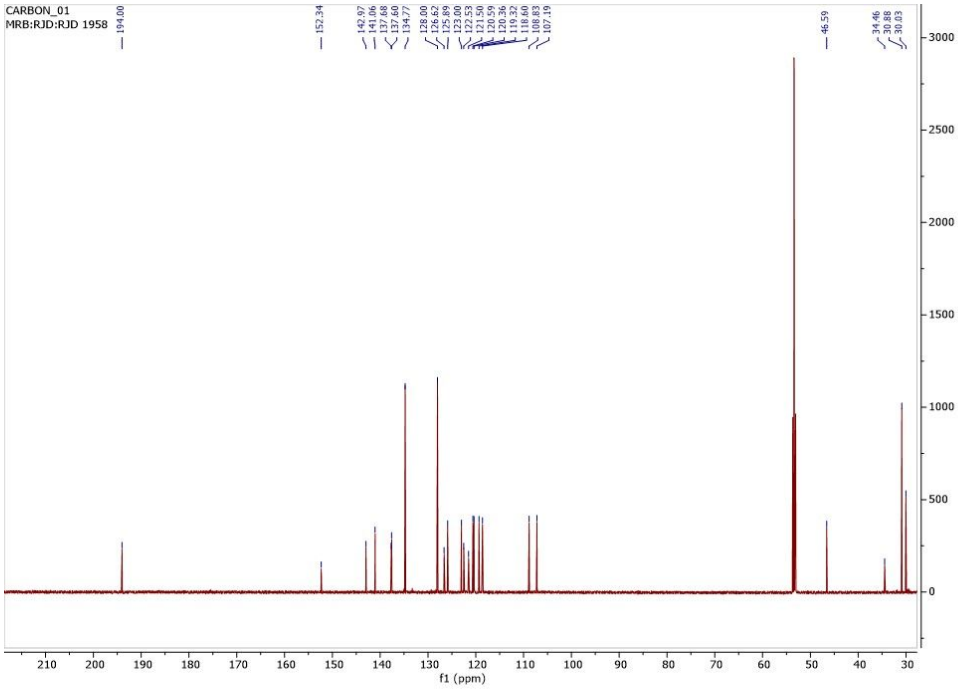

Figure S36. $^{13}C\{^{1}H\}$ NMR spectrum of **3a** recorded in CD_2Cl_2.

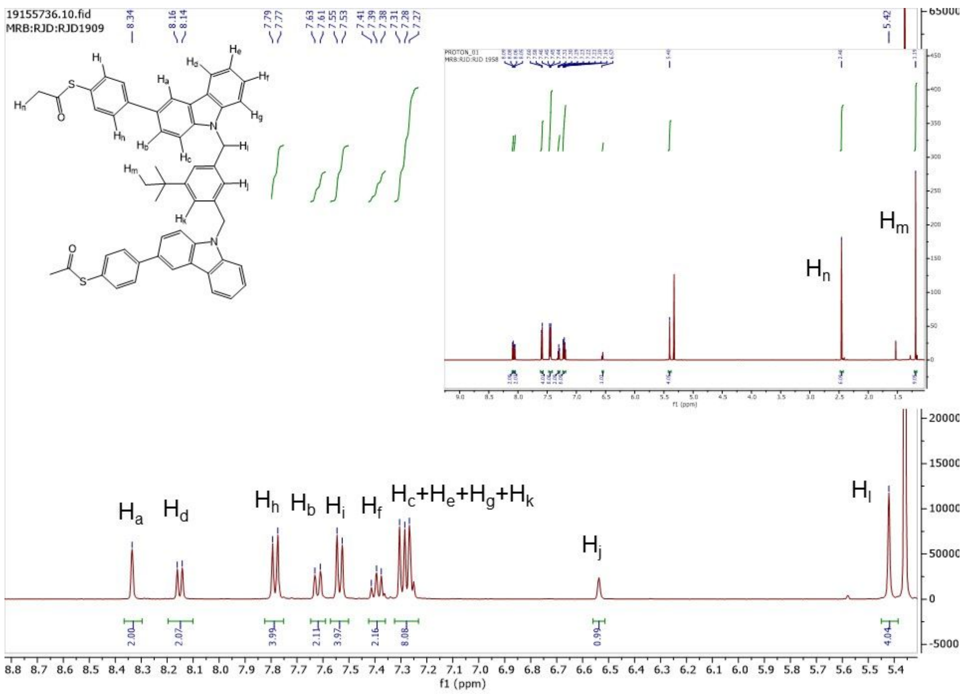

Figure S37. ^{1}H NMR spectra **3b** recorded in CD_2Cl_2.

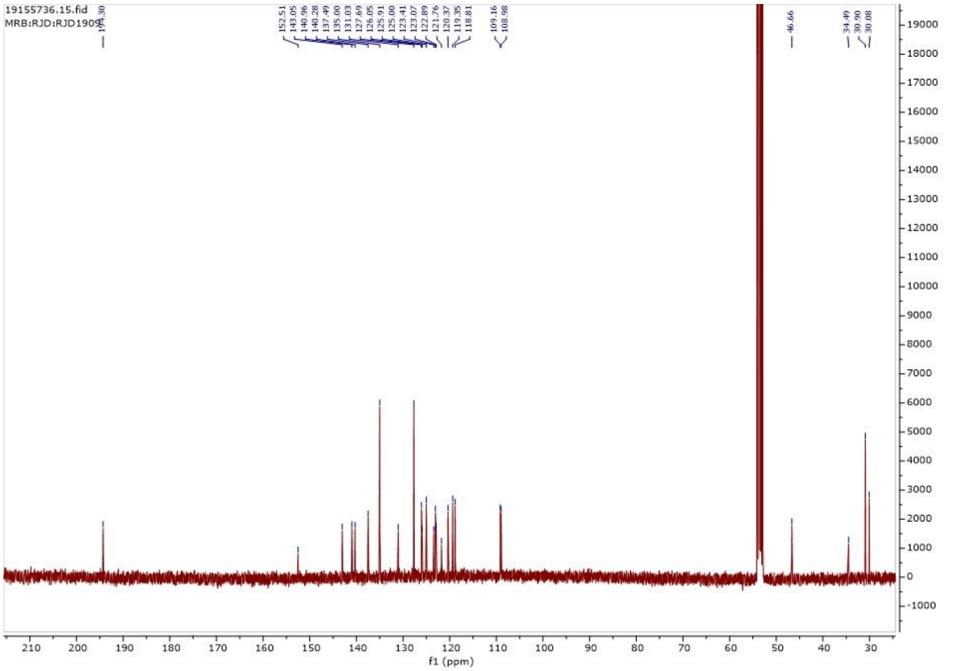

Figure S38. $^{13}C\{^{1}H\}$ NMR spectrum **3b** recorded in CD_2Cl_2.

S3. Crystallographic data

Single-crystal X-ray diffraction experiments (Table S1, Figs. S39–S42) were carried out on a Bruker D8 Venture 3-circle diffractometer, equipped with a PHOTON 100 CMOS area detector, using Mo-Kα radiation (λ = 0.71073 Å) from an Incoatec IµS 3.0 microsource with focusing mirrors. The crystals were cooled using Cryostream 700 open-flow N_2 gas cryostat (Oxford Cryosystems). The data were collected in shutterless mode by narrow frame ω scans covering full sphere of reciprocal space, using APEX3 v.2016.1-0 software, reflection intensities integrated using SAINT v8.38A software (Bruker AXS, 2019). Data were corrected for absorption by semi-empirical method based on Laue equivalents and multiple scans using SADABS 2016/2 software.[4] Structure **1a-TMS** was solved by direct methods (SHELXS 2013/1 program),[5] the rest by dual-space intrinsic phasing (SHELXT 2018/2 program),[6] all were refined by full-matrix least squares using SHELXL 2018/3 software[7] on Olex2 platform.[8] In structure **1a-TMS**, the $C_6H_4S(2)CH_2CH_2Si(2)Me_3$ moiety is disordered equally between two conformations, A and B, the arene ring flipping about the C(36)...C(41) axis by 8°. All methyl groups at Si(1) are disordered by rotation around the Si-C bonds. In **3b-TMS**, the $Me_3Si(1)CH_2CH_2$- moiety is disordered between positions A and B with occupancies 0.85 and 0.15, the $Me_3Si(2)CH_2CH_2S(2)C_6H_4$-moiety is disordered between positions A and B with occupancies 0.819(2) and 0.181(2).

The intramolecular dihedral angle between carbazole planes equals 59.7° in **1a-TMS** and 13.6° in **3b-TMS** molecule; in either case there are no close intramolecular contacts between these moieties. The twist angles between carbazole and adjacent phenylene moieties are 29.2 and 35.3° in **1a-TMS**, 29.4 and 35.2° in **3b-TMS**. The plane of the intra-carbazole bridge is inclined to the two carbazole planes by 86.2 and 83.8° in **1a-TMS**, by 67.7 and 71.5° in **3b-TMS**. In **2a**, the carbazole

and phenylene are almost coplanar (twist angle 5.6°), while the SC(O)Me is nearly perpendicular to the phenylene (twist angle 81.5°) and the propyl group to the carbazole (89.2°).

The packing of carbazole moieties in crystals of **1a-TMS** and **3b-TMS** is shown in Figure S42. In the former, each independent carbazole moiety contacts two other carbazoles in a C-H...π (i.e. edge-to-face) fashion, at interplanar angles of 59.7°. Besides, the inversion-related (hence rigorously parallel) carbazoles containing N(2) atoms, show π...π overlap of their fringes, with shortest C...C and C...N contacts of 3.68 and 3.60 Å, respectively. In **3b-TMS** all carbazole moieties' contacts are of C-H...π kind; the interplanar angles between contacting carbazoles are 60.0, 64.2 and 77.6°.

Table S1. Crystal data and experimental details for **1a-TMS**, **3b-TMS**, **2a**.

	**1a-TMS**	**3b-TMS**	**2a**
CCDC dep.numbers	2388669	2388671	2388670
Empirical formula	$C_{49}H_{54}N_2S_2Si_2$	$C_{58}H_{64}N_2S_2Si_2$	$C_{23}H_{21}NOS$
Formula weight	791.24	1035.49	359.47
T/K	120	120	120
Crystal system	triclinic	orthorhombic	monoclinic
Space group	P-1 (no. 2)	Pbca (no.61)	$P2_1/n$ (no. 14)
a/Å	8.9807(11)	26.123(4)	17.892(2)
b/Å	16.657(2)	8.8033(13)	5.2298(7)
c/Å	17.030(2)	45.243(7)	20.442(3)
α/°	112.410(4)	90	90
β/°	100.638(4)	90	102.529(3)
γ/°	105.175(4)	90	90
Volume/$Å^3$	2154.6(5)	10404(3)	1867.3(4)
Z	2	8	4
$D_{x,}$ g/cm^3	1.220	1.322	1.279
μ/mm^1	0.215	0.198	0.185
Reflections collected	28453	125375	26701
independent	7653	9233	3325
with I≥2σ (I)	4786	5388	1775
Max. 2θ/°	50	50	50
R_{int}	0.070	0.152	0.095
parameters/restraints	553/70	648/21	240/0
Goodness-of-fit on F^2	1.038	1.012	1.012
R_1 [data with I≥2σ (I)]	0.062	0.075	0.062
wR_2 [all data]	0.155	0.186	0.165
$\Delta\rho_{max,min}$/ e $Å^{-3}$	0.41/-0.42	0.40/-0.53	0.22/-0.27

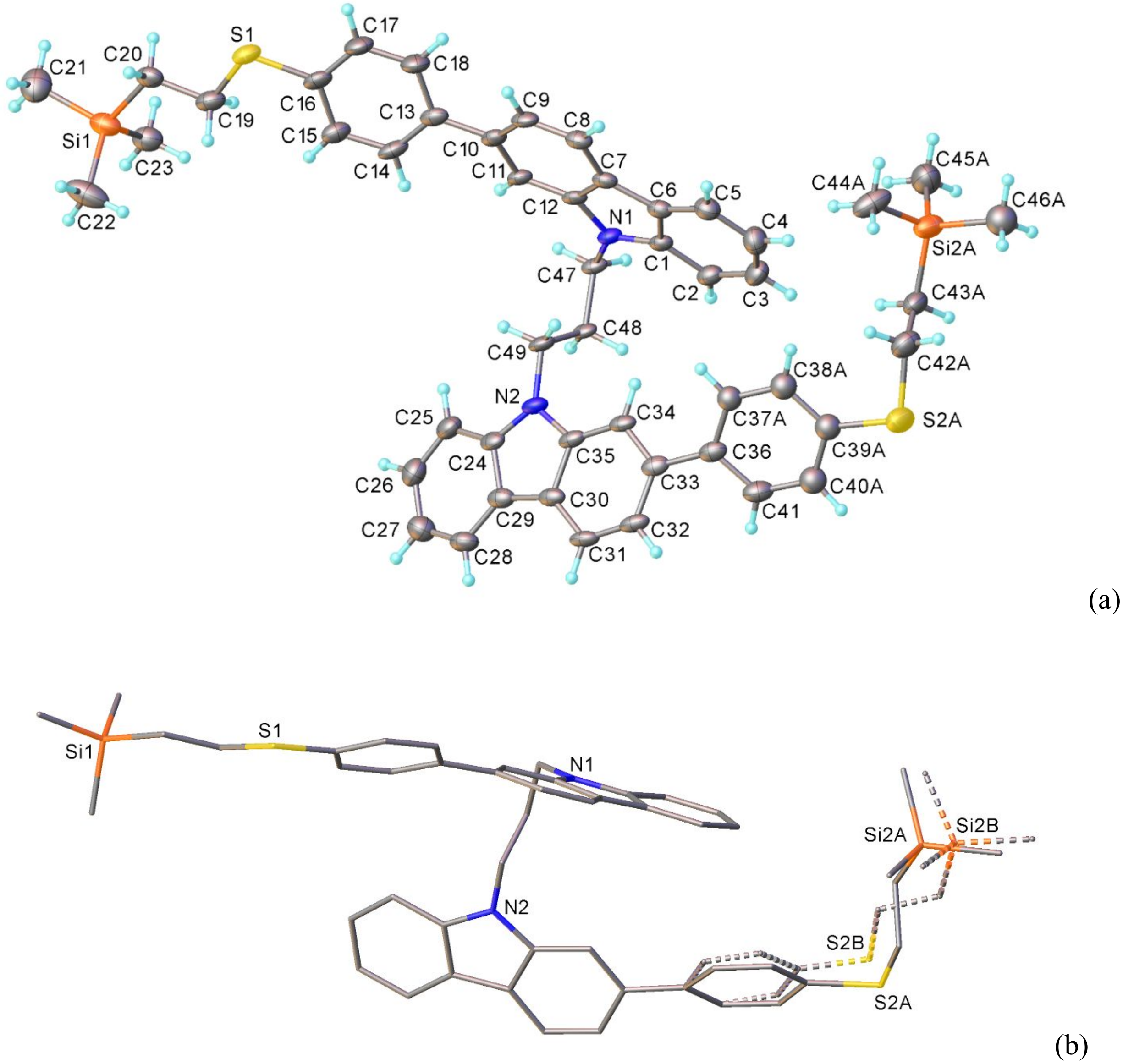

(a)

(b)

Figure S39. X-ray molecular structures: (a) one of the two equiprobable conformers of **1a-TMS**; (b) disorder of **1a-TMS** (H atoms are omitted for clarity).

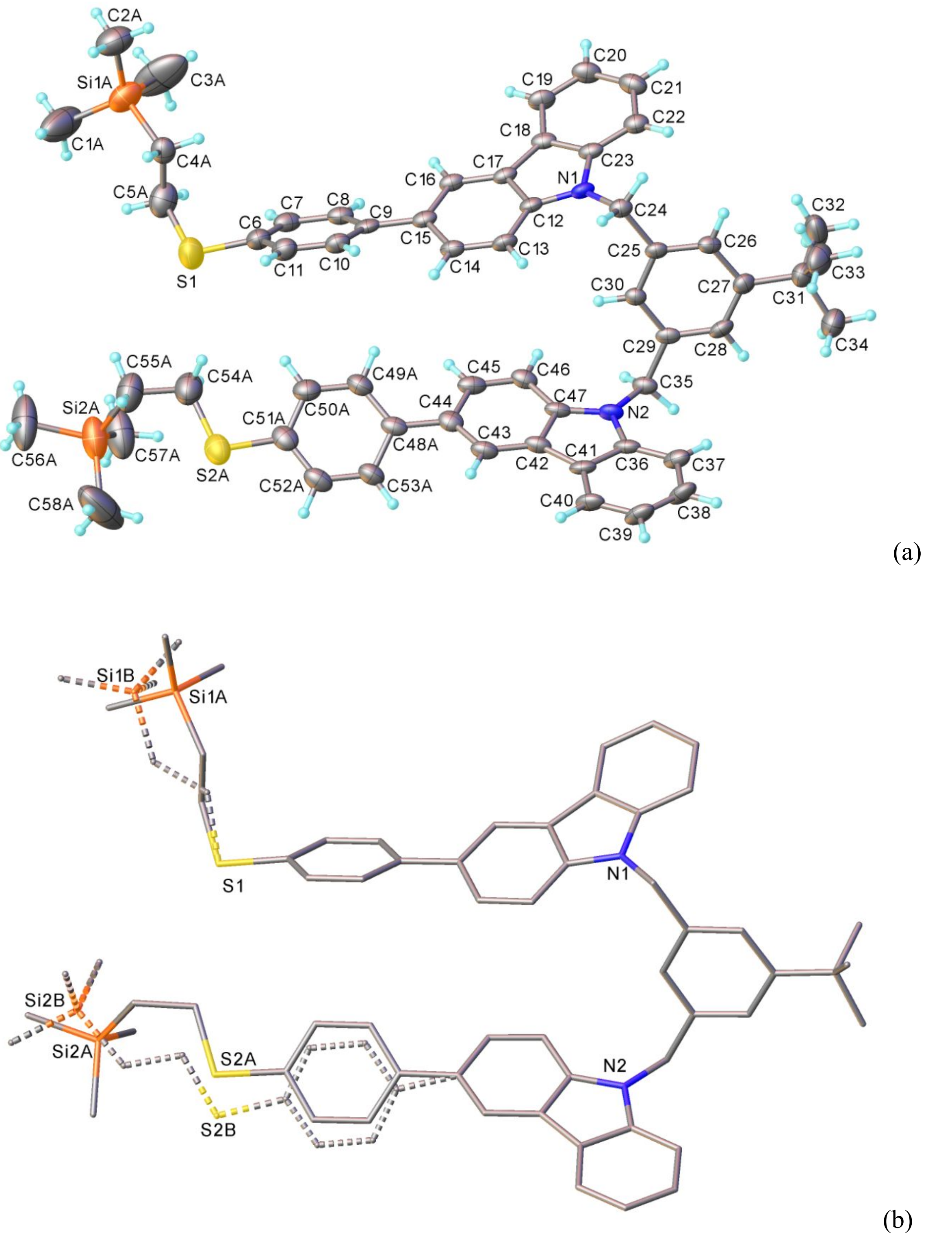

Figure S40. X-ray molecular structures: (a) major conformer (82-85%) of **3b-TMS**; (b) disorder of **3b-TMS** (H atoms are omitted for clarity).

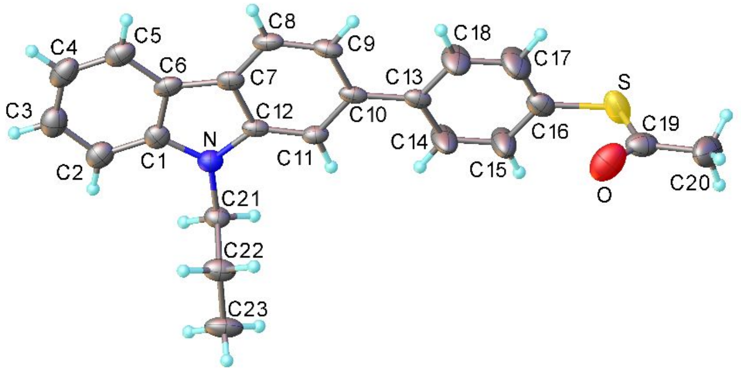

Figure S41. X-ray molecular structures of molecule **2a**. Atomic displacement ellipsoids are drawn at the 50% probability level.

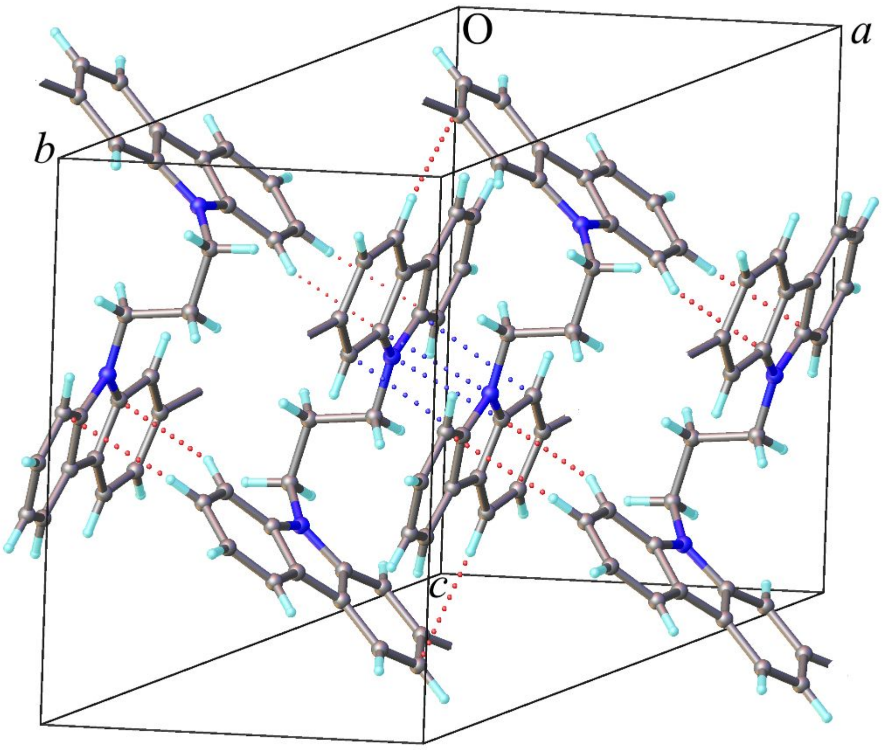

(a)

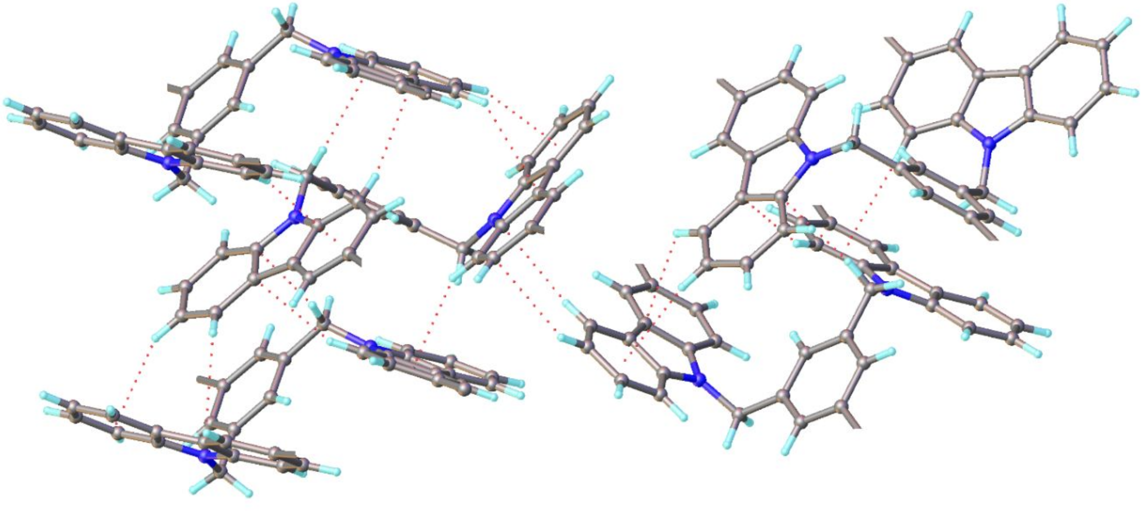

(b)

Figure S42. Crystal packing of carbazole moieties in **1a-TMS** (a) and **3b-TMS** (b). Side-groups are omitted for clarity. Dotted lines indicate close C-H...π (red) and π...π (blue) contacts.

S4. Conductance behavior

All compounds were deposited onto Au(111) samples using the drop casting technique. Au samples were annealed at approximately 900 K for 1-2 minutes, allowed to cool down to room temperature and then introduced into a 1 mM dichloromethane (DCM) solution of the corresponding molecule. After 40 minutes samples were dried off with nitrogen gas to eliminate possible molecular aggregates on the surface. Mechanically cut Au wires (0.25 mm diameter, 99.99% purity, Goodfellow) were used as STM tips. A bias voltage was applied to the sample, using $V_{bias} = 100$ mV and a series resistor of 12 MΩ. The tunnelling current was amplified using a double-stage, home-made, linear current-voltage (I-V)-converter with an overall gain of 2.5×10^{10} V/A (5×10^{8} V/A in the first stage and multiplied by a factor of 50 in the second one).

S4.1. Conductance measurements

To form the molecular junctions, the STM tip was repeatedly indented into the Au sample and then retracted while the current was recorded. When having enough molecules on the surface, there is the possibility to create a molecular junction between the electrodes just after the monoatomic Au-Au contact breaks. This whole process is called a *GZ* curve (tunnelling current *I vs* tip displacement *Z*) and the statistical analysis of the electrical conductance *G* of the molecular junctions (where $G = I/V_{bias}$) is performed collecting thousands of *GZ* curves. 1D conductance histograms were built, considering only the conductance values, as well as 2D conductance vs distance histograms, showing the conductance *vs* the tip displacement for all the curves. In the case of the 2D histograms, the zero tip-sample displacement is set for each *GZ* curve on the monoatomic Au-Au breakage.

The clustering analysis applied was based on the k-means algorithm supported by Matlab.[9-11] To transform the *GZ* curves into valid inputs for the algorithm, a Lorentzian distribution was assigned

to each conductance point and then they were all added up. The number of clusters was initially chosen to be 2 and was then successively increased until the complete conductance distribution was properly fitted, without major overlapping between the conductance clusters.

Figures S43-48 show the 2D G vs displacement histograms of all the conductance plateaus for all respective compounds. On the top right of all panels the apparent stretching length (L_s) of the plateaus is shown. The apparent stretching length (L_s) was obtained in the same way by fitting a Gaussian distribution to each conductance peak and determining the length differences of every trace between the Au-Au monoatomic breakage point (G_0) and $G_m + \sigma$, where G_{m} and σ are the most probable conductance value and the standard deviation of the Gaussian fitting curves, respectively. To take into account multiple junction configurations, Gaussian distributions were fitted to all distances, L_s. The most probable value was then determined as the maximum of the distribution.

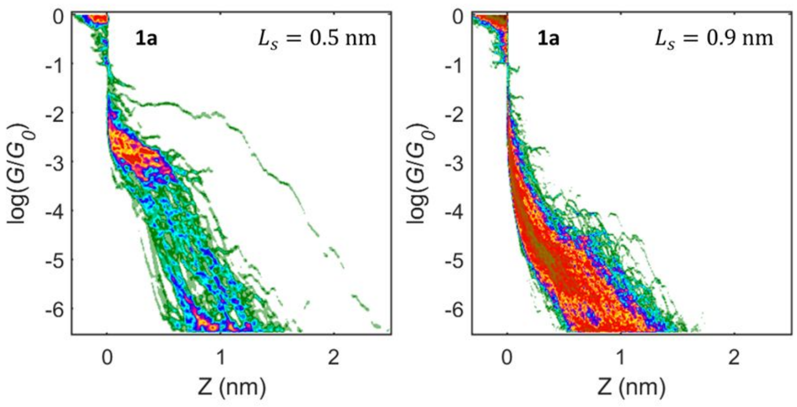

Figure S43. 2D G vs distance histograms and apparent stretching lengths (L_s) of all clusters of compound **1a**.

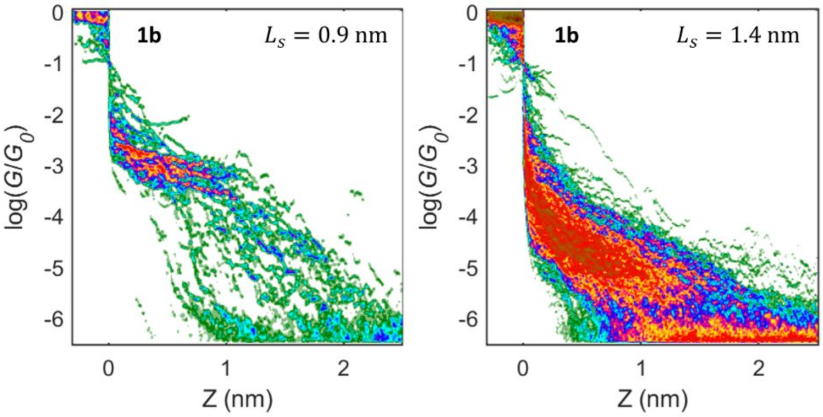

Figure S44. 2D G vs distance histograms and apparent stretching lengths (L_s) of all clusters of compound **1b**.

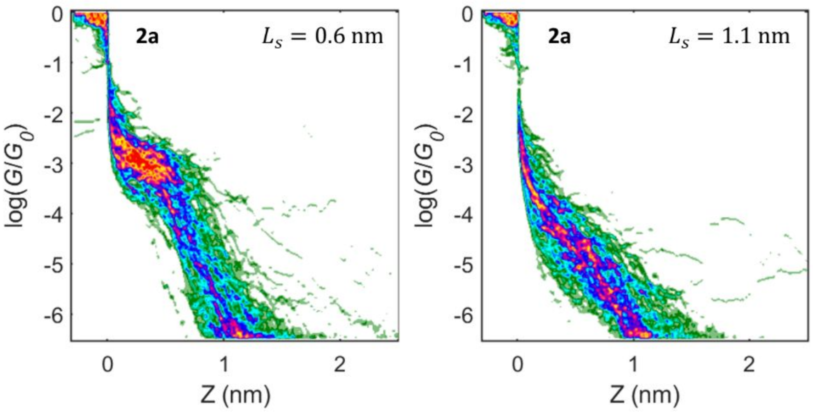

Figure S45. 2D G vs distance histograms and apparent stretching lengths (L_s) of all clusters of compound **2a**.

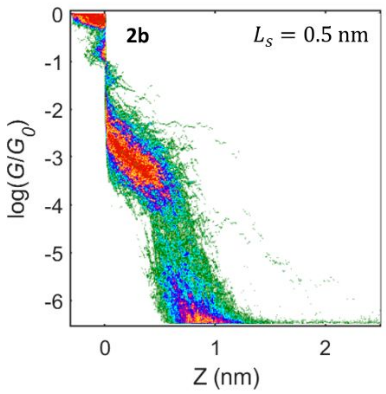

Figure S46. 2D G vs distance histograms and apparent stretching lengths (L_s) of all clusters of compound **2b**.

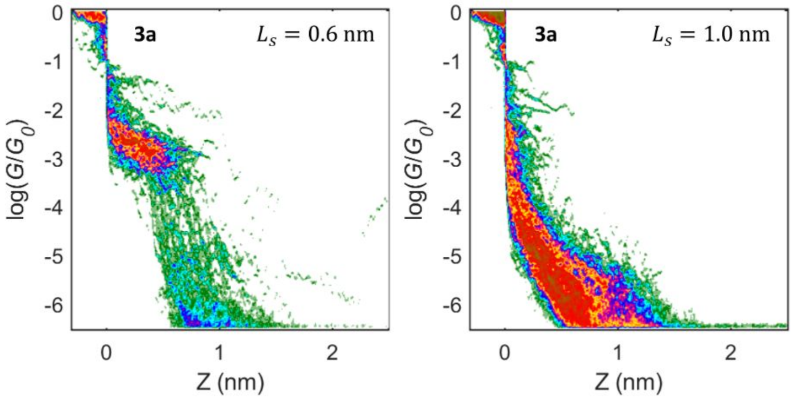

Figure S47. 2D G vs distance histograms and apparent stretching lengths (L_s) of all clusters of compound **3a**.

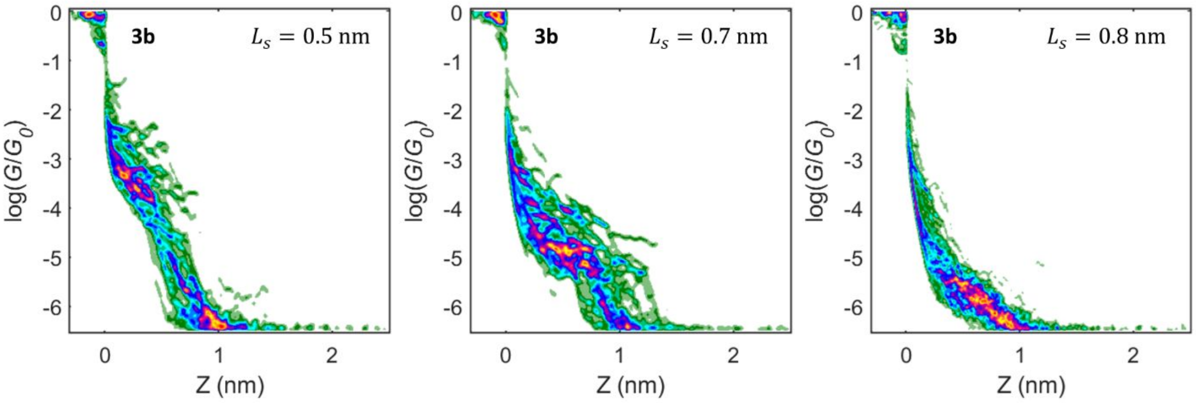

Figure S48. 2D G vs distance histograms and apparent stretching lengths (L_s) of all clusters of compound **3b**.

S4.2. Conductance fluctuation analysis

To analyze the conductance fluctuations, we manually selected the traces with this behavior, obtaining 24.5% for compound **1a**, 5% for compound **1b** and 10% for compound **2a**. From each trace we obtained the points with minimum and maximum in conductance, shown as red and black crosses in Figures S49, S50 and S51. To estimate the variation in *G* produced by this fluctuation, the mean distance between the minimum and maximum points was obtained for each compound and shown in Figures S49 as ΔG. We observed a variation of 1.1 $\log{(G/G_0)}$ for compounds **1a** and **1b** and a variation of 0.8 $\log{(G/G_0)}$ for compound **2a**. This difference in the conductance variations arises from a different effect in the junction, having a fold-unfold process for the molecules **1a** and **1b** and a π-π slide between two molecules for compound **2a**.

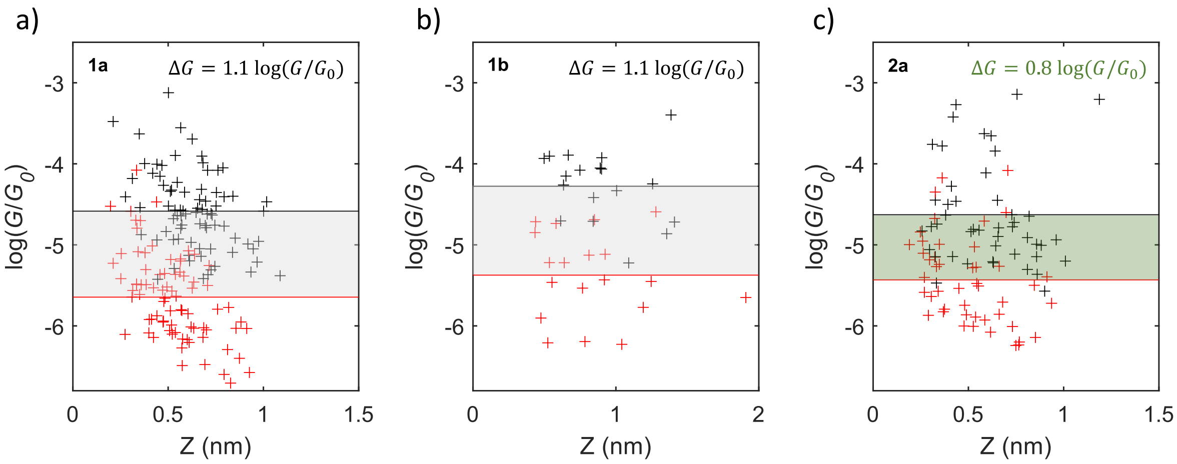

Figure S49. Minimum (red cross) and maximum (black cross) points in the conductance fluctuation for each selected trace for compound **1a**(a) **1b**(b) and **2a**(c). Solid lines represent the mean conductance value for all points (minimum in red and maximum in black). The colored area represents the mean conductance variation for each compound. Conductance variation ΔG between

the mean value of the minimum and maximum of all the points is represented in the top of each panel.

Individual examples of these traces and their minimum and maximum points for each trace are represented in Figure 5a of the main text (compound **1a**) and Figures S50a and S51a (for compounds **1b** and **2a**, respectively). To obtain information about the shape of this conductance fluctuation and 2D histogram of G vs displacement and the mean G behavior is represented in Figure 5b of the main text (compound **1a**) and Figures S50b and S51b (for compounds **1b** and **2a**, respectively). For these representations, the zero-displacement value was set at the distance where the fluctuation begins, meaning the distance at the minimum in conductance obtained previously.

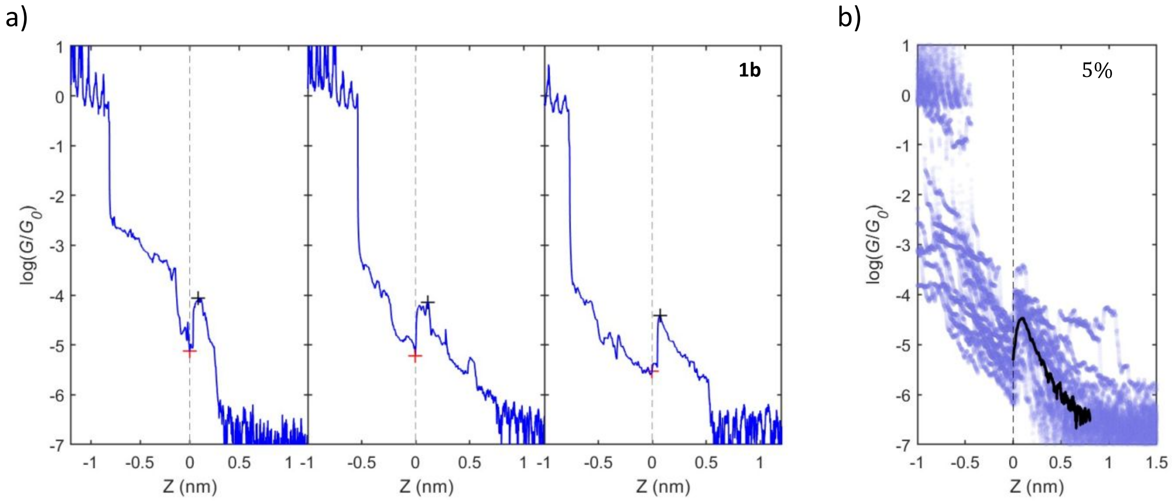

Figure S50. a) Individual GZ traces of compound **1b** exhibiting conductance fluctuations. Red and black crosses show the minimum and maximum G points of the fluctuation, respectively. b) All GZ traces with G fluctuations centered at the minimum G value of the fluctuation of compound **1b**. Percentage of traces with G fluctuations are shown in the top right part of the panel. Black trace on top represents the mean G vs displacement behavior of all the selected traces.

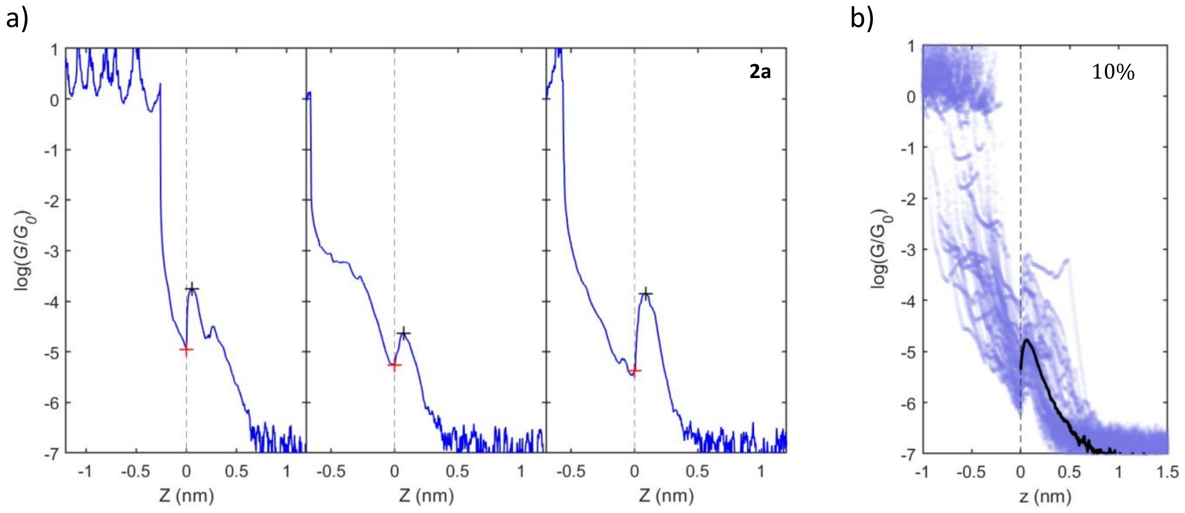

Figure S51. a) Individual *GZ* traces of compound **2a** exhibiting conductance fluctuations. Red and black crosses show the minimum and maximum *G* points of the fluctuation, respectively. b) All *GZ* traces with G fluctuations centered at the minimum *G* value of the fluctuation of compound **2a**. Percentage of traces with *G* fluctuations are shown in the top right part of the panel. Black trace on top represents the mean *G* vs displacement behavior of all the selected traces.

We also compared the *G* fluctuations by comparing the mean normalized *GZ* trace of all the traces containing *G* fluctuations. We represent together the mean *GZ* traces normalizing the conductance and displacement at the origin of the fluctuation. In a similar way, we observe that the *G* fluctuation for compound **2a** is smaller than the ones for compounds **1a** and **1b**. With this analysis we are also able to compare the mean displacement of the fluctuation. For doing so we calculated the distance at which the *G* passes by the origin again (once the *G* increases). Here we observe that the displacement of the fluctuation is smaller for compound **2a** in comparison to compounds **1a** and **1b**. This is a sign of the different origin of the fluctuation for compound **2a**.

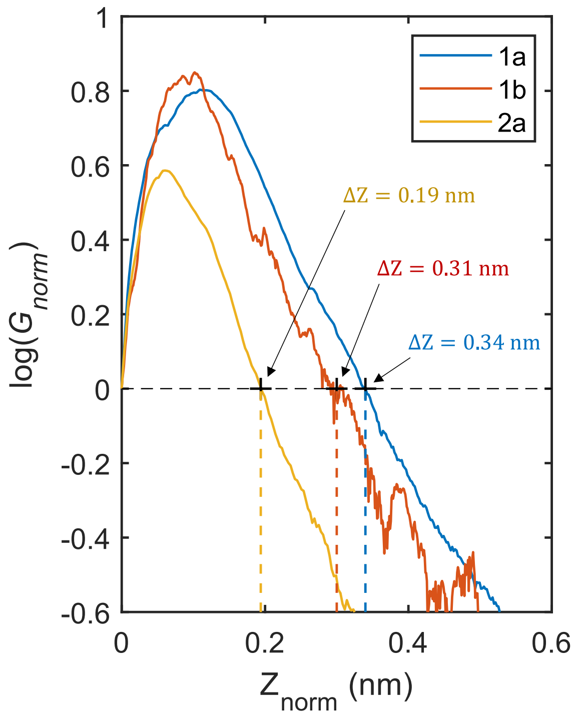

Figure S52. Mean *GZ* traces of the normalized conductance fluctuation for each compound. Blue (**1a**), red (**1b**) and yellow (**2a**). Crosses mark the crossing point of the *G* fluctuation with the origin conductance point. Δ*Z* represents the distance where the *G* crosses the *G* origin again.

S5. XPS measurements

XPS spectra were registered on a Kratos AXIS ultra DLD spectrometer equipped with an Al Kα X-ray monochromatic source (1486.6 eV) and using 20 eV as pass energy. Binding energies were calibrated according to the C1s peak at 284.6 eV.

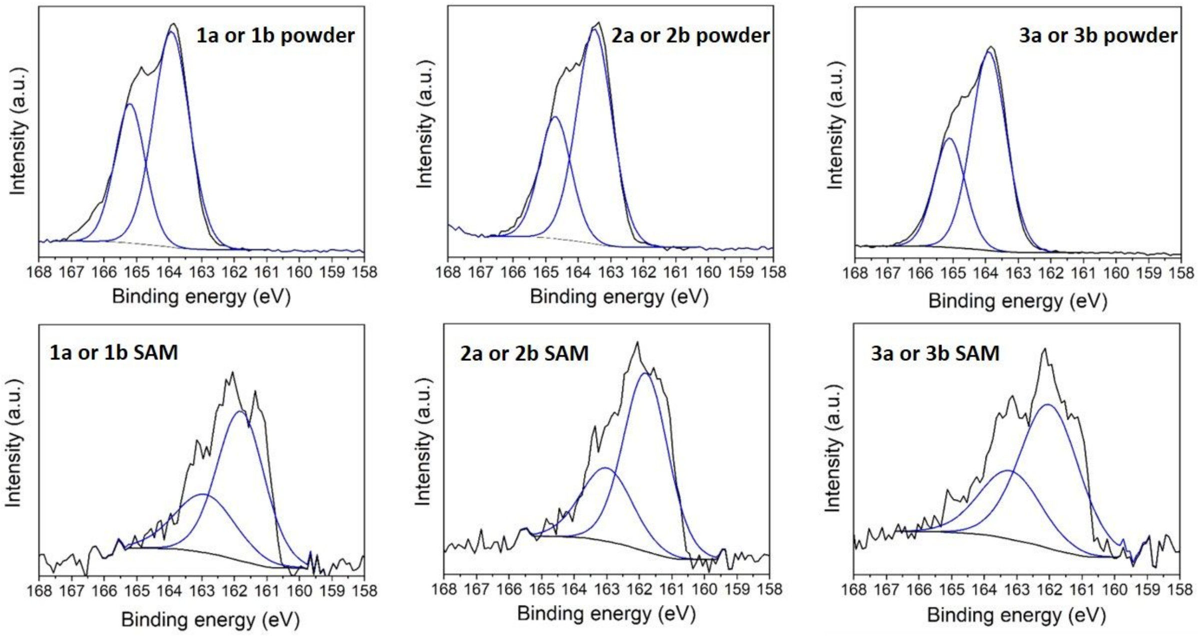

Figure S53. XPS spectra in the S2p region corresponding to **1a**, **1b**, **2a**, **2b**, **3a** and **3b** powders and SAMs. The same XPS spectra were obtained for **1a** and **1b**; **2a** and **2b** and **3a** and **3b** powders, and for their respective SAMs. Therefore, only one spectrum is shown in each panel.

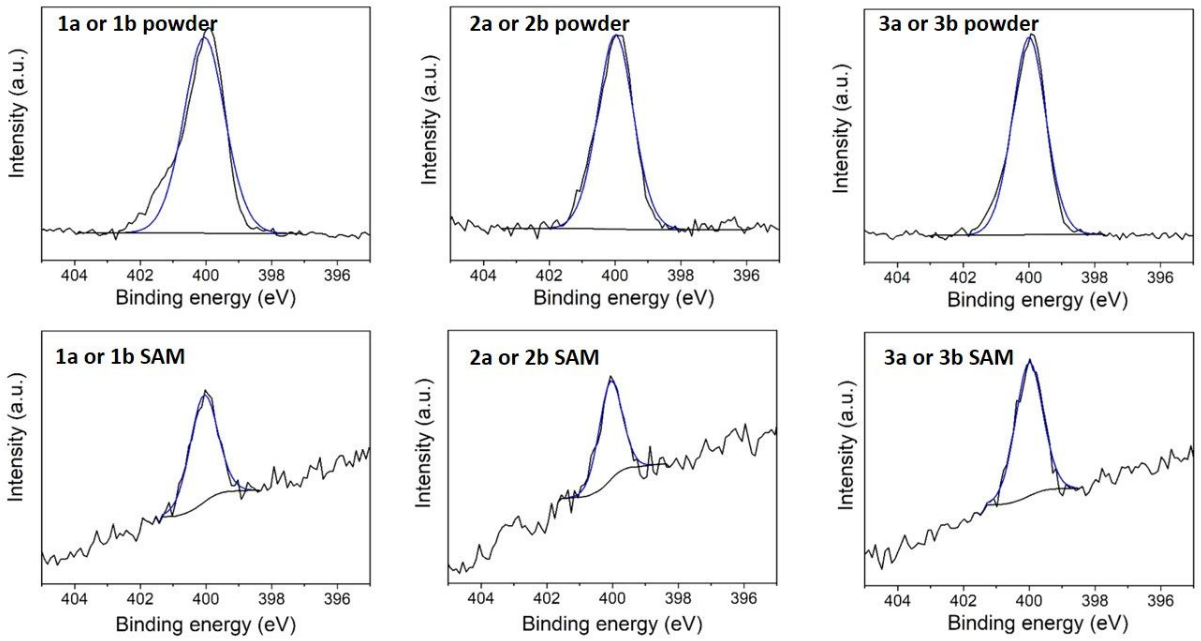

Figure S54. XPS spectra in the N1s region corresponding to **1a**, **1b**, **2a**, **2b**, **3a,** and **3b** powders and SAMs. The same XPS spectra were obtained for **1a** and **1b**; **2a** and **2b;** and **3a** and **3b** powders, and for their respective SAMs. Therefore, only one spectrum is shown in each panel.

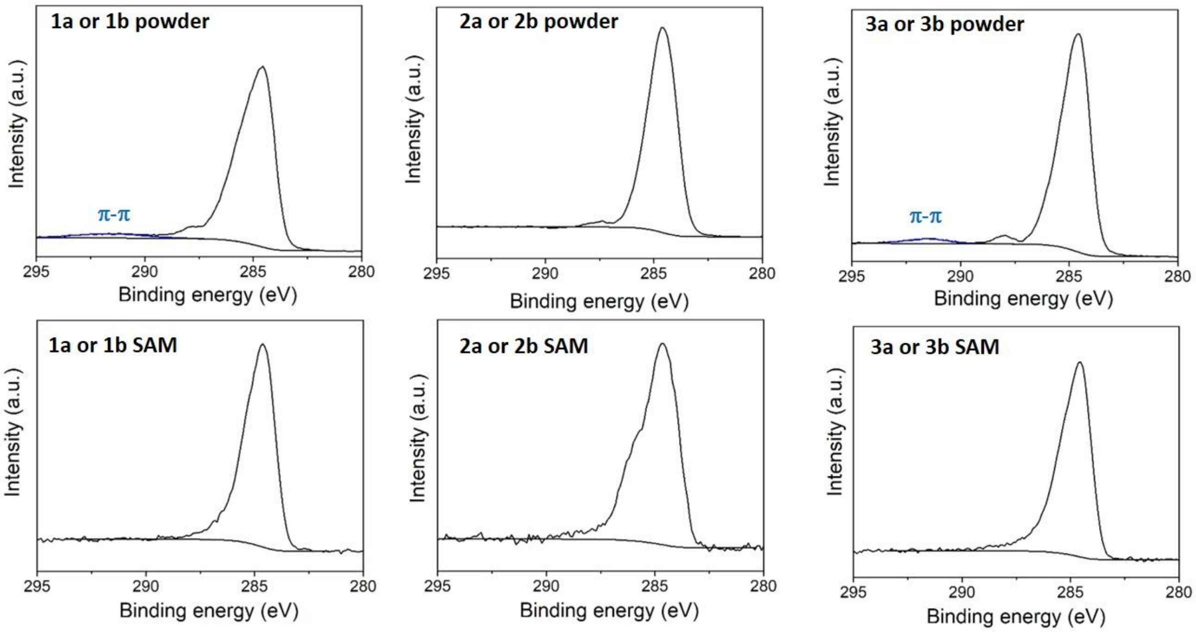

Figure S55. XPS spectra in the C1s region corresponding to **1a**, **1b**, **2a**, **2b**, **3a**, and **3b** powders and SAMs. The same XPS spectra were obtained for **1a** and **1b**; **2a** and **2b** and **3a**; and **3b** powders, and for their respective SAMs. Therefore, only one spectrum is shown in each panel.

b) Theoretical

S6. Density Functional Theory (DFT) modelling of molecular junctions

S6.1 Molecules in the gas phase

The first step in modelling their electronic properties was to calculate the optimum geometries of the molecules. Examination of the structures of the tethered molecules **1a, 1b**, **3a**, and **3b**, led to the identification of four possible geometries, shown in Figures S56, S57, S60, and S61, **(a)** *closed-*asym denotes the fully stacked or ‘hairpin’ geometry where the anchor groups lie on the same side. **(b)** closed-sym denotes the half stacked or the geometry where the anchor groups lie on the opposite side and only the core carbazole unit is stacked. **(c)** open-sym geometry where the thiol anchor groups lie at opposite ends. **(d)** open-asym consists of the non-stacked or ‘open’ configuration. However, examination of the structure of the tethered molecules **2a,** and **2b**, led to the identification of three possible geometries, shown in Figures S58 and S59, **(a)** closed-asym is the fully stacked or ‘hairpin’ geometry, where the anchor groups lie on the same side. **(b)** closed-sym geometry, where the anchor groups lie on the opposite side. **(c)** open is a monomer geometry. These molecules were relaxed using the density functional SIESTA code,[12, 13] to a force tolerance less than 0.01 eV/Å using a double-zeta basis set, and a real- space grid defined with an energy cutoff of 250 Rydberg. The exchange-correlation functional was the van der Waals functional which more accurately describes the longer-range interactions in the stacked junctions.[14, 15]

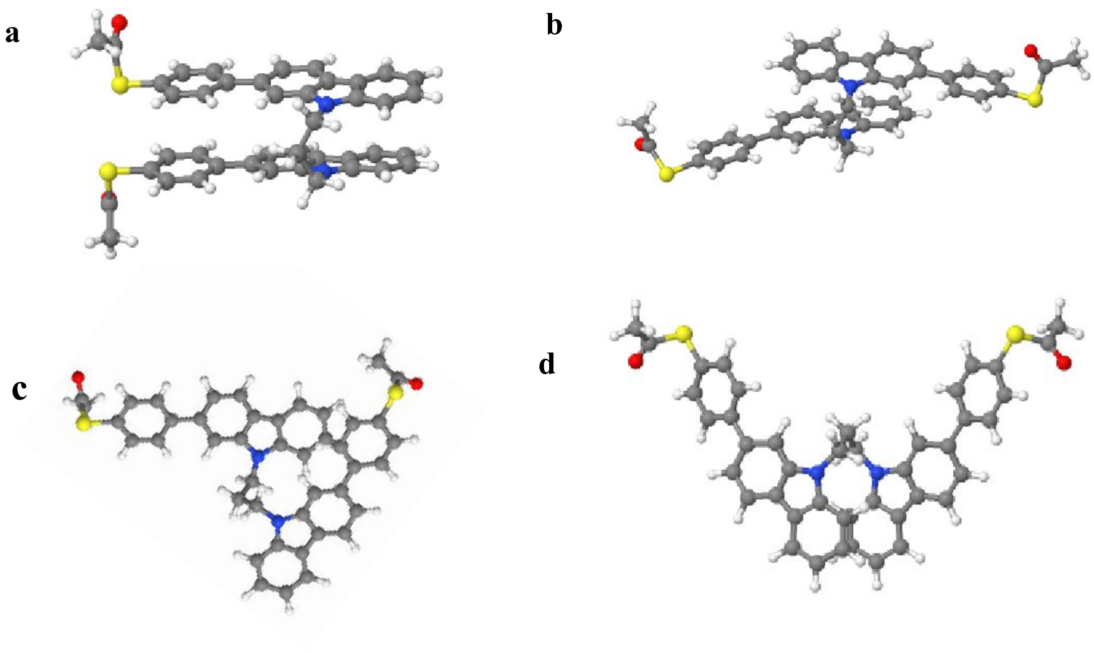

Figure S56. Simulated structures of **1a** for different bridging geometries. **(a)** closed-asym. **(b)** closed-sym. **(c)** open-sym. **(d)** open-asym.

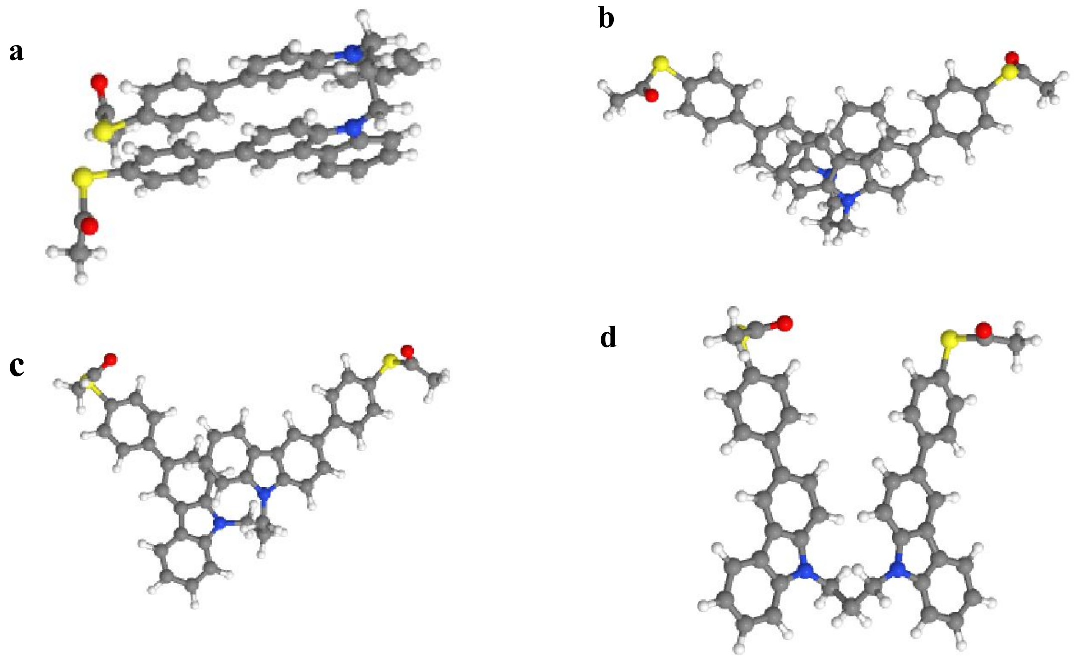

Figure S57. Simulated structures of **1b** for different bridging geometries. **(a)** closed-asym. **(b)** closed-sym. **(c)** open-sym. **(d)** open-asym.

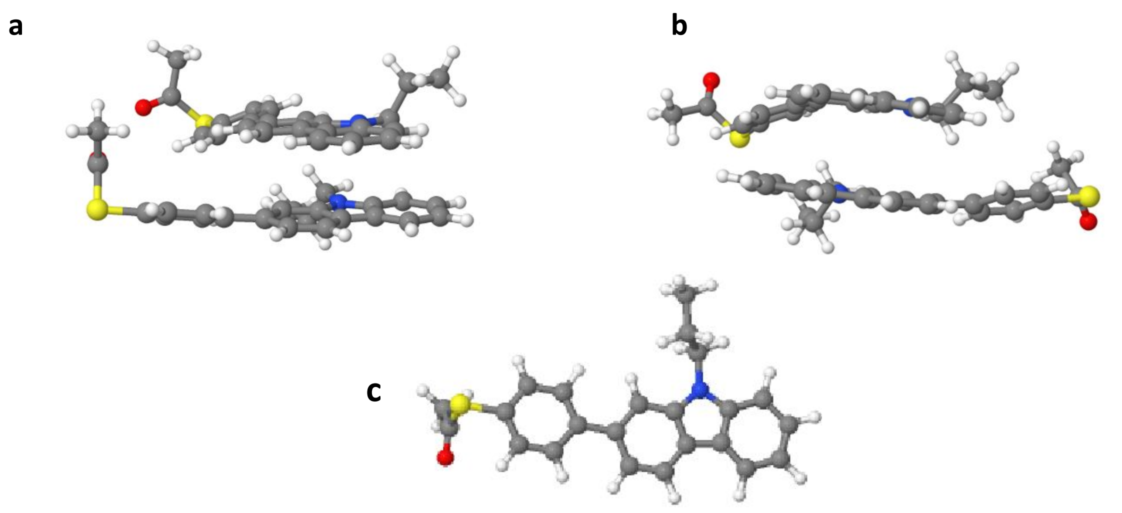

Figure S58. Simulated structures of **2a** for different bridging geometries. **(a)** closed-asym. **(b)** closed-sym. **(c)** open.

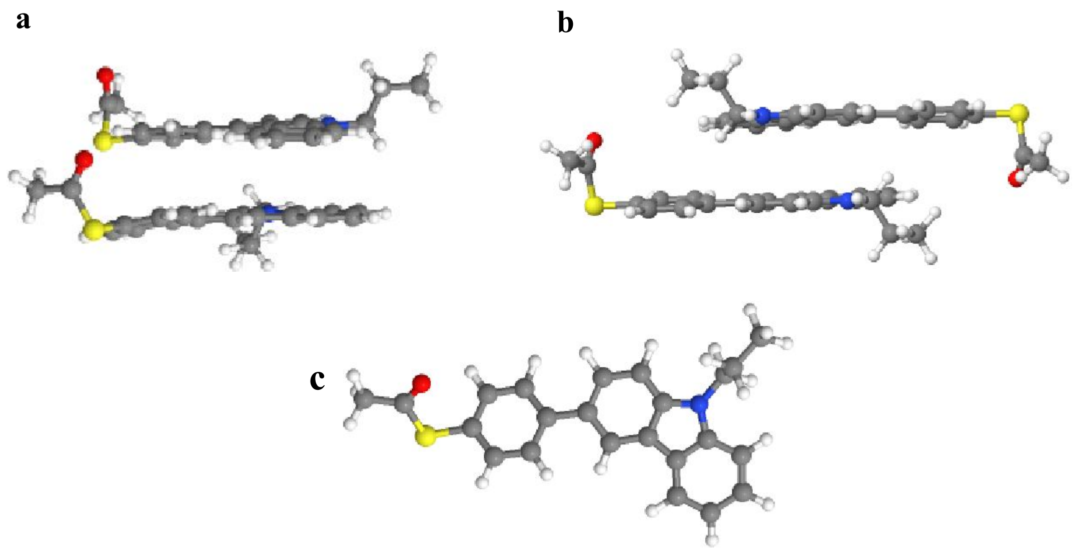

Figure S59. Simulated structures of **2b** for different bridging geometries. **(a)** closed-asym. **(b)** closed-sym. **(c)** open.

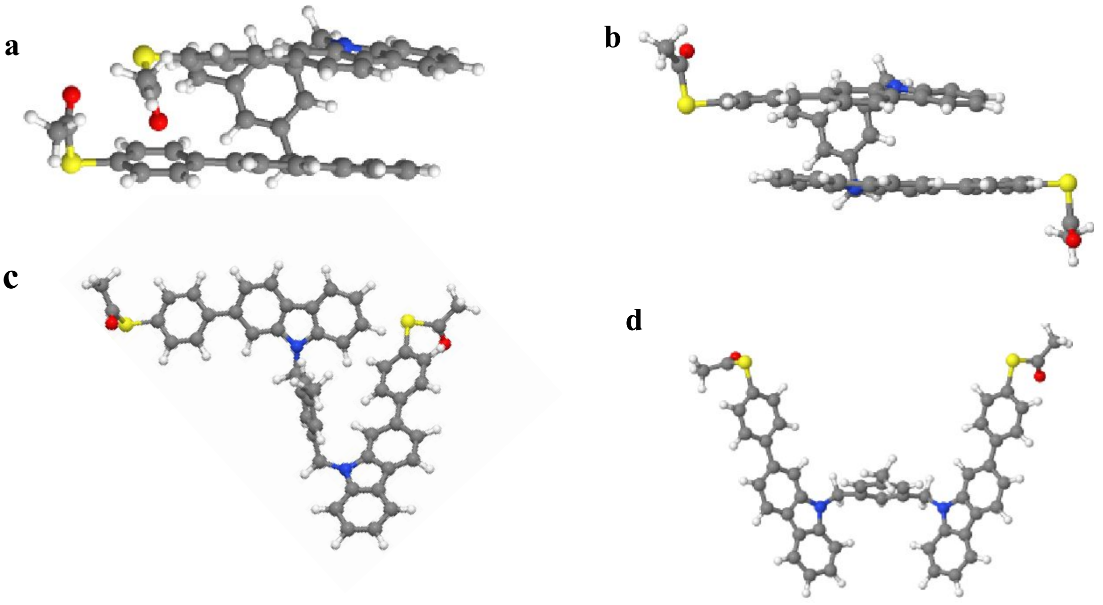

Figure S60. Simulated structures of **3a** for different bridging geometries. **(a)** closed-asym. **(b)** closed-sym. **(c)** open-sym. **(d)** open-asym.

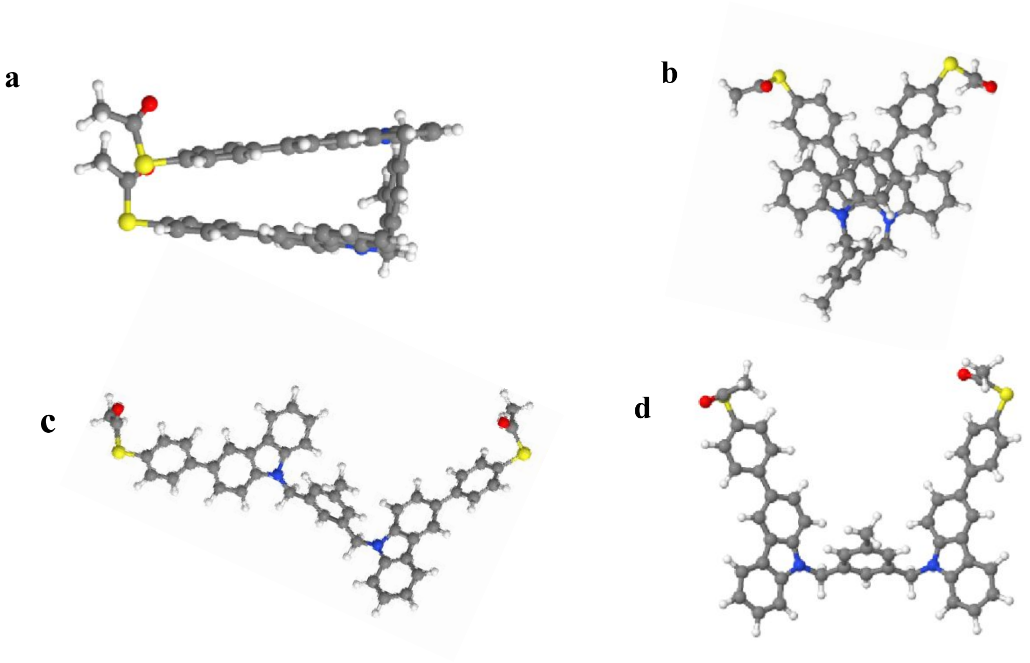

Figure S61. Simulated structures of **3b** for different bridging geometries. **(a)** closed-asym. **(b)** closed-sym. **(c)** open-sym. **(d)** open-asym.

S6.2 Binding energies to gold electrodes

The next step was to understand how the studied molecules attach to the surface of the gold electrodes, which are modelled as a pyramid of gold atoms.[16, 17] For these molecules, the expected binding is through the tip of the gold coupling to the terminal sulfur atom in each molecule. The binding distance d is defined as the distance between the gold – sulfur atoms. The ground state energy of the total structure (E_{AB}^{AB}) was calculated using SIESTA, with the parameters defined as those in this section. In SIESTA, the energy of the individual molecule in the presence of the fixed basis well-defined as $E_{A}^{AB}$and for the electrode is E_{B}^{AB}. The binding energy was calculated using the following equation:[18]

$$\Delta E(AB) = E_{AB}^{AB} - E_{A}^{AB} - E_{B}^{AB}$$

The optimum binding distance d between Au-H is 2.5 Å, and the optimum binding energy is approximately 0.03 eV. The optimum binding distance d between Au-S is 2.4 Å, and the optimum binding energy is approximately 0.94 eV. The optimum binding distance d between Au-C is 2.4 Å, and the optimum binding energy is approximately 1 eV.

S6.2.1 Binding energy of molecules on Au-H, (B.E1)

After obtaining the optimum structures of the molecules, shown in Figs. S62-S64, the next step was to find the optimum distance between the Au electrode and the molecules.

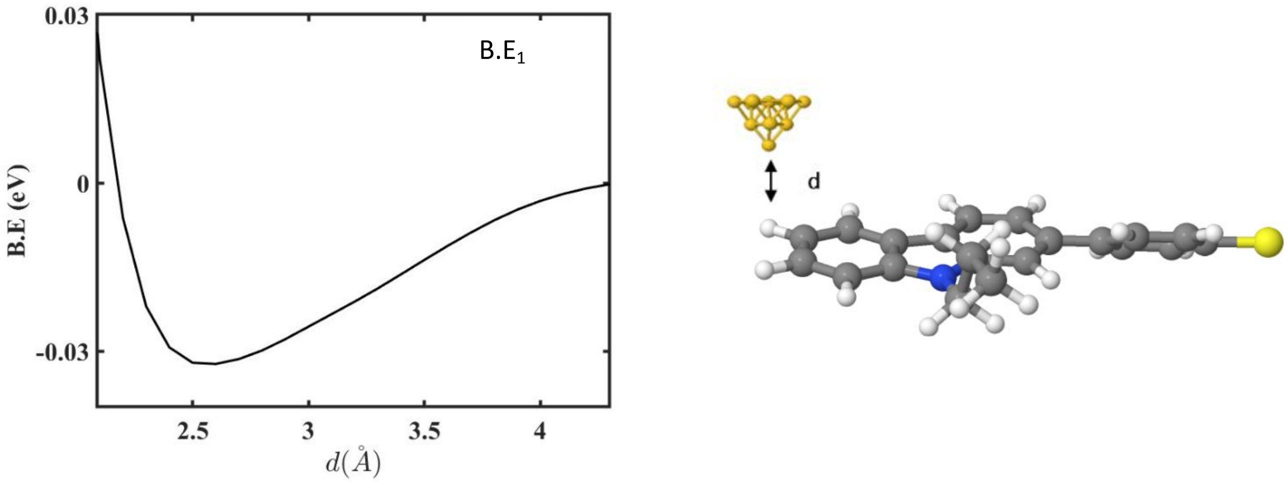

Figure S62. **Left panel**: Binding energy as a function of the optimum binding distance *d*, where *d* is found to be approximately 2.5 Å, and binding energy B. E= 0.03 eV. **Right panel**: B.E_1 represents **2a** binding to Au.

S6.2.2 Binding energy of molecules on Au-S,(B.E2)

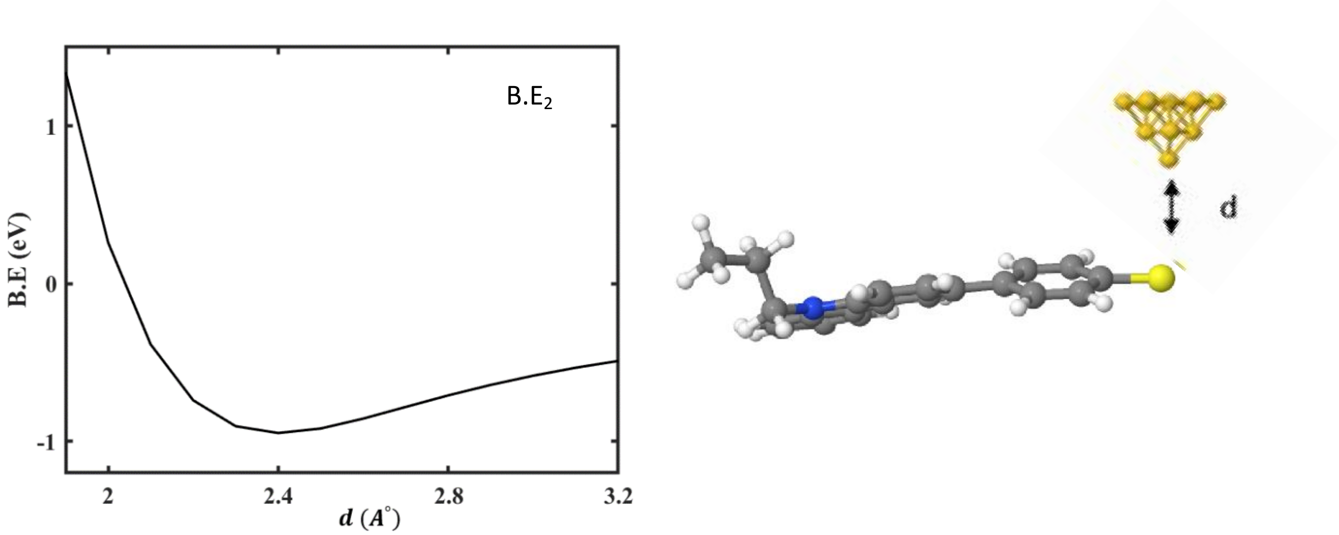

Figure S63. **Left panel**: Binding energy as a function of the optimum binding distance *d*, where *d* is found to be approximately 2.4 Å, and binding energy B. E= 0.94 eV. **Right panel**: B.E_2 represents **2a** binding to Au.

S6.2.3 Binding energy of molecules on Au-C,(B.E3)

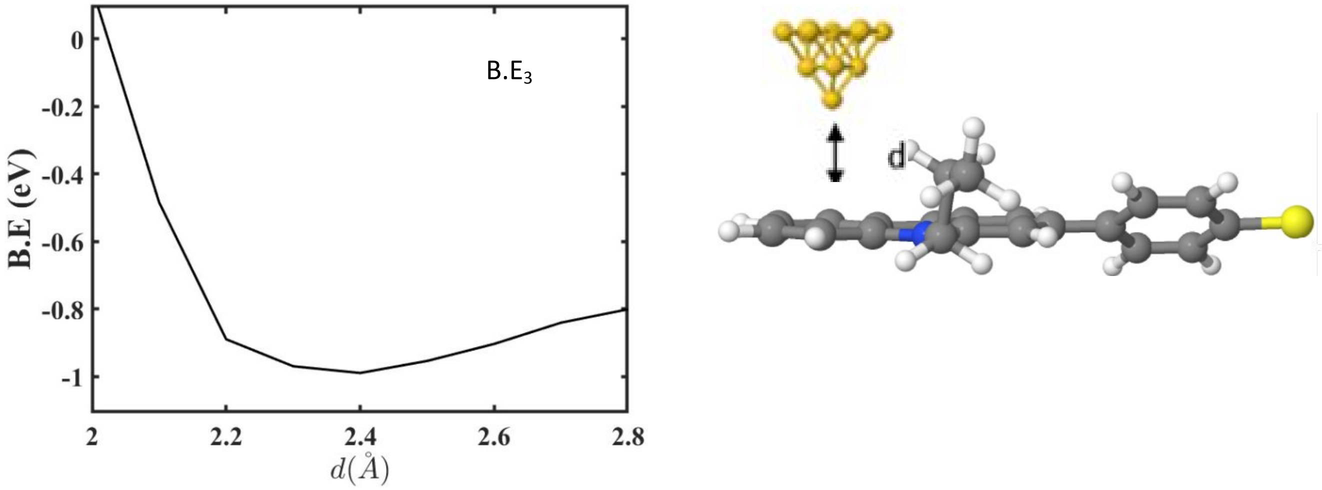

Figure S64. **Left panel**: Binding energy as a function of the optimum binding distance *d*, where *d* is found to be approximately 2.4 Å, and binding energy B. E= 1 eV. **Right panel**: B.E_3 represents **2a** binding to Au.

Table S2: Summary of the binding energy calculations.

Compound	$\boldsymbol{B.E\ (eV)}$	$\boldsymbol{d(A^{\circ})}$
$B.E_1$	0.03	2.5
$B.E_2$	0.94	2.4
$B.E_3$	1	2.4

S6.3 Metal|Molecule|Metal junctions.

Molecular junctions were formed from electrodes consisting of 5 layers of (111) gold each containing 25 atoms terminated by a pyramid of 11 gold atoms. SIESTA was then used to generate a Hamiltonian using the parameters described previously (here a GGA (generalized gradient approximation) functional was used to minimize computational expense as the transport properties are not strongly dependent on the functional).[19, 20] The zero-bias transmission coefficient $T(E)$ was calculated using this Hamiltonian, via the GOLLUM[21] code. The room temperature conductance can then be evaluated from the Landauer formula,

$$G = \frac{2e^2}{h}\int_{-\infty}^{\infty} dE\, T(E)\left(\frac{df(E)}{dE}\right)$$

where $f(E)$ is the Fermi-Dirac distribution, e is the electronic charge and h is Planck's constant. In this study all the Fermi levels of the transmission coefficient T(E) were 0.5 eV.

In the following sections, conductance values are presented for all the different junctions.

S6.3.1 Transmission coefficients

S6.3.1.1. Results for 1a

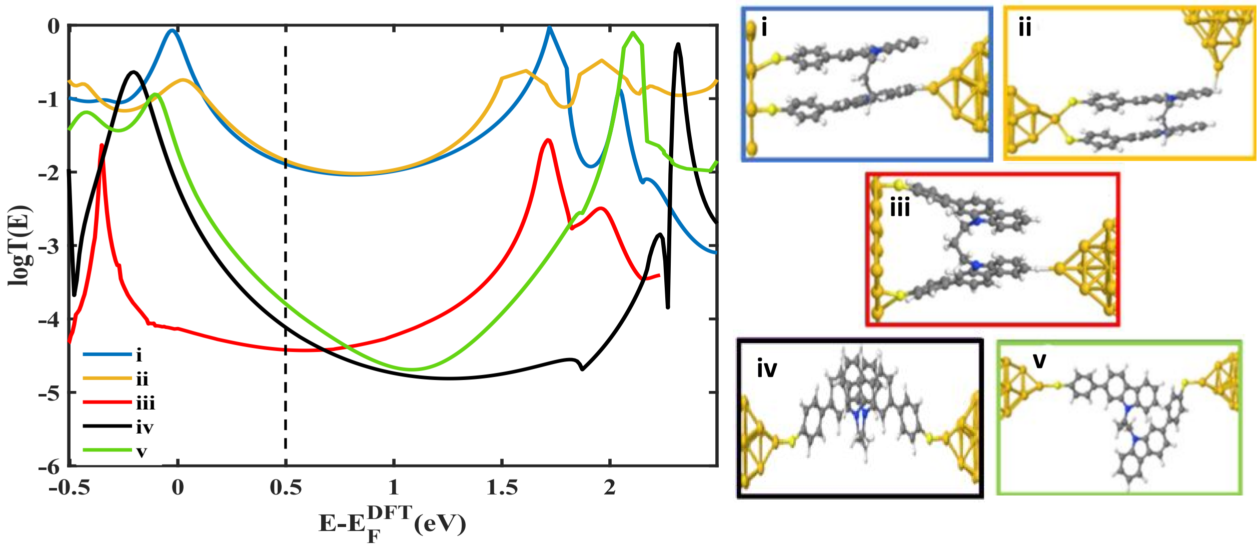

Figure S65. The transmission coefficient of **1a** for different bridging geometries. **(i,ii)** Closed-asym. **(iii)** Open-asym. **(iv)** closed-sym. **(v)** Open-sym. $T(E)$ values are taken at $E - E_F^{DFT} = 0.5$ eV.

The entire set of configurations resulted in two distinct conductance values. The first geometry (**i**, blue), for two thiol anchor groups attached to the gold on one side, and the terminal hydrogen atom on the carbazole attached to the gold as another anchor group. The second geometry (**ii**, gold) is for two thiol anchor groups attached to the gold on one side, through the π-system (co-facial) of

the terminal hydrogen atom on the carbazole attached to the gold as another anchor group. These two junctions show the highest transmission value. Closed asymmetric junctions lead to increased conductance. The third geometry (**iii**, red) is open-asym, in which two thiol anchor groups attached to the gold on one side, and the terminal hydrogen atom on the carbazole attached to the gold as another anchor group. The fourth geometry (**iv**, black) is half stacked, where the sulfur atoms are at the opposite end, while the last geometry (**v**, green) for open-sym. The last three junctions led to low conductance.

S6.3.1.2. Results for 1b

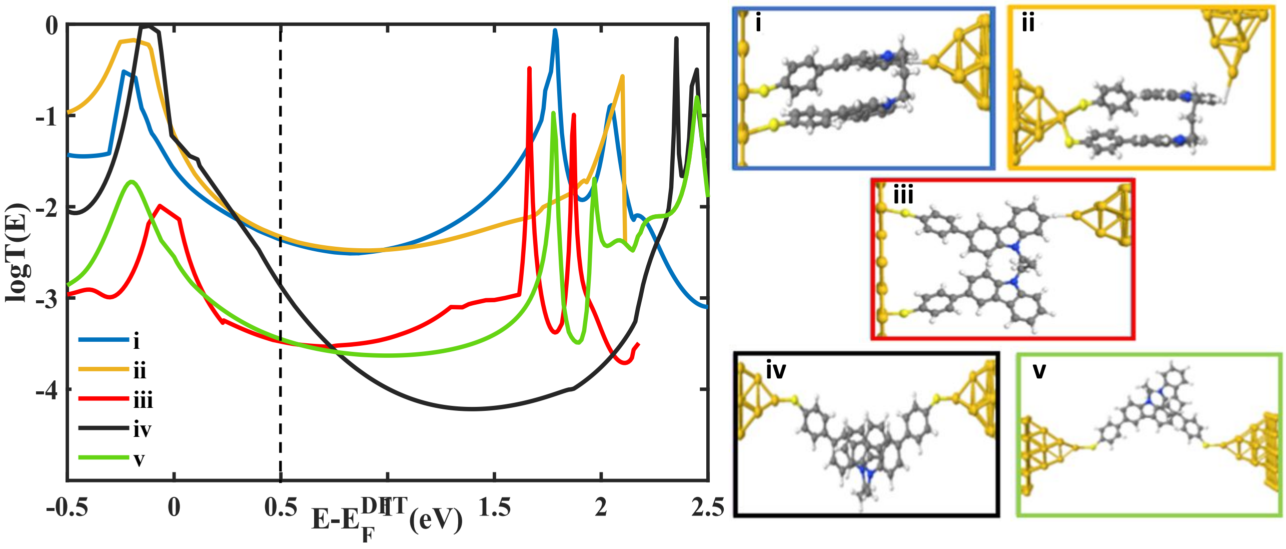

Figure S66. The transmission coefficient of **1b** for different bridging geometries. **(i,ii)** closed-asym. **(iii)** open-asym **(iv)** closed-sym. **(v)** open-sym. $\mathrm{T(E)}$ values are taken at $\mathrm{E - E_F^{DFT} = 0.5\ eV}$.

Again, the entire set of configurations resulted in two distinct conductance values. The first geometry (**i**, blue) is for two thiol anchor group attached to the gold on one side, and the terminal

hydrogen atom on the carbazole attached to the gold as another anchor group. The second geometry (**ii**, gold) is for two thiol anchor group attached to the gold on one side, and the hairpin geometry binding through the π-system (co-facial) of the terminal hydrogen atom on the carbazole attached to the gold as another anchor group. These two junctions represent the highest transmission values. Closed asymmetric junctions lead to increased conductance. The third geometry (**iii**, red), for open-asym, where two thiol anchor group attached to the gold on one side, and the terminal hydrogen atom on the attached to the gold as another anchor group while the fourth geometry (**iv**, black), is half stacked, where the sulfur atoms are at the opposite end. The last geometry (**v**, green) for open-sym. The last three junctions led to low conductance.

S6.3.1.3. Results for 2a

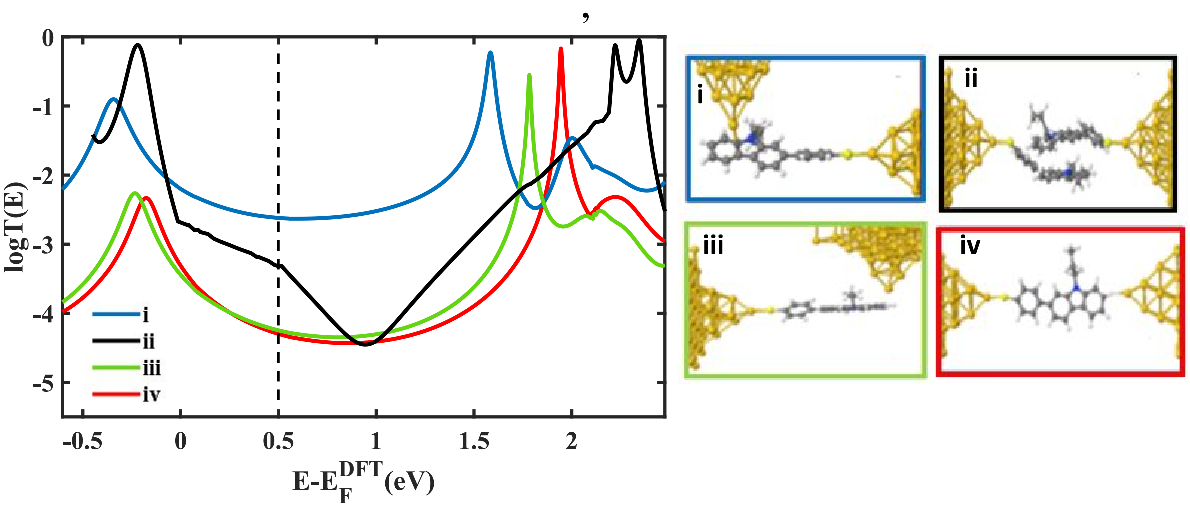

Figure S67. The transmission coefficient T(E) of different bridging geometries for the **2a**. **(i, iii, iv)** open-sym, **(ii)** closed-sym. T(E) values are taken at $E - E_F^{DFT} = 0.5$ eV .

The first geometry (**i** blue) involves co-facial binding through the central core of the carbazole molecule gives the highest value of $T(E)$. Therefore, for the single molecule junction, where the second electrode is weakly coupled, the value of the transmission is controlled by the coupling strength between electrode and the pi-system of the molecule. The second geometry (ii, black) is half stacked, where the sulfur atoms are at the opposite end, is shown with a low T(E) value. The third geometry (**iii,** green) considers the co-facial binding connecting the terminal phenyl ring to one electrode, as the thiol is connected to the second. The fourth configuration (**iv,** red) is for one thiol anchor group attached to the gold on one side, and the terminal hydrogen atom on the carbazole attached to the gold as another anchor group. The last two junctions show the lowest transmission values.

S6.3.1.4. Results for 2b

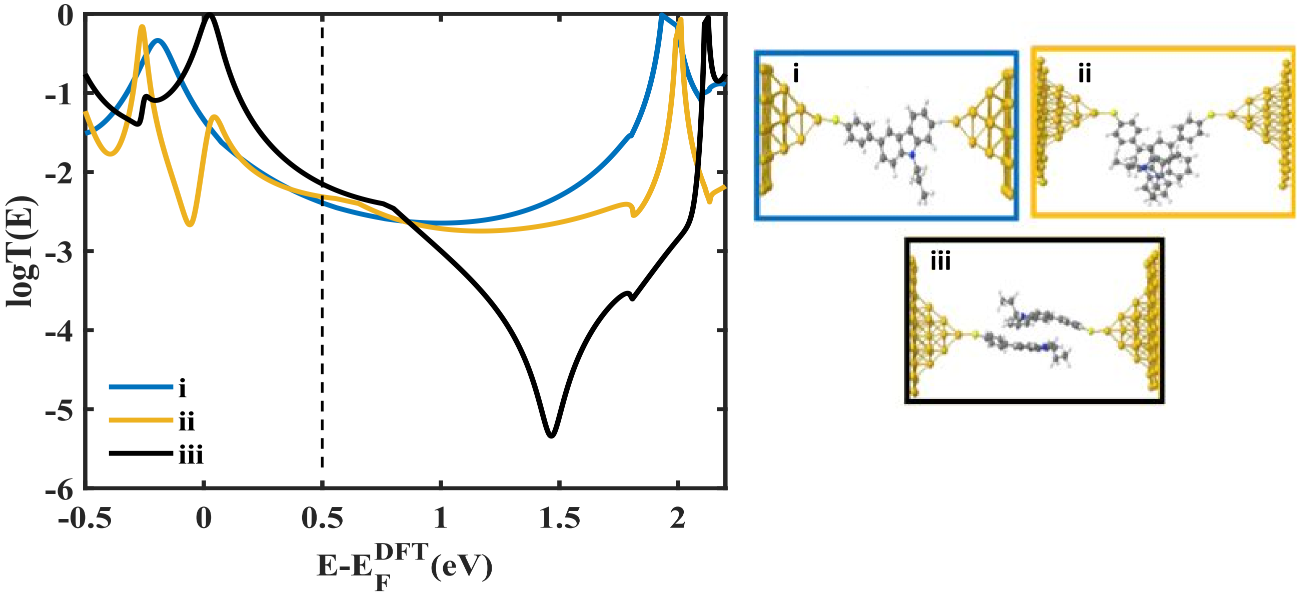

Figure S68. The transmission coefficient of **2b** for different bridging geometries. **(i)** Open. **(ii)** Closed-asym. **(iii)** Closed sym . T(E) values are taken at $E - E_F^{DFT} = 0.5$ eV .

The first configuration (**i**, blue) is for one thiol anchor group attached to the gold on one side, and the terminal hydrogen atom on the carbazole attached to the gold as another anchor group. The second geometry (**ii**, gold) is the fully stacked, where the sulfur atoms are at the same end. The last geometry (**iii**, black) is half stacked, where the sulfur atoms are at the opposite end. All junctions have almost the same conductance values at E_F=0.5 eV.

S6.3.1.5. Results for 3a

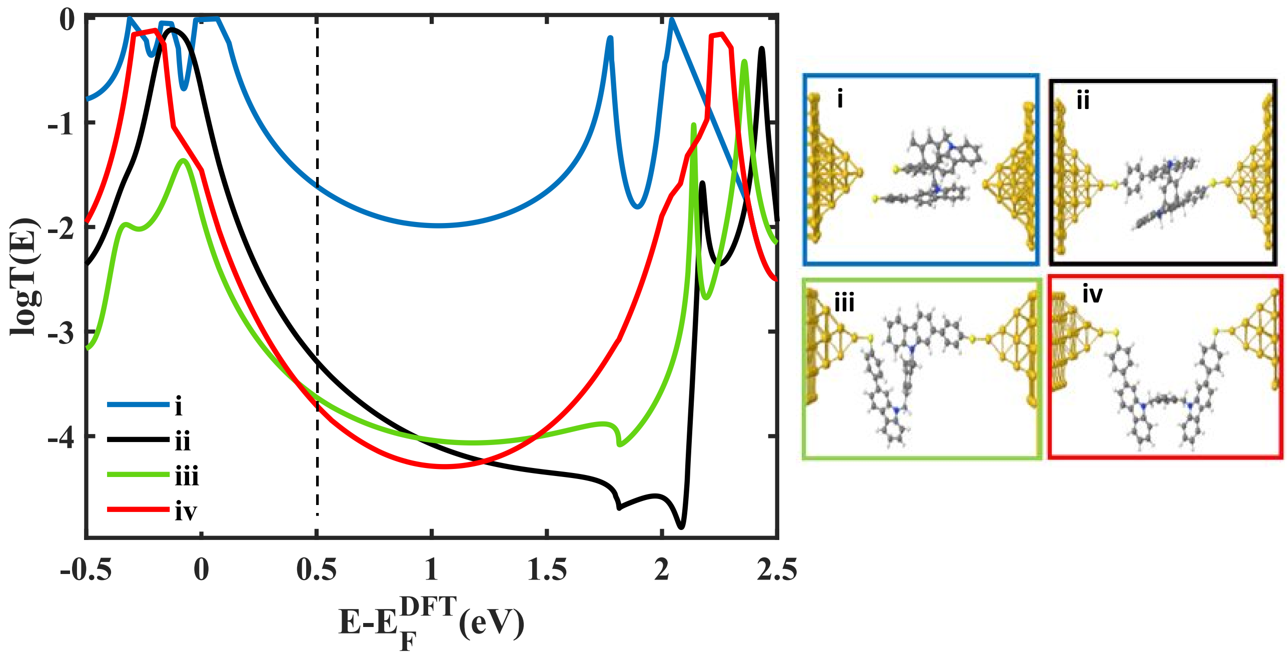

Figure S69. The transmission coefficient of **3a** for different bridging geometries. **(i)** Closed-asym. **(ii)** Closed- sym. **(iii)** Open-sym. **(iv)** Open-asym. $\mathrm{T(E)}$ values are taken at $\mathrm{E - E_F^{DFT} = 0.5\ eV}$.

The first structure (**i**, blue) is the fully stacked or hairpin, where the two sulfur atoms are at the same end and the two terminal hydrogens bind to the second electrode shows the highest

conductance. The second case (**ii**, black) is half stacked, where the sulfur atoms are at the opposite end. The third geometry (**iii**, green) is open-sym while the last geometry (**iv**, red) for open-asym. The last three junctions show the lowest transmission values.

S6.3.1.6. Results for 3b

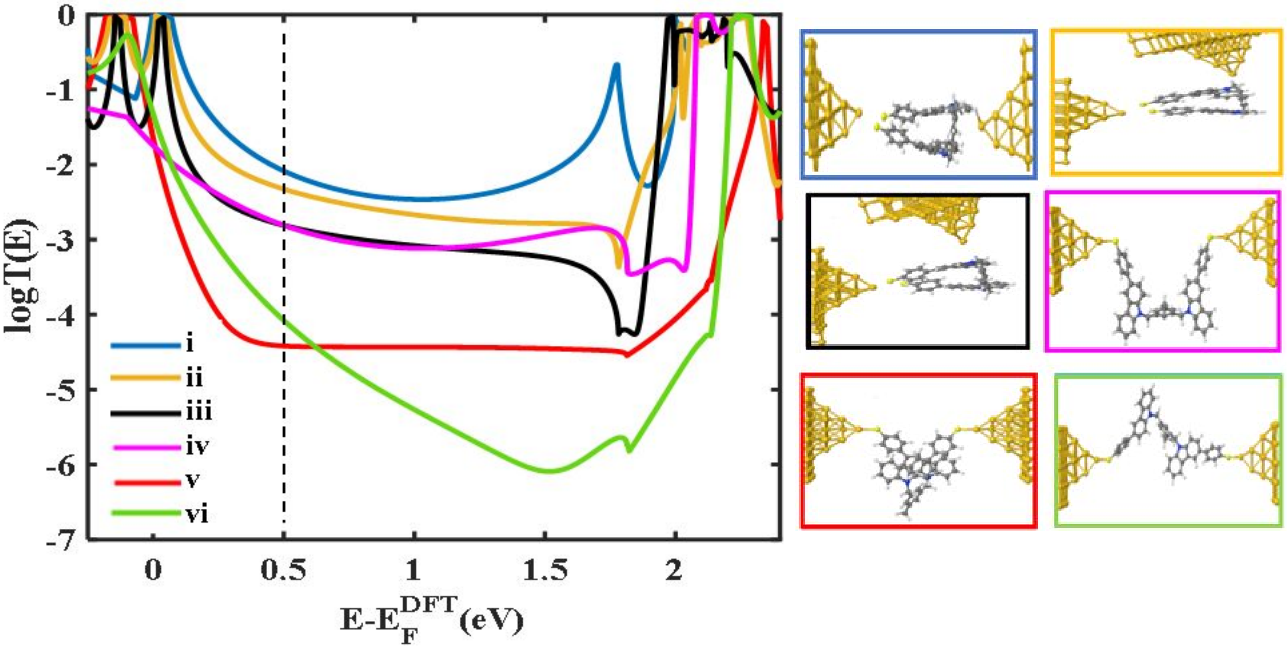

Figure S70: The transmission coefficient of **3b** for different bridging geometries. **(i,ii,iii)** Closed-asym. **(iv)** Open-asym. **(v)** Closed- sym. **(vi)** Open- sym . $T(E)$ values are taken at $E - E_F^{DFT} = 0.5$ eV .

The first structure (**i**, blue) is the fully stacked or hairpin, where the two sulfurs are at the same end and the two terminal hydrogens bind to the second electrode, showing the highest transmission value. The hairpin geometry binding through the π-system (co-facial) of the terminal hydrogen (**ii**, gold) shows a high transmission value, as it happened in (**i**, blue). The hairpin geometry binding through the pi-system (co-facial) of the terminal phenyl ring (**iii**, black) shows the medium transmission value. The fourth geometry (**iv** purple) is open-asym and shows the medium

transmission value as it happened in (**iii**, black). The fifth geometry (**v**, red) is half-stacked, where the sulfur atoms are at the same end, shows a lower transmission value. The last geometry (**vi**, green) is open-sym where two thiol anchor groups attached to the gold on two sides show the lowest transmission value.

S7. Comparison between experiment and theory

The experimental measurements of **1a** show two conductance values which can be identified as corresponding to different bridging geometries that **1a** could form. Theoretically, the high conductance value occurs for a closed-asym, the lowest value occurs for the closed-sym, open-sym, and open-asym shown in Figure S65. The experimental measurements of **1b** show two conductance values and can be identified with the different bridging geometries that **1b** could form. Theoretically, the high conductance value occurs for a closed-asym, the lowest value occurs for the closed-sym, open-sym, and open-asym shown in Figure S66. The experimental measurements of **2a** show two conductance values and can be identified with different bridging geometries that **2a** could form. Theoretically, the conductance values occur as shown in Figure S67. The experimental measurements of **2b** show one conductance value and can be identified different bridging geometries that **2b** could form. Theoretically, the conductance value occurs with all junctions shown in Figure S68. The experimental measurements of **3a** show two conductance values and can be identified with different bridging geometries that **3a** could form. Theoretically, the high conductance value occurs for a closed-asym, the lowest value occurs for the closed-sym, open-sym, and open-asym shown in Figure S69. The experimental measurements of **3b** show three conductance values and can be identified with different bridging geometries that **3b** could form. Theoretically, the conductance value occurs as shown in the Figure S70.

Table S3. Comparison between experimental and theory

**1a**						
**G-Level**	High			Low		
**Geometries**	Closed-asym			Open-asym	Closed-sym	Open-sym
**Junction**	I	ii		iii	iv	v
**Theory**	-1.9	-1.9		-4.4	-4.1	-3.8
**Theory-Avg.**	-1.9			-4.1		
**Exp-Avg.**	-2.9			-5.4		
**1b**						
**G-Level**	High			Low		
**Geometries**	Closed-asym			Open-asym	Closed-sym	Open-sym
**Junction**	i	ii		iii	iv	v
**Theory**	-2.3	-2.3		-3.5	-2.9	-3.5
**Theory-Avg.**	-2.3			-3.3		
**Exp-Avg.**	-3.0			-4.8		
**2a**						
**G-Level**	High			Low		
**Geometries**	open-sym			Closed-sym	Open-sym	
**Junction**	i			ii	iii	iv
**Theory**	-2.6			-3.3	-4.3	-4.3
**Theory-Avg.**	-2.6			-4.0		
**Exp-Avg.**	-3.0			-5.0		
**2b**						
**G-Level**	High			Low		
**Geometries**	Open	Closed-asym	Closed-sym			
**Junction**	i	ii	iii			
**Theory**	-2.4	-2.3	-2.2			
**Theory-Avg.**	-2.3					
**Exp-Avg.**	-3.1					

Table S3 continued. Comparison between experimental and theory

<table>
<tr><th colspan="7">3a</th></tr>
<tr><td>G-Level</td><td colspan="2">High</td><td colspan="2" rowspan="6"></td><td colspan="3">Low</td></tr>
<tr><td>Geometries</td><td colspan="2">Closed-asym</td><td>Closed-sym</td><td>Open-sym</td><td>Open-asym</td></tr>
<tr><td>Junction</td><td colspan="2">i</td><td>ii</td><td>iii</td><td>iv</td></tr>
<tr><td>Theory</td><td colspan="2">-1.6</td><td>-3.3</td><td>-3.6</td><td>-3.7</td></tr>
<tr><td>Theory-Avg.</td><td colspan="2">-1.6</td><td colspan="3">-3.5</td></tr>
<tr><td>Exp-Avg.</td><td colspan="2">-2.8</td><td colspan="3">-5.8</td></tr>
<tr><th colspan="7">3b</th></tr>
<tr><td>G-Level</td><td colspan="2">High</td><td colspan="2">Medium</td><td colspan="2">Low</td></tr>
<tr><td>Geometries</td><td>Closed-asym</td><td>Closed-asym</td><td>Closed-asym</td><td>Open-asym</td><td>Closed-sym</td><td>Open-sym</td></tr>
<tr><td>Junction</td><td>i</td><td>iii</td><td>iv</td><td>v</td><td>ii</td><td>vi</td></tr>
<tr><td>Theory</td><td>-2.1</td><td>-2.3</td><td>-2.8</td><td>-2.8</td><td>-4.4</td><td>-4.1</td></tr>
<tr><td>Theory-Avg.</td><td colspan="2">-2.2</td><td colspan="2">-2.8</td><td colspan="2">-4.3</td></tr>
<tr><td>Exp-Avg.</td><td colspan="2">-3.1</td><td colspan="2">-4.0</td><td colspan="2">-5.2</td></tr>
</table>

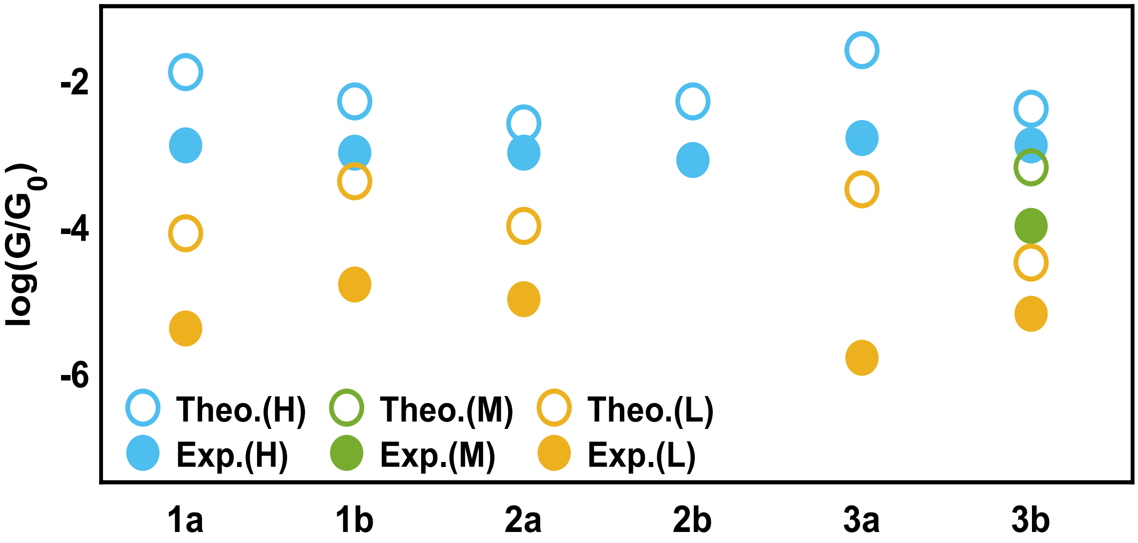

Figure S71. The transmission coefficient $log\left({}^{G}/_{G_0}\right)$, for different geometries. Configurations **1a**, **1b**, **2a**, and **3a** exhibit two distinct levels of conductance, categorized as high (H) and low (L). In contrast, configuration **2b** is characterized solely by a high level of conductance. Meanwhile, configuration **3b** features three conductance levels, classified as high, medium (M), and low.

S8. π- π stacking

To evaluate the π-stacking impact on the conductance, many dimer configurations were considered. After sliding one molecule on top of the other one in **2a**, **2b,** the DQI was visible only in **2a** (see Figures S72 and S73 respectively).

In **2a**, the DQI appeared only at the following sliding positions $X = 7.5\ A^{\circ}$and $X = 9.5\ A^{\circ}$.

S8.1. 2a

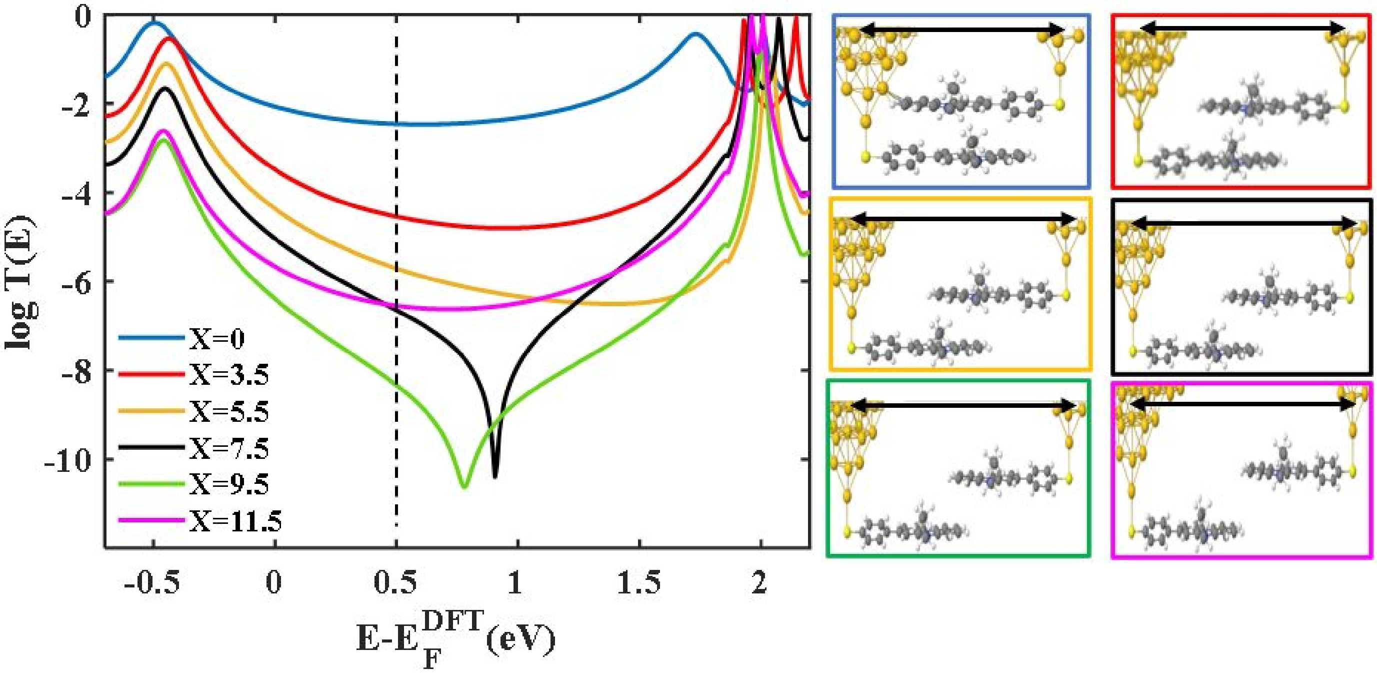

Figure S72. The transmission coefficient of 2**a** for different horizontal sliding positions (X = 0 – 11.5 Å).

T(E) values are taken at $E - E_F^{DFT} = 0.5$ eV .

S8.2. 2b

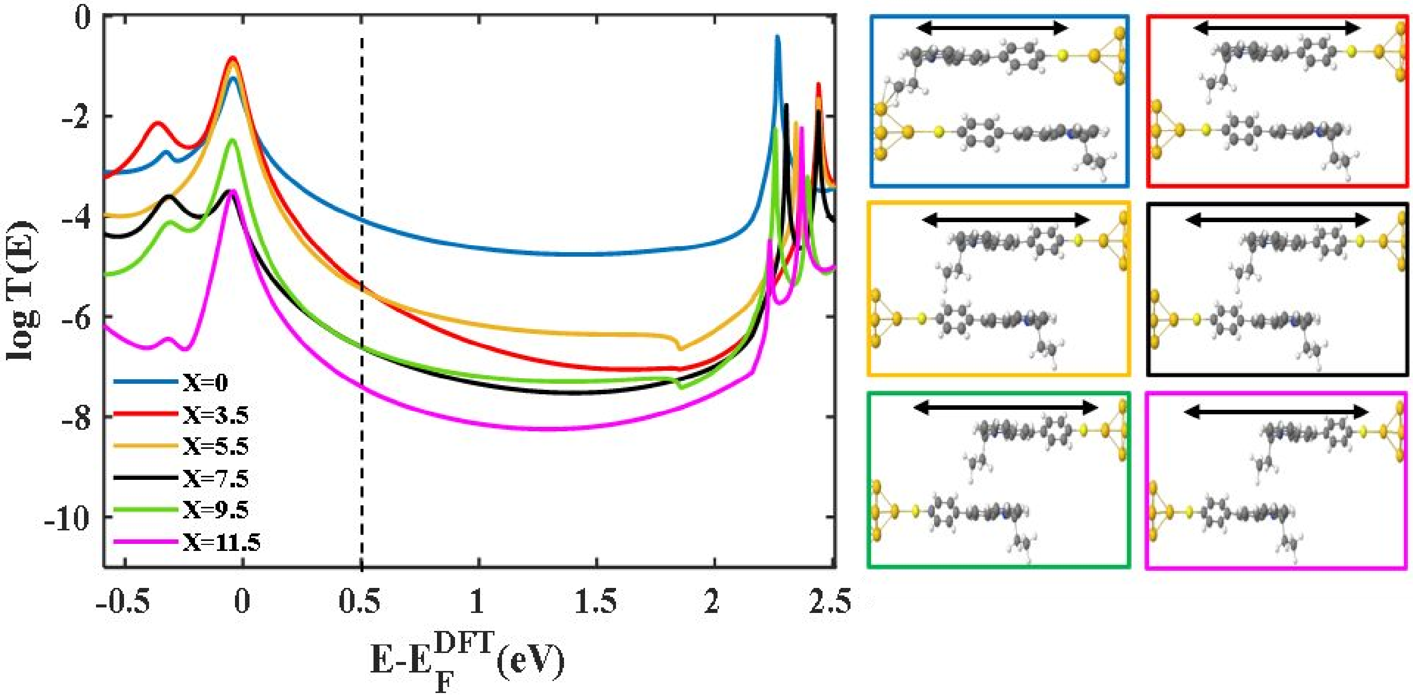

Figure S73. The transmission coefficient of 2**b** for different horizontal sliding positions (X = 0 – 11.5 Å).

T(E) values are taken at $E - E_F^{DFT} = 0.5$ eV .

Table S4. Comparison between experimental and theory conductance fluctuation (**ΔG**).

<table>
<tr><th colspan="8">2a</th></tr>
<tr><td colspan="3">Theory at E_F = -0.3 eV</td><td>Exp</td><td colspan="3">Theory at E_F = 0.8eV</td><td>Exp</td></tr>
<tr><td colspan="2">Conductance Level</td><td>Theo-ΔG</td><td>Exp-ΔG</td><td colspan="2">Conductance Level</td><td>Theo-ΔG</td><td>Exp-ΔG</td></tr>
<tr><td>HC</td><td>-2.43</td><td rowspan="2">1.7</td><td rowspan="2">0.8</td><td>HC</td><td>-5.3</td><td rowspan="2">1.1</td><td rowspan="2">0.8</td></tr>
<tr><td>LC</td><td>-4.10</td><td>LC</td><td>-6.4</td></tr>
</table>

S9. Molecular design

To determine the ideal alkane length to link the carbazole groups we applied DFT/GGA calculations. By comparing the energy difference between the open and closed forms (unsubstituted carbazole), the results predicted that n = 2 (4 carbon atoms) would be energetically favored. However, this prediction changes to n=1 (3 carbon atoms) when we performed a similar calculation for a substituted carbazole, e.g. the dithiol analog of **1a** (Tables S5 and S6).

Unsubstituted bicarbazole

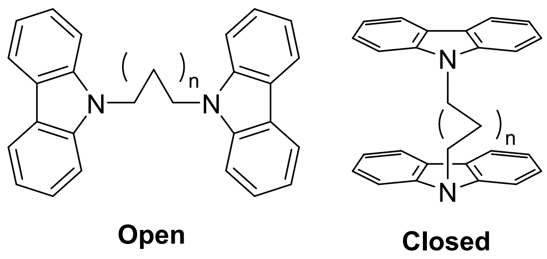

n = 0, 1, 2, 3

Table S5. The ideal alkane length to link the carbazole-core groups

DFT	ΔE(kJ/mol)
n	GGA/DFT
n=0	15.75
n=1	28.15
n=2	46.25
n=3	32.80

Substituted bicarbazole

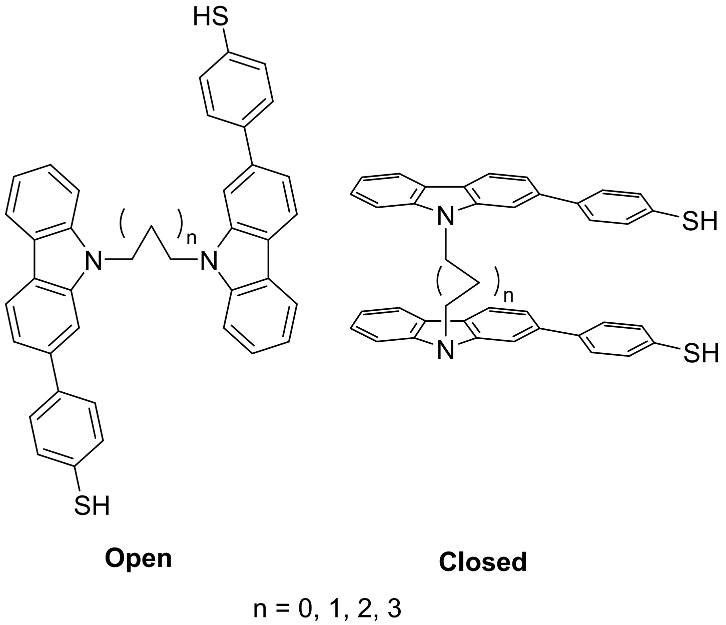

Table S6. The ideal alkane length to link the carbazole groups for the thiol analog of **1a.**

DFT	ΔE(kJ/mol)	
**n**	**unsubstituted**	**substituted**
n=0	73.90	70
n=1	0.15	72
n=2	11.39	20.2
n=3	24	43.4

References

(1) Barata, P. D.; Prata, J. V. New entities for sensory chemistry based on calix[4]arene-carbazole conjugates: from synthesis to applications. *Supramolecular Chemistry* **2013**, *25* (12), 782-797. DOI: 10.1080/10610278.2013.804185.

(2) Sander, F.; Peterle, T.; Ballav, N.; Wrochem, F. v.; Zharnikov, M.; Mayor, M. Loops versus Stems: Benzylic Sulfide Oligomers Forming Carpet Type Monolayers. *The Journal of Physical Chemistry C* **2010**, *114* (9), 4118-4125. DOI: 10.1021/jp911879x.

(3) Grunder, S.; Huber, R.; Wu, S.; Schönenberger, C.; Calame, M.; Mayor, M. Oligoaryl Cruciform Structures as Model Compounds for Coordination-Induced Single-Molecule Switches. *European Journal of Organic Chemistry* **2010**, *2010* (5), 833-845. DOI: 10.1002/ejoc.200901150.

(4) Krause, L.; Herbst-Irmer, R.; Sheldrick, G. M.; Stalke, D. Comparison of silver and molybdenum microfocus X-ray sources for single-crystal structure determination. *Journal of Applied Crystallography* **2015**, *48* (1), 3-10. DOI: doi:10.1107/S1600576714022985.

(5) Sheldrick, G. A short history of SHELX. *Acta Crystallogr., Sect. A* **2008**, *64* (1), 112-122. DOI: doi:10.1107/S0108767307043930.

(6) Sheldrick, G. SHELXT - Integrated space-group and crystal-structure determination. *Acta Crystallographica Section A* **2015**, *71* (1), 3-8. DOI: doi:10.1107/S2053273314026370.

(7) Sheldrick, G. Crystal structure refinement with SHELXL. *Acta Crystallographica Section C* **2015**, *71* (1), 3-8. DOI: doi:10.1107/S2053229614024218.

(8) Dolomanov, O. V.; Bourhis, L. J.; Gildea, R. J.; Howard, J. A. K.; Puschmann, H. OLEX2: a complete structure solution, refinement and analysis program. *Journal of Applied Crystallography* **2009**, *42*, 339-341. DOI: 10.1107/s0021889808042726.

(9) Cabosart, D.; El Abbassi, M.; Stefani, D.; Frisenda, R.; Calame, M.; van der Zant, H. S. J.; Perrin, M. L. A reference-free clustering method for the analysis of molecular break-junction measurements. *Applied Physics Letters* **2019**, *114* (14). DOI: 10.1063/1.5089198.

(10) Lemmer, M.; Inkpen, M. S.; Kornysheva, K.; Long, N. J.; Albrecht, T. Unsupervised vector-based classification of single-molecule charge transport data. *Nature Communications* **2016**, *7* (1), 12922. DOI: 10.1038/ncomms12922.

(11) Zotti, L. A.; Bednarz, B.; Hurtado-Gallego, J.; Cabosart, D.; Rubio-Bollinger, G.; Agrait, N.; van der Zant, H. S. J. Can One Define the Conductance of Amino Acids? *Biomolecules* **2019**, *9* (10), 580.

(12) Artacho, E.; Anglada, E.; Diéguez, O.; Gale, J. D.; García, A.; Junquera, J.; Martin, R. M.; Ordejón, P.; Pruneda, J. M.; Sánchez-Portal, D.; et al. The SIESTA method; developments and applicability. *Journal of Physics: Condensed Matter* **2008**, *20* (6), 064208. DOI: 10.1088/0953-8984/20/6/064208.

(13) Soler, J. M.; Artacho, E.; Gale, J. D.; Garcia, A.; Junquera, J.; Ordejon, P.; Sanchez-Portal, D. The SIESTA method for ab initio order-N materials simulation. *Journal of Physics: Condensed Matter* **2002**, *14* (11), 2745-2779. DOI: 10.1088/0953-8984/14/11/302.

(14) Berland, K.; Cooper, V. R.; Lee, K.; Schröder, E.; Thonhauser, T.; Hyldgaard, P.; Lundqvist, B. I. van der Waals forces in density functional theory: a review of the vdW-DF method. *Reports on Progress in Physics* **2015**, *78* (6), 066501. DOI: 10.1088/0034-4885/78/6/066501.

(15) Hermann, J.; Tkatchenko, A. Density Functional Model for van der Waals Interactions: Unifying Many-Body Atomic Approaches with Nonlocal Functionals. *Physical Review Letters* **2020**, *124* (14), 146401. DOI: 10.1103/PhysRevLett.124.146401.

(16) Sabater, C.; Untiedt, C.; Palacios, J. J.; Caturla, M. J. Mechanical Annealing of Metallic Electrodes at the Atomic Scale. *Physical Review Letters* **2012**, *108* (20), 205502. DOI: 10.1103/PhysRevLett.108.205502.

(17) Fernández, M. A.; Sabater, C.; Dednam, W.; Palacios, J. J.; Calvo, M. R.; Untiedt, C.; Caturla, M. J. Dynamic bonding of metallic nanocontacts: Insights from experiments and atomistic simulations. *Physical Review B* **2016**, *93* (8), 085437. DOI: 10.1103/PhysRevB.93.085437.

(18) Boys, S. F.; and Bernardi, F. The calculation of small molecular interactions by the differences of separate total energies. Some procedures with reduced errors. *Molecular Physics* **1970**, *19* (4), 553-566. DOI: 10.1080/00268977000101561.

(19) Jones, R. O.; Gunnarsson, O. The density functional formalism, its applications and prospects. *Reviews of Modern Physics* **1989**, *61* (3), 689-746. DOI: 10.1103/RevModPhys.61.689.

(20) Parr, R. G.; Weitao, Y. *Density-Functional Theory of Atoms and Molecules*; Oxford University Press, 1995. DOI: 10.1093/oso/9780195092769.001.0001.

(21) Ferrer, J.; Lambert, C. J.; Garcia-Suarez, V. M.; Manrique, D. Z.; Visontai, D.; Oroszlany, L.; Rodriguez-Ferradas, R.; Grace, I.; Bailey, S. W. D.; Gillemot, K.; et al. GOLLUM: a next-generation simulation tool for electron, thermal and spin transport. *New Journal of Physics* **2014**, *16*. DOI: 10.1088/1367-2630/16/9/093029.
